# Supplementary material for: Impact of matching error on linked mortality outcome in a data linkage of secondary mental health data with Hospital Episode Statistics (HES) and mortality records in South East London: a cross-sectional study
Source: BMJ Open. 2020 Jul 7;10(7):e035884. doi: 10.1136/bmjopen-2019-035884 (PMC7342822; doi:10.1136/bmjopen-2019-035884)
Supplement: Supplementary data [file bmjopen-2019-035884supp002.pdf]

Table 2. Logistic regression analysis examining administrative, socio-demographic, and clinical associations with an optimal match.

Variable	Total Population (N=241,890)	Optimal Match (*n*=203,552 84.15%)	Non-optimal Match (*n*=38,338 15.85%)	OR (95% CI)	aOR[1] (95% CI)
**Sociodemographic variables**					
Age: mean (SD)	43.70 (22.89)	44.68 (23.01)	38.47 (21.52)	**0.987 (0.987-0.988)****	**0.985 (0.984-0.986)****
Male Sex: *n* (%)	119634 (49.46)	99187 (48.73)	20447 (53.38)	**1.20 (1.18-1.23)****	**1.14 (1.12-1.17)****
Patient deceased: *n* (%)	28790 (11.90)	25629 (12.59)	3161 (8.25)	**0.63 (0.61-0.65)****	0.95 (0.91-1.00)
Ethnicity coded: *n* (%)					
British, Irish, or any other white ethnic groups	128547 (59.32)	109139 (59.71)	19408 (57.25)	(reference)	(reference)
Mixed	6306 (2.91)	5168 (2.83)	1138 (3.36)	**1.24 (1.16-1.32)****	1.00 (0.93-1.07)
Indian, Pakistani, Bangladeshi, or 'other Asian'	9745 (4.50)	8348 (4.57)	1397 (4.12)	**0.94 (0.89-0.99)***	**0.90 (0.85-0.96)***
Caribbean, African, or any 'other black'	37052 (17.10)	31115 (17.02)	5937 (17.51)	**1.07 (1.04-1.11)****	0.97 (0.93-1.00)
Other	14728 (6.80)	12248 (6.70)	2480 (7.32)	**1.14 (1.09-1.19)****	**1.08 (1.03-1.13)***
Not stated	20319 (9.38)	16778 (9.18)	3541 (10.45)	**1.19 (1.14-1.23)****	**1.04 (1.00-1.09)***
Resident in SLaM catchment area: *n* (%)	172567 (73.34)	145594 (73.34)	26973 (73.37)	1.00 (0.98-1.03)	**1.13 (1.09-1.16)****
Quartiles of neighbourhood deprivation: *n* (%)					
1st (most deprived)	58789 (25.15)	49830 (25.24)	8959 (24.67)	(reference)	(reference)
2nd	58146 (24.87)	48785 (24.71)	9361 (25.78)	**1.07 (1.03-1.10)****	1.01 (0.98-1.05)
3rd	58342 (24.96)	49200 (24.92)	9142 (25.18)	**1.03 (1.00-1.07)***	0.97 (0.94-1.01)
4th (least deprived)	58488 (25.02)	49638 (25.14)	8850 (24.37)	0.99 (0.96-1.02)	**0.92 (0.89-0.96)****
**Clinical variables**					
Referral status in past 2 years: *n* (%)					
Accepted	11061 (4.59)	9445 (4.65)	1616 (4.23)	(reference)	(reference)
Discharged	41227 (17.09)	35007 (17.25)	6220 (16.27)	1.04 (0.98-1.10)	**1.11 (1.04-1.18)***
Rejected	8559 (3.55)	7060 (3.48)	1499 (3.92)	**1.24 (1.06-1.18)****	**1.10 (1.01-1.21)***
No referral in 2 year prior to linkage	180330 (74.77)	151442 (74.62)	28888 (75.58)	**1.11 (1.06-1.18)****	**1.24 (1.17-1.32)****
Primary diagnosis ever: *n (*%)					
F00-F09: organic, including symptomatic, mental disorders	24095 (9.96)	21191 (10.41)	2904 (7.57)	**0.71 (0.68-0.73)****	**1.31 (1.24-1.39)****
F10-F19: mental and behavioural disorders due to psychoactive substance use	25530 (10.55)	21124 (10.38)	4406 (11.49)	**1.12 (1.08-1.16)****	**1.17 (1.12-1.22)****
F20-F29:schizophrenia, schizotypal and delusional disorders	16889 (6.98)	14328 (7.04)	2561 (6.68)	**0.95 (0.91-0.99)***	1.05 (0.99-1.11)

F30-F39: mood (affective) disorders	39799 (16.45)	34512 (16.95)	5287 (13.79)	**0.78 (0.76-0.81)****	**0.88 (0.85-0.92)****
F40-F49: neurotic, stress-related and somatoform disorders	30943 (12.79)	26729 (13.13)	4214 (10.99)	**0.82 (0.79-0.85)****	**0.83 (0.79-0.86)****
F50-F59: behavioural syndromes associated with physiological disturbances and physical factors	8086 (3.34)	6634 (3.26)	1452 (3.79)	**1.17 (1.10-1.24)****	**1.12 (1.05-1.19)***
F60-F69: disorders of adult personality and behaviour	6509 (2.69)	5521 (2.71)	988 (2.58)	0.95 (0.89-1.02)	0.98 (0.91-1.05)
F70-F79: mental retardation	2810 (1.16)	2448 (1.20)	362 (0.94)	**0.78 (0.70-0.87)****	**0.82 (0.73-0.92)****
F80-F89: disorders of psychological development	6804 (2.81)	5673 (2.79)	1131 (2.95)	1.06 (0.99-1.13)	**0.76 (0.70-0.81)****
F90-F98: behavioural and emotional disorders with onset usually occurring in childhood and adolescence	15931 (6.59)	12877 (6.33)	3054 (7.97)	**1.28 (1.23-1.34)****	0.96 (0.91-1.00)
Other diagnosis	109854 (45.41)	92623 (45.50)	17231 (44.94)	**0.98 (0.96-1.00)***	**0.96 (0.94-0.99)****
Quartiles of face to face contacts: *n* (%)					
1st (least face to face contact)	68150 (28.17)	56507 (27.76)	11643 (30.37)	(reference)	(reference)
2nd	58128 (24.03)	48702 (23.93)	9426 (24.59)	**0.94 (0.91-0.97)****	1.01 (0.97-1.04)
3rd	54781 (22.65)	46311 (22.75)	8470 (22.09)	**0.89 (0.86-0.92)****	**0.95 (0.91-0.99)***
4th (most face to face contact)	60831 (25.15)	52032 (25.56)	8799 (22.95)	**0.82 (0.80-0.85)****	**0.89 (0.85-0.93)****
Inpatient bed days: *n* (%)					
None (0)	216890 (89.66)	182362 (89.59)	34528 (90.06)	(reference)	(reference)
Low (1-2 days)	1327 (0.55)	1106 (0.54)	221 (0.58)	1.06 (0.91-1.22)	1.06 (0.91-1.24)
Moderate (3-31 days)	9064 (3.75)	7607 (3.74)	1457 (3.80)	1.01 (0.96-1.07)	1.05 (0.98-1.12)
High (32+ days)	14609 (6.04)	12477 (6.13)	2132 (5.56)	**0.90 (0.86-0.95)****	1.00 (0.95-1.06)

Note. Optimal match data missing for *n*=6,808 (2.75%) matched records. **p*<0.05. ***p*<0.001. [1]adjusted for all other variables listed in the table. Missing data: sex (*n*=34); ethnicity (*n*=25,193); resident in local catchment area (*n*=6,604); quartiles of neighbourhood deprivation (*n*=8,125); referral status (*n*=713).
