# Supplementary material for: Impact of matching error on linked mortality outcome in a data linkage of secondary mental health data with Hospital Episode Statistics (HES) and mortality records in South East London: a cross-sectional study
Source: BMJ Open. 2020 Jul 7;10(7):e035884. doi: 10.1136/bmjopen-2019-035884 (PMC7342822; doi:10.1136/bmjopen-2019-035884)
Supplement: Supplementary data [file bmjopen-2019-035884supp003.pdf]

Table 3. Logistic regression analysis examining the association between sociodemographic factors and availability of NHS number for matching.

Variable	Total Population (N=265,300)	NHS Number Present (*n*=259,545 97.83%)	NHS Number Missing (*n*=5,755 2.17%)	OR (95% CI)	aOR[1] (95% CI)
**Sociodemographic variables**					
Age: mean (SD)	43.40 (22.69)	43.49 (22.79)	39.19 (16.57)	**1.009 (1.008-1.010)****	**1.010 (1.008-1.012)****
Male Sex: *n* (%)	132730 (50.04)	126085 (49.74)	3645 (63.47)	**0.57 (0.54-0.60)****	**0.60 (0.56-0.66)****
Ethnicity: *n* (%)					
British, Irish, or any other white ethnic groups	138495(58.54)	136495 (58.69)	2265 (50.99)	(reference)	(reference)
Mixed	6853 (2.90)	6782 (2.92)	71 (1.60)	**1.59 (1.25-2.01)****	**1.60 (1.16-2.19)***
Indian, Pakistani, Bangladeshi, or 'other Asian'	10889 (4.60)	10586 (4.56)	303 (6.82)	**0.58 (0.51-0.66)****	**0.44 (0.38-0.51)****
Caribbean, African, or 'other black'	40725 (17.21)	40008 (17.24)	717 (16.14)	0.93 (0.85-1.01)	**0.73 (0.65-0.82)****
Other	16650 (7.04)	16120 (6.94)	530 (11.93)	**0.51 (0.46-0.56)****	**0.45 (0.39-0.51)****
Not stated	22961 (9.71)	22405 (9.65	556 (12.52)	**0.67 (0.61-0.74)****	**0.58 (0.51-0.65)****
Resident in SLaM Catchment Area: *n* (%)	187773 (73.42)	185393 (73.51)	2380 (66.93)	**1.37 (1.28-1.47)****	**1.15 (1.04-1.26)***
Quartiles of neighbourhood deprivation: *n* (%)					
1st (most deprived)	63476 (25.03)	62832 (25.10)	644 (19.93)	(reference)	(reference)
2nd	63452 (25.02)	62605 (25.01)	847 (26.21)	**0.76 (0.68-0.84)****	**0.66 (0.59-0.75)****
3rd	63449 (25.02)	62557 (24.99)	892 (27.60)	**0.72 (0.65-0.80)****	**0.68 (0.60-0.77)****
4th (least deprived)	63221 (24.93	62371 (24.91)	849 (26.27)	**0.75 (0.68-0.83)****	**0.70 (0.61-0.79)****

Note. **p*<0.05. ***p*<0.001. [1]adjusted for all other variables listed in the table. Missing data: age (*n*=275); sex (*n*=55); ethnicity (*n*=28,727); quartiles of neighbourhood deprivation (*n*=11,702).
