# Supplementary material for: Impact of matching error on linked mortality outcome in a data linkage of secondary mental health data with Hospital Episode Statistics (HES) and mortality records in South East London: a cross-sectional study
Source: BMJ Open. 2020 Jul 7;10(7):e035884. doi: 10.1136/bmjopen-2019-035884 (PMC7342822; doi:10.1136/bmjopen-2019-035884)
Supplement: Supplementary data [file bmjopen-2019-035884supp004.pdf]

**Table 4.** Logistic regression analysis examining the association between sociodemographic factors and availability of date of birth for matching.

| Variable | Total Population (N=265,300) | Date of Birth Present (*n*=265,010 99.89%) | Date of Birth Missing (*n*=290 0.11%) | OR (95% CI) | aOR[1] (95% CI) |
|---|---|---|---|---|---|
| **Sociodemographic variables** | | | | | |
| Age: mean (SD) | 43.40 (22.69) | 43.40 (22.69) | 32.93 (20.49) | 1.025 (0.997-1.054) | 1.033 (0.974-1.096) |
| Male Sex: *n* (%) | 132730 (50.04) | 132585 (50.04) | 145 (50.17) | 0.99 (0.79-1.25) | 1.78 (0.29-10.77) |
| Ethnicity: *n* (%) | | | | | |
| British, Irish, or any other white ethnic groups | 138495(58.54) | 138440 (58.55) | 55 (50.00) | (reference) | (reference) |
| Mixed | 6853 (2.90) | 6853 (2.90) | 0 (0.00) | ---- | ---- |
| Indian, Pakistani, Bangladeshi, or 'other Asian' | 10889 (4.60) | 10877 (4.60) | 12 (10.91) | **0.36 (0.19-0.67)*** | 0.08 (0.01-1.22) |
| Caribbean, African, or 'other black' | 40725 (17.21) | 40710 (17.22) | 15 (13.64) | 1.08 (0.61-1.91) | ---- |
| Other | 16650 (7.04) | 16632 (7.03) | 18 (16.36) | **0.37 (0.22-0.63)**** | 0.10 (0.01-1.73) |
| Not stated | 22961 (9.71) | 22951 (9.71) | 10 (9.09) | 0.91 (046-1.79) | 0.11 (0.01-1.23) |
| Resident in SLaM Catchment Area: *n* (%) | 187773(73.42) | 187661 (73.42) | 112 (73.68) | 0.99 (0.69-1.42) | 3.23 (0.46-22.53) |
| Quartiles of neighbourhood deprivation: *n* (%) | | | | | |
| 1st (most deprived) | 63476 (25.03) | 63428 (25.03) | 48 (33.33) | (reference) | (reference) |
| 2nd | 63452 (25.02) | 63427 (25.03) | 25 (17.36) | **1.92 (1.18-3.11)*** | 1.43 (0.12-16.75) |
| 3rd | 63449 (25.02) | 63405 (25.02) | 44 (30.56) | 1.09 (0.72-1.64) | 1.26 (0.10-15.36) |
| 4th (least deprived) | 63221 (24.93 | 63194 (24.93) | 27 (18.75) | **1.77 (1.11-2.84)*** | 1.23 (0.10-15.00) |

**Note.** **p*<0.05. ***p*<0.001. [1]adjusted for all other variables listed in the table. Missing data: age (*n*=275); sex (*n*=55); ethnicity (*n*=28,727); quartiles of neighbourhood deprivation (*n*=11,702).
