# Supplementary material for: Impact of matching error on linked mortality outcome in a data linkage of secondary mental health data with Hospital Episode Statistics (HES) and mortality records in South East London: a cross-sectional study
Source: BMJ Open. 2020 Jul 7;10(7):e035884. doi: 10.1136/bmjopen-2019-035884 (PMC7342822; doi:10.1136/bmjopen-2019-035884)
Supplement: Author's manuscript [file bmjopen-2019-035884.draft_revisions.pdf]

BMJ Open is committed to open peer review. As part of this commitment we make the peer review history of every article we publish publicly available.

When an article is published we post the peer reviewers' comments and the authors' responses online. We also post the versions of the paper that were used during peer review. These are the versions that the peer review comments apply to.

The versions of the paper that follow are the versions that were submitted during the peer review process. They are not the versions of record or the final published versions. They should not be cited or distributed as the published version of this manuscript.

BMJ Open is an open access journal and the full, final, typeset and author-corrected version of record of the manuscript is available on our site with no access controls, subscription charges or pay-per-view fees (http://bmjopen.bmj.com).

If you have any questions on BMJ Open's open peer review process please email info.bmjopen@bmj.com

BMJ Open

# The Impact of Matching Error on Linked Mortality Outcome in a Data Linkage of Secondary Mental Health Data with Hospital Episode Statistics (HES) and Mortality records

| | |
|---|---|
| Journal: | *BMJ Open* |
| Manuscript ID | bmjopen-2019-035884 |
| Article Type: | Original research |
| Date Submitted by the Author: | 20-Nov-2019 |
| Complete List of Authors: | Jewell, Amelia; South London and Maudsley NHS Foundation Trust, Maudsley Biomedical Research Centre (BRC)<br>Broadbent, Matthew; South London and Maudsley NHS Foundation Trust, Maudsley Biomedical Research Centre (BRC)<br>Hayes, Richard; Institute of Psychiatry, Kings College London, Psychological Medicine<br>Gilbert, Ruth; UCL Institute of Child Health, Centre for Paediatric Epidemiology and Biostatistics<br>Stewart, Robert; King's College London, Institute of Psychiatry<br>Downs, Johnny; Kings College London, Institute of Psychiatry, Psychology and Neuroscience |
| Keywords: | Health informatics < BIOTECHNOLOGY & BIOINFORMATICS, MENTAL HEALTH, PSYCHIATRY |
| | |

SCHOLARONE™
Manuscripts

*I, the Submitting Author has the right to grant and does grant on behalf of all authors of the Work (as defined in the below author licence), an exclusive licence and/or a non-exclusive licence for contributions from authors who are: i) UK Crown employees; ii) where BMJ has agreed a CC-BY licence shall apply, and/or iii) in accordance with the terms applicable for US Federal Government officers or employees acting as part of their official duties; on a worldwide, perpetual, irrevocable, royalty-free basis to BMJ Publishing Group Ltd ("BMJ") its licensees and where the relevant Journal is co-owned by BMJ to the co-owners of the Journal, to publish the Work in this journal and any other BMJ products and to exploit all rights, as set out in our licence.*

*The Submitting Author accepts and understands that any supply made under these terms is made by BMJ to the Submitting Author unless you are acting as an employee on behalf of your employer or a postgraduate student of an affiliated institution which is paying any applicable article publishing charge ("APC") for Open Access articles. Where the Submitting Author wishes to make the Work available on an Open Access basis (and intends to pay the relevant APC), the terms of reuse of such Open Access shall be governed by a Creative Commons licence – details of these licences and which Creative Commons licence will apply to this Work are set out in our licence referred to above.*

*Other than as permitted in any relevant BMJ Author's Self Archiving Policies, I confirm this Work has not been accepted for publication elsewhere, is not being considered for publication elsewhere and does not duplicate material already published. I confirm all authors consent to publication of this Work and authorise the granting of this licence.*

For peer review only

# The Impact of Matching Error on Linked Mortality Outcome in a Data Linkage of Secondary Mental Health Data with Hospital Episode Statistics (HES) and Mortality records

Amelia Jewell[1*], Matthew Broadbent[1], Richard D Hayes[2], Ruth Gilbert[3], Robert Stewart[1,2], and Johnny Downs[1,2]

* Corresponding author contact information:

Amelia Jewell, Biomedical Research Centre (BRC) Nucleus (PO92), Kings College London, DeCrespigny Park, London, SE5 8AF, United Kingdom. Telephone: +44 20 3228 8554; E-mail: amelia.jewell@slam.nhs.uk

**Author details:**

[1] South London and Maudsley NHS Foundation Trust, London, UK. [2] Institute of Psychiatry, Psychology and Neuroscience, King's College London, London, UK. [3] University College London Great Ormond Street Institute of Child Health.



For peer review only - http://bmjopen.bmj.com/site/about/guidelines.xhtml

## ABSTRACT

**Objectives:** Linkage of electronic health records (EHRs) to Hospital Episode Statistics (HES)-Office for National Statistics (ONS) mortality data has provided compelling evidence for lower life expectancy in people with severe mental illness. However, linkage error may underestimate these estimates. Using a clinical sample (N=265,300) of individuals accessing mental health services, we examined potential biases introduced through missed matching, and examined the impact on the association between clinical disorders and mortality.

**Setting:** The South London and Maudsley NHS Foundation Trust (SLaM) is a secondary mental health care provider in London. A de-identified version of SLaM's EHR was available via the Clinical Record Interactive Search (CRIS) system linked to HES-ONS mortality records.

**Participants:** Records from SLaM for patients active between January 2006 and December 2016.

**Outcome measures:** Two sources of death data were available for SLaM participants: accurate and contemporaneous date of death via local batch tracing (gold standard), and date of death via linked HES-ONS mortality data. The effect of linkage error on mortality estimates was evaluated by comparing sociodemographic and clinical risk factor analyses using gold standard death data, against HES-ONS mortality records.

**Results:** Of the total sample, 93.74% were successfully matched to HES-ONS records. We found a number of statistically significant administrative, socio-demographic, and clinical differences between matched and unmatched records. Of note, schizophrenia diagnosis showed a significant association with higher mortality using gold standard data (OR=1.08; 95% CI=1.01, 1.15; $p$=0.02), but not in HES-ONS data (OR=1.05; 95% CI=0.98, 1.13; $p$=0.16). Otherwise little change was found in the strength of associated risk factors and mortality, after accounting for missed matching bias.

**Conclusions:** Despite significant clinical and socio-demographic differences between matched and unmatched records, changes in mortality estimates were minimal. However, researchers and policy analysts using HES-ONS linked resources should be aware that administrative linkage processes can introduce error.




For peer review only - http://bmjopen.bmj.com/site/about/guidelines.xhtml

**Strengths and limitations of this study:**

- The findings of our study demonstrate that although there are significant differences between matched and unmatched records, these did not significantly change the relative differences in mortality according to type of psychiatric disorder.

- The findings from this study are novel, in that data linkage error has not previously been evaluated in a routine mental health database and this may be useful for other researchers looking to conduct research using electronic health records linked to HES-ONS mortality data by NHS Digital.

- A significant strength of the study is our access to the gold standard death data within the mental health record, which can be used to compare with the linked mortality data; this is useful as the mental health discrepancy in mortality is a particular focus on issues of parity/ inequality for health policy.

- Results may be generalisable to other NHS cohorts being linked to HES-ONS Mortality data via NHS Digital as national standard matching methodology was used for the data linkage and the quality and type of administrative data available for matching is likely to be similar within most NHS Trusts.

- False matches were not examined; therefore, data linkage error within the linked dataset may be higher.



For peer review only

For peer review only - http://bmjopen.bmj.com/site/about/guidelines.xhtml

**INTRODUCTION**

Individuals with mental health disorders have substantially lower life expectancy in comparison to those without mental health disorders [1,2]. The United Kingdom (UK) government has been working to reduce the mortality gap between those with and without mental health disorders by implementing policies aimed at improving the physical health of individuals with mental illness [3]. To inform changes in health policy it is essential that we have good data available on health (physical and mental) and mortality in order to understand the trends and underlying mechanisms.

Electronic health record (EHR) data from the South London and Maudsley NHS Foundation Trust (SLaM) Clinical Record Interactive Search (CRIS) system [4] linked to Hospital Episode Statistics (HES) and death certificate data from the Office for National Statistics (ONS) have previously been utilised to provide evidence of the mental health mortality gap and investigate potential underlying mechanisms [5-9]. Data linkages such as that between CRIS and HES-ONS mortality resources are an important tool for increasing the use of existing data resources to support research [10]. However, the success of any data linkage and the quality of the subsequent linked dataset rely on multiple factors, including the data quality and linkage methodology. The two main types of linkage error which can occur during the data linkage process are false matches (i.e. false positives) and missed matches (i.e. false negatives). Errors in data linkage may result in systematic bias in reported outcomes [11]. False positives in data linkages can dilute the association between variables [12] whereas false negatives can cause an underestimation of the risk outcome [13]; for example, a US study found that linking on an infant's medical insurance record, as opposed to the mother's, led to a significant underestimation in infant mortality due to the number of missed matches [14].

There are a number of approaches to evaluating linkage quality; for example by comparing characteristics of linked and unlinked records to identify potential sources of bias [12]. Previous studies have identified significant differences in socio-demographic characteristics between matched and unmatched samples in data linkages, including sex [15-21], age [15,19,22-27], ethnicity [16,19,20-22,26-32], socioeconomic status [20,27,33,34], marital status [15,22,28,35], and level of education [20,34,35]. Providing some indication that linked data may not always be representative of the population of interest [12]. Reference or 'gold standard' datasets in which the true match status is known can be used to evaluate the impact of data linkage error by quantifying missed or false matches [36]. Gold standard datasets can enable identification of both missed matches and false matches by providing the true match status of a record; however, gold standard data either do not exist or are rarely available to researchers using linked data due to issues around governance and data protection [12].

If linked data are being used to inform health policy it is important that the effect of linkage error is estimated. At present, for example, it is not clear whether any potential error in the linkage



For peer review only - http://bmjopen.bmj.com/site/about/guidelines.xhtml

between CRIS and HES-ONS mortality data has a bearing on the research being conducted using the linked data. It was therefore our aim to investigate the effect of linkage error on the prediction of a linked outcome measure in this mental health population by, firstly, examining the administrative, socio-demographic, and clinical characteristics of matched and unmatched records, and secondly, by comparing the prediction of linked mortality outcome with gold-standard death data provided by the local mental health record.

## METHODS

*The Mental Health Dataset - CRIS*

The Maudsley Biomedical Research Centre (BRC) CRIS system, described previously in detail [4,37], is a deidentified database of electronic medical records for mental health service users within SLaM: one of the largest providers of secondary mental health care in Europe, providing care predominately for the London boroughs of Lambeth, Southwark, Croydon, and Lewisham [4]. Records from CRIS have previously been linked to a number of external databases including the National Pupil Database [38], the Thames Cancer Registry [39], and General Practice (GP) data [40].

All individuals (children and adults) who were active in SLaM between 1st January 2006 and 20th December 2016, not including IAPT (Improving Access to Psychological Therapies) services, were included in the current study. Exposure variables (Table 1) were collected from structured fields within the CRIS system and included socio-demographic (e.g. age, sex, ethnicity, and mortality), clinical (e.g. mental health diagnosis, referral status, level of service use), and administrative variables (i.e. patient identifiers available for linkage).

Mortality data from CRIS provided the ‘gold standard’ death data using both formal and informal notification sources [41]. All-cause mortality data is entered into the SLaM EHR either by the care team directly (for example after being informed by family members) or via automatic tracing, whereby the SLaM Trust run a demographic batch trace weekly for all patients (past or current) against the NHS Summary Care Record (SCR). This returns date of death for those patients who have died (Figure 1). The SCR is an electronic record of important patient information created from GP medical records which can be seen and used by any authorised staff in areas of the health system who are involved in a patients’ direct care. The SCR is administered by NHS Digital [42]. Date of death in the SCR is entered at the point of occurrence, i.e. the death may not have been formally registered yet.

*The Hospital Episode Statistics and ONS Mortality Dataset*

HES data are a national dataset, governed by NHS Digital, which holds details of all admissions, outpatient appointments, and accident and emergency attendances at NHS hospitals in England [43]. HES data are derived from the Secondary Uses Service (SUS) database which collects submissions



For peer review only - http://bmjopen.bmj.com/site/about/guidelines.xhtml

routinely from all NHS acute hospital and mental health trusts in England. The data accuracy and quality within these submissions are important, especially to NHS Trusts, as they are used to calculate payments for the care provided [44]. HES data are designed to enable secondary use, e.g. for research, and can be linked to other external data sources.

The ONS mortality data consists of the Primary Care Mortality Database (PCMD) which includes date and cause of death for all deaths registered in England and Wales. The data are collected by the ONS and access to the data is managed by NHS Digital [45]. Mortality data from the PCMD are only returned for individuals who have matched and have died; individuals without a mortality record are therefore assumed to be alive.

*Linkage Procedures*

The linkage between CRIS, HES-ONS mortality data under analysis here was conducted in January 2017. Patient Identifiable Information (PII), i.e. date of birth, sex, NHS number, and postcode, for all SLaM records (excluding anybody who had previously opted out of CRIS) was extracted on 20th December 2016. The data linkage was conducted by NHS Digital following deterministic matching procedures. Date of birth, sex, NHS number, and most recently recorded postcode were used as the personal identifiers to match records. Table 2 demonstrates the match ranks used by NHS Digital, with the quality of the matching decreasing from step one to eight.

Initially records are matched to the Personal Demographics Service (PDS) using this method. The PDS is the national electronic database of NHS patient details such as name, address, date of birth, and NHS number [46]. The PDS is also involved in the creation and updating of the NHS SCR, described above. Theoretically, every CRIS record should match to the PDS, even if they do not have any HES episodes or a mortality record. Following matching to the PDS, patients who have opted out of their data being used for secondary purposes, known as 'type 2' opt-outs [47], are removed from the cohort. A report is produced containing details of all the matched records, excluding unmatched records and opt-outs, known as the Flagging Current Status report. Mortality and HES data are then extracted for matched records only (see Figure 2).

Match status outcome measure (i.e. matched vs. missed match) was determined using the Flagging Current Status report following the linkage of CRIS records by NHS Digital. Unmatched records included both records which were not matched (i.e. missed matches) as well as opt-outs.

For those records which were successfully matched, we examined optimal match rank, whereby an optimal match represents a 'perfect' match or a match rank of one (see Table 2), i.e. the records matched on all supplied patient identifiers. The optimal match variable was provided by NHS Digital within the HES data following completion of the data linkage.



For peer review only - http://bmjopen.bmj.com/site/about/guidelines.xhtml

*Patient and Public Involvement*

Patient and public involvement was key to the initial development and ongoing oversight of the CRIS system. The data linkage between CRIS and the HES-ONS mortality data, as well as this project specifically, were approved by the service-user chaired CRIS Oversight Committee (project reference: 17-065). The CRIS, HES-ONS mortality data linkage was also presented to the Data Linkage Service User and Carer Advisory Group [48], a regular meeting of people with lived experience of mental illness, all of whom have an interest in mental health research involving data linkage. The group provide ongoing advice and feedback to researchers conducting projects using the linked CRIS and HES-ONS mortality data.

*Statistical Analysis*

*Step 1: Missed Matches Analysis*

Data were analysed using STATA version 15. Univariable logistic regression was first performed on all administrative, socio-demographic, and clinical characteristics to examine the association with match status outcome for HES-ONS linkage (i.e. those with a positive match on the Flagging Current Status report). Multivariable logistic regression was then performed to identify factors which remained significant predictors of HES-ONS matching after controlling for all other examined variables.

*Step 2: Optimal Match Analysis*

In order to determine which variables predicted an optimal match, univariable logistic regression analysis was performed on a subsample of the total cohort (i.e. only those records which were matched with HES-ONS). Multivariable logistic regression was then performed to identify factors associated with an optimal match outcome after accounting for all other examined variables.

*Step 3: CRIS Derived Mortality Analysis*

In order to examine the effect of potential matching bias on mortality outcome we first examined factors associated with all-cause mortality using the gold standard CRIS-derived death data in univariable and multivariable analyses.

*Step 4: Linked Mortality Analysis*

We then examined predictors of mortality using the linked mortality outcome (i.e. patients with a death date in the HES-ONS matched dataset), in the HES-ONS matched group only, in univariable and multivariable analyses. We used inverse probability weighting calculated from the Step 1 analysis and the optimal match variable to adjust the final model for missed matching bias.



For peer review only - http://bmjopen.bmj.com/site/about/guidelines.xhtml

**RESULTS**

Within the total cohort (N=265,300), 93.7% (*n*=248,698) of patients were successfully matched by NHS Digital to the HES-ONS data via the PDS. The mean age for the matched sample was 43.40 (Range 5 months to 117 years; SD 22.69), at the time the patient identifiers were extracted, with half the sample being male (50.0%). The majority of the matched group (58.5%) were of white ethnicity and were resident in the four London Boroughs serviced by SLaM, i.e. Lambeth, Southwark, Lewisham, and Croydon (73.4%).

*Step 1: Missed Matches Analysis*

Table 3 provides the administrative, socio-demographic, and clinical characteristics of the CRIS sample, according to matched and unmatched status. With regards to the administrative variables examined (i.e. NHS number, date of birth, sex, and postcode present in PII sent to NHS Digital), records which did not have an NHS number or date of birth were not matched (*n*=5755, 2.17%). Records where sex was present in the PII sent to NHS Digital were eight times more likely to be matched than records where sex was not present (OR=8.14; 95% CI=4.66, 14.23; $p<0.001$). Similarly, records where a postcode was present were almost seven times more likely to be matched in comparison to records where no postcode was available for matching (OR=6.68; 95% CI= 6.26, 7.12; $p<0.001$). Patients who had died prior to the PII being extracted, i.e. 20$^{th}$ December 2016, (according to the gold standard CRIS-derived death data) were significantly more likely to match than patients who were alive.

Within the adjusted analysis, we found the likelihood of matching was significantly associated with a number of socio-demographic and clinical factors. Males and patients of non-white ethnicities, i.e. (i) mixed, (ii) Indian, Pakistani, Bangladeshi or 'other Asian', (iii) Caribbean, African, or 'other black', or (iv) other ethnicity, were all significantly less likely to match. Individuals without a stated ethnicity were also significantly less likely to match. Compared to patients in the lowest quartile of deprivation, patients in the second quartile were significantly less likely to match. We found no significant differences between patients in the third and fourth quartiles of deprivation ($p>0.05$).

With regards to clinical variables, patients who had been discharged or who had not had a referral in the two years prior to the PII being extracted were significantly less likely to match than patients who had an accepted referral in the past 2 years. Similarly, patients with the most face to face contact (third and fourth quartiles) were significantly more likely to match, whilst patients in the second quartile of face to face contact were significantly less likely to match. Patients with the highest number of inpatient bed days (32+) were significantly more likely to match than those with no inpatient bed days, whereas patients with a moderate number of inpatient bed days (3-31) were significantly less likely to match than those who no inpatient bed days.



For peer review only - http://bmjopen.bmj.com/site/about/guidelines.xhtml

In terms of diagnosis, patients who had ever received a primary diagnosis within the majority F00-98 ICD-10 diagnosis codes (see Table 3) were all significantly more likely to match than patients who had never received an ICD-10 F diagnosis. However, primary diagnoses of schizophrenia, schizotypal, and delusional disorders (F20-F29) or disorders of adult personality and behaviour (F60-F69) were not significantly associated with match status.

*Step 2: Optimal Match Analysis*

For records which were matched (*n*=248,698), we examined predictors of an optimal match. An optimal match represents a 'perfect match', i.e. where the records were matched on all four patient identifiers provided to NHS Digital (NHS number, sex, date of birth, and postcode). Within the adjusted model (see Table 1 in Supplementary Material) older age, male sex, an 'other' or 'not stated' ethnicity, living in the SLaM catchment area, deprivation, referral status in the two years prior to the linkage, low levels of face to face contact, and a mental health diagnosis were all significantly associated with an optimal match. All-cause mortality and number of inpatient bed days were not significantly associated with optimal match outcome.

*Step 3: CRIS Derived Mortality Analysis*

Table 4 displays clinical and socio-demographic factors associated with mortality using the gold standard CRIS-derived death data. Within the fully adjusted model ($aOR^2$) older age, male sex, deprivation, a primary diagnosis ever of organic, including symptomatic, mental disorders (F00-F09), mental and behavioural disorders due to psychoactive substance use (F10-F19), schizophrenia, schizotypal and delusional disorders (F20-F29), mental retardation (F70-F79), amount of face to face contact, and inpatient bed days were all associated with a significant increased risk of mortality.

Whilst, non-white ethnicity, a primary diagnosis ever of mood (affective) disorders (F30-F39), neurotic, stress-related and somatoform disorders (F40-F49), behavioural syndromes associated with physiological disturbances and physical factors (F50-F59), behavioural and emotional disorders with onset usually occurring in childhood and adolescence (F90-F98), or other diagnoses were all associated with a significant decreased risk of mortality.

*Step 4: Linked Mortality Analysis*

We examined the associations between mortality and socio-demographic and clinical factors using the linked HES-ONS mortality data for the matched group only (*n*=248,698). Within the adjusted model (see Table 5) we found that older age, male sex, and the second, third and fourth (i.e. least deprived) quartiles of deprivation were significantly associated with mortality. With regards to clinical variables, a primary diagnosis of organic, including symptomatic, mental disorders (F00-F09), mental



For peer review only - http://bmjopen.bmj.com/site/about/guidelines.xhtml

and behavioural disorders due to psychoactive substance use (F10-F19), mental retardation (F70-F79), and a low, moderate, or high number of inpatient bed days were all significantly associated with an increased risk of mortality. Similarly, patients with higher levels of face to face contact (2nd and 3rd quartiles) were significantly more likely to have died when compared to patients with the least amount of face to face contact.

Non-white ethnicity, and a primary diagnosis of mood (affective) disorders (F30-F39), neurotic, stress-related and somatoform disorders (F40-F49), behavioural syndromes associated with physiological disturbances and physical factors (F50-F59), disorders of psychological development (F80-F89), and behavioural and emotional disorders with onset usually occurring in childhood and adolescence (F90-F98) were all negatively associated with mortality.

After further adjustments, using the optimal match variable and an inverse probability weighting, calculated during the missed match analysis in Step 1, to account for potential linkage bias, we found these associations with mortality persisted (Table 5).

We compared the output of the Step 3 and Step 4 analyses (Table 4 - aOR[1] and Table 5), i.e. predictors of CRIS-derived mortality and predictors of linked ONS mortality in the matched group (controlling for optimal matching and matching probability). The two final models were largely the same with slight differences in some of the odds ratios. However, there was a difference between the models within the diagnosis variables. In the model predicting CRIS-derived mortality, a primary diagnosis ever of schizophrenia, schizotypal and delusional disorders (F20-F29) was significantly associated with all-cause mortality (OR=1.08; 95% CI=1.01, 1.15; $p$=0.02). Whereas in the model predicting linked mortality in the matched group, a primary diagnosis of F20-F29 was not significantly associated with mortality (OR=1.05; 95% CI=0.98, 1.13; $p$>0.05), despite the odds ratios being virtually identical (1.08 for the CRIS-derived death model compared to 1.05 for the linked death model). The only other difference between the two models was regarding the F60-F69 diagnosis variable. Patients who had ever had a primary diagnosis of a disorder of adult personality and behaviour (F60-F69) were more likely to have died in the CRIS-derived mortality model, whereas they were less likely to have died in the linked mortality model (OR: 1.02 vs. 0.98 respectively); however, neither of these findings were statistically significant.

## DISCUSSION

*Statement of the Principal Findings*

By evaluating NHS electronic health records linked via standard deterministic procedures to HES-ONS mortality data, we found no evidence of substantial bias distorting risk-factor associations with the linked outcome measure analysed (i.e. mortality). Of the 265,300 SLaM records sent to NHS Digital for matching, 93.74% were successfully matched. Despite a number of significant



For peer review only - http://bmjopen.bmj.com/site/about/guidelines.xhtml

administrative, socio-demographic, and clinical differences between records that matched and those that did not, we found minimal effects on the clinical associations with mortality. Matching error did not appear to impact the strength and direction of effects between mortality and a number of socio-demographic and clinical factors using linked mortality data after adjusting for matching probability using an inverse probability weighting statistical technique. Furthermore, after comparing the fully adjusted models predicting gold standard CRIS-derived mortality in the full cohort and linked mortality in the matched group we found that the two models were largely the same with only slight differences in some of the odds ratios.

*Putting the Results in Context*

Consistent with previous research, we found a number of significant socio-demographic differences between the matched and unmatched group including gender, ethnicity, and deprivation. Previous research has demonstrated how linkage error disproportionately affects marginalised people and, therefore, missed matches result in underestimating their needs [16,19-22,26-34]. In comparison to the white ethnic group (i.e. British, Irish, or any other white ethnic groups), we found that individuals of all other recorded ethnicities examined were significantly less likely to match. This is potentially relevant to researchers examining health outcome variations by ethnicity, and those conducting studies within similarly diverse, urban populations [4].

This is one of the first studies to examine clinical factors associated with matching quality in a mental healthcare population and we identified a number of novel findings with regards to the clinical characteristics of the matched and unmatched groups. Patients who had not had a clinical contact with SLaM in the two years prior to the linkage were significantly less likely to be matched, whereas, patients with high levels of clinical contact, indicated by frequency of face to face contact or inpatient bed days, were significantly more likely to match. Similarly, patients who had ever received a primary diagnosis within the majority F00-98 ICD-10 diagnosis codes were significantly more likely to match than patients who had never received an ICD-10 F diagnosis. Our findings provide some indication that patients with higher severity of illness and clinical service contacts proximal to the date of matching are more likely to match, possibly through these factors driving greater administrative data accuracy. Risk factors for match status may operate through different mechanisms, e.g. (i) through the type of condition and severity, (ii) as determinants of linkage error, or (iii) both, and further research is required to understand the underlying mechanisms.

Improving the quality of data linkages, by reducing missing information in the source data, is important for scientific as well as legal and ethical reasons [25]. Within the current study we found that records which did not contain an NHS number or date of birth in the source SLaM data were not



For peer review only - http://bmjopen.bmj.com/site/about/guidelines.xhtml

matched. In order to improve the missed match rate and quality of future data linkages it would be beneficial to reduce the number of missing NHS numbers and dates of birth in the source clinical data.

The evaluation of linkage quality can guide decisions about appropriate study designs [12]. However, data linkages tend to be carried out by an independent body and information about linkage processes are not always made available to researchers, known as the 'separation principle' [49]. By taking account of matching rank, in our sample we found that variation in linkage quality appeared to have very little effect on the prediction of mortality. We were able to demonstrate this as the information on missed matches and match rank was provided to us by NHS Digital. Key to health researchers being able to account for the impact of matching on the analysis of interest (in our case clinical risk factor for death), are organisations which conduct data linkages being able to provide information on match accuracy (i.e. match rates, match ranks, etc.) so that potential linkage error can be accounted for in subsequent analyses.

*Strengths and Weaknesses of the Study*

This is a large scale study of linkage error in a population of people receiving mental healthcare and one of the first studies to examine the association between patient characteristics and an optimal match. An optimal match (i.e. a match rank of one - see Table 2) indicates a higher quality match and therefore improving the number of records which achieve an optimal match is one way of improving the validity of data linkages. By identifying factors associated with an optimal match we can target specific records in the source data which can be improved (e.g. adding in missing information) in order to maximise the quality of the data linkage.

Gold standard data are rarely available but provide an easily interpretable way to measure linkage error [12], a major strength of the study is the use of gold standard death data (i.e. CRIS-derived mortality) as a reference category for examining error in the linked records. However, matching error due to false or missed matches is not the only reason that we may see discrepancies between the CRIS-derived mortality data and the linked mortality data from NHS Digital. For example, if a patient were to die abroad then the death may not be registered in UK and will therefore not always be included in the mortality data obtained from the ONS. Similarly, there are sometimes delays in death registration due to coroner investigations which can take several months. Although SLaM may be informed at the time of death, and therefore the EPR updated to reflect this, it could be several months before the official registration takes places. Despite this, it is not always possible to identify the reason for the discrepancy within the data and therefore researchers should be aware of these issues when analysing linked data.

There are some limitations of this study. The study sample was largely derived from a diverse urban and semi-urban population in South London, and is therefore not nationally representative.



For peer review only - http://bmjopen.bmj.com/site/about/guidelines.xhtml

However, the findings may be generalisable to other NHS cohorts, as the quality and type of administrative data available for matching is likely to be similar within most NHS Trusts, and national standard NHS Digital matching methodology was used for the data linkage. A further limitation is that within the unmatched group we were unable to tell apart missed matches and patients who had opted out of their data being used for secondary purposes. This is due to the strict governance requirements around opt-outs [47]. However, any researcher using data from NHS Digital, will have to account for missed matching in the same way, irrespective of whether non-matching was due to opt-out.

The data linkage in the current study was conducted by NHS Digital using deterministic methods; however, previous research has found that following probabilistic linkage methods can reduce the number of missed matches [26]. Therefore, the inclusion of probabilistic matching techniques may help to improve linkage error in future refreshes of the data linkage. Furthermore, within the current study we only examined missed matches and not false matches, and therefore we may have underestimated the effect of linkage error. Future research should aim to examine the rate of false matches in order to gain a clearer picture of the total effect of linkage error in this population.

*Conclusion*

There are significant administrative, socio-demographic, and clinical differences between the matched and unmatched records in the data linkage examined. Despite this, after adjusting for matching probability, missed matches do not appear to have had an effect on the prediction of mortality. Furthermore, after comparing models predicting gold standard CRIS-derived mortality and linked mortality we found that the models were largely the same with only slight differences in some of the odds ratios. Together these provide some indication that this effect is not driven by selection bias from matching error and provide some reassurance to studies using NHS linked data to investigate mortality.



For peer review only - http://bmjopen.bmj.com/site/about/guidelines.xhtml

**Acknowledgements**

We would like to acknowledge NHS Digital as the provider of the HES and mortality data; those who carried out the original collection and analysis of the data bear no responsibility for their further analysis or interpretation. We would also like to acknowledge the staff at the Maudsley Biomedical Research Centre (BRC) Nucleus for their continued support of the study.

**Contributors**

AJ and JD conceived the study design. Data extraction was completed by AJ. Data analysis was conducted by AJ and JD. All authors contributed to the interpretation of the data, and to the final manuscript.

**Funding**

This paper represents independent research part funded by the National Institute for Health Research (NIHR) Biomedical Research Centre (BRC) at the South London and Maudsley NHS Foundation Trust and King's College London. The work described here was part-supported by the Mental Health Data Pathfinder award to King's College London from the Medical Research Council (reference MC_PC_17214). The views expressed are those of the authors and not necessarily those of the NHS, the NIHR, the MRC, or the Department of Health. AJ, MB, RH, and RS receive salary support from the National Institute for Health Research (NIHR) Mental Health Biomedical Research Centre at South London and Maudsley NHS Foundation Trust and King's College London.

**Competing Interests**

RH has received research funding from Roche, Pfizer, Janssen, and Lundbeck. The other authors have declared that no competing interests exist.

**Ethics Approval**

CRIS was approved as a dataset for secondary analysis by the Oxford Research Ethics Committee C (18/SC/0372). Approval to conduct the data linkage was granted by the Health Research Authority (HRA) Confidentiality Advisory Group (CAG) in July 2011 (ECC 3-04(f)/2011) and by NHS Digital's Data Access Advisory Group (DAAG) in October 2016 (NIC-292279-Z2S5T). Approval to conduct the study was granted in July 2017 by the CRIS Oversight Committee (17-065).

**Data Sharing Statement**

Data are owned by a third party, Maudsley Biomedical Research Centre (BRC) Clinical Records Interactive Search (CRIS) tool, which provides access to anonymised data derived from SLaM



For peer review only

electronic medical records. These data can only be accessed by permitted individuals from within a secure firewall (i.e., the data cannot be sent elsewhere), in the same manner as the authors. For more information please contact: cris.administrator@slam.nhs.uk.

For peer review only



For peer review only - http://bmjopen.bmj.com/site/about/guidelines.xhtml

**REFERENCES**

1. Chesney, E., Goodwin, G.M. and Fazel, S. (2014) Risks of all-cause and suicide mortality in mental disorders: a meta-review. *World Psychiatry*, 13, p.p. 153-160.

2. Walker, E.R., McGee, R.E. and Druss, B.G. (2015) Mortality in Mental Disorders and Global Disease Burden Implications: A Systematic Review and Meta-analysis. *JAMA Psychiatry*, 72(4), p.p. 334-341.

3. Ilyas, A., Chesney, E. and Patel, R. (2017) Improving life expectancy in people with serious mental illness: should we place more emphasis on primary prevention. *The British Journal of Psychiatry*, 211(4), p.p. 194-197.

4. Perera, G., Broadbent, M., Callard, F., Chang, C-K., Downs, J., Dutta, R., Fernandes, A., Hayes, R.D., Henderson, M., Jackson, R., Jewell, A., Kadra, G., Little, R., Pritchard, M., Shetty, H., Tulloch, A., Stewart, R. (2016) Cohort profile of the South London and Maudsley NHS Foundation Trust Biomedical Research Centre (SLaM BRC) Case Register: current status and recent enhancement of an Electronic Mental Health Record- derived data resource. *BMJ Open*, 6:e008721.

5. Chang, C-K., Hayes, R.D., Perera, G., Broadbent, M.T.M., Fernandes, A.C., Lee, W.E., Hotopf, M. and Stewart, R. (2011) Life Expectancy at Birth for People with Serious Mental Illness and Other Major Disorders from a Secondary Mental Health Care Case Register in London. *PLoS ONE*, 6(5): e19590.

6. Fok, M.L-Y, Hayes, R.D., Chang, C-K., Stewart, R., Callard, F.J. and Moran, P. (2012) Life expectancy at birth and all-cause mortality among people with personality disorder. *Journal of Psychosomatic Research*, 73(2), p.p. 104-107.

7. Hayes, R.D., Chang, C-K., Fernandes, A., Begum, A., To, D., Broadbent, M., Hotopf, M. and Stewart, R. (2012) Associations between symptoms and all-cause mortality in individuals with serious mental illness. *Journal of Psychosomatic Research*, 72(2), p.p. 114-119.



For peer review only - http://bmjopen.bmj.com/site/about/guidelines.xhtml

8. Das-Munshi, J., Chang, C-K., Dutta, R., Morgan, C., Nazroo, J., Stewart, R. and Prince, M.J. (2017) Ethnicity and excess mortality in severe mental illness: a cohort study. *The Lancet Psychiatry*, 4, p.p. 389-399.

9. Jayatilleke, N., Hayes, R.D., Dutta, R., Shetty, H., Hotopf, M., Chang, C-K. and Stewart, R. (2017) Contributions of specific causes of death to lost life expectancy in severe mental illness. *European Psychiatry*, 43, p.p. 109-115.

10. Green, E., Ritchie, F., Mytton, J., Webber, D.J., Deave, T., Montgomery, A., Woolfrey, L., ul-Baset, K. and Chowdhury, S. (2015) Enabling Data Linkage to Maximise the Value of Public Health Research Data: full report. Project Report. Wellcome Trust, London. [Online] Available from: https://wellcome.ac.uk/sites/default/files/enabling-data-linkage-to-maximise-value-of-public-health-research-data-phrdf-mar15.pdf. [Accessed 7 March 2018].

11. Bohensky, M.A., Jolley, D., Sundararajan, V., Evans, S., Pilcher, D.V., Scott, I. and Brand, C.A. (2010) Data Linkage: A powerful research tool with potential problems. *BMC Health Services Research*, 10:346.

12. Harron, K.L., Doidge, J.C., Knight, H.E., Gilbert, R.E., Goldstein, H., Cromwell, D.A. and van der Meulen, J.H. (2017) A guide to evaluating linkage quality for the analysis of linked data. *International Journal of Epidemiology*, 0 (0), p.p. 1-12.

13. Blakely, T. and Salmond, C. (2002) Probabilistic record linkage and a method to calculate the positive predictive value. *International Journal of Epidemiology*, 31, p.p. 1246-1252.

14. Buescher, P.A. (1999) Method of Linking Medicaid Records to Birth Certificates May Affect Infant Outcome Statistics. *American Journal of Public Health,* 89(4), p.p. 564-566.

15. Bopp, M. and Minder, C.E. (2003) Mortality by education in German speaking Switzerland, 1990-1997: results from the Swizz National Cohort. *International Epidemiological Association*, 32, p.p. 346-354.

16. Zingmond, D.S., Ye, Z., Ettner, S.L. and Honghu, L. (2004) Linking hospital discharge and death records - accuracy and sources of bias. *Journal of Clinical Epidemiology*, 57, p.p. 21-29.



For peer review only - http://bmjopen.bmj.com/site/about/guidelines.xhtml

17. Jebamani, L.S., Burchill, C.A. and Martens, P.J. (2005) Using data linkage to identify First National Manitobans: technical, ethical, and political issues. *Canadian Journal of Public Health*, 96(1): S28-32.

18. Maizlish, N.A. and Herrera, L. (2005) A Record Linkage Protocol for a Diabetes Registry at Ethnically Diverse Community Health Centers. *Journal of the American Medical Informatics Association*, 12(3), p.p. 331-337.

19. Schmidlin, K., Clough-Gorr, K.M., Spoerri, A., Egger, M. and Zwahlen, M. (2013) Impact of unlinked deaths and coding changes on mortality trends in the Swiss National Cohort. *BMC Medical Informatics and Decision Making*, 13:1.

20. Kabudula, C.W., Clark, B.D., Gómez-Olive, F.X., Tollman, S., Menken, J. and Reniers, G. (2014) The promise of record linkage for assessing the uptake of health services in resource constrained settings: a pilot study from South Africa. *BMC Medical Research Methodology*, 14:71.

21. McIsaac, K.E., MacDonald, S.F., Chong, N., Moser, A., Moineddin, R., Colantonio, A., Nathens, A. and Matheson, F.I. (2016) Creating a powerful platform to explore health in a correctional population: A record linkage study. *PLOS one*, 11(8): e0161173.

22. Rosman, D.L. and Knuiman, M.W. (1994) A comparison of hospital and police road injury data. *Accident; Analysis and Prevention,* 26(2), p.p. 215-222.

23. Cryer, P.C., Westrup, S., Cook, A.C., Ashwell, V., Bridger, P. and Clarke, C. (2001) Investigation of bias after data linkage of hospital admissions data to police road traffic crash reports. *Injury Prevention,* 7, p.p. 234-241.

24. Clark, D., King, A., Sharpe, K., Connelly, G., Elliot, L., Macpherson, L.M.D., McMahon, A.D., Milligan, I., Wilson, P., Conway, D.I. and Wood, R. (2017) Linking routinely collected social work, education and health data to enable monitoring of the health and health care of school-aged children in state care ('looked after children') in Scotland: a national demonstration project. *Public Health*, 150, p.p. 101-111.



For peer review only - http://bmjopen.bmj.com/site/about/guidelines.xhtml

25. Hagger-Johnson, G., Harron, K., Fleming, T., Gilbert, R., Goldstein, H., Landy, R. and Parslow, R.C. (2015) Data linkage errors in hospital administrative data when applying a pseudonymisation algorithm to paediatric intensive care records. *BMJ Open*, 5:e008118.

26. Hagger-Johnson, G., Harron, K., Goldstein, H., Aldridge, R. and Gilbert, R. (2017) Probabilistic linking to enhance deterministic algorithms and reduce linkage errors in hospital administrative data. *Journal of Innovation in Health Informatics*, 24(2), p.p. 234-246.

27. Downs, J.M., Ford, T., Stewart, R., Epstein, S., Shetty, H., Little, R., Jewell, A., Broadbent, M., Deighton, J., Mostafa, T., Gilbert, R., Hotopf, M. and Hayes, R. (2019) An approach to linking education, social care and electronic health records for children and young people in South London: a linkage study of child and adolescent mental health service data. *BMJ Open*, 9:e024355.

28. Adams, M.M., Wilson, H.G., Casto, D.L., Berg, C.J., McDermott, J.M., Gaudino, J.A. and McCarthy, B.J. (1997) Constructing Reproductive Histories by Linking Vital Records. *American Journal of Epidemiology*, 145(4), p.p. 339-348.

29. Blakely, T., Woodward, A. and Salmond, C. (2000) Anonymous linkage of New Zealand mortality and Census data. *Australian and New Zealand Journal of Public Health,* 24(1), p.p. 92-95.

30. Gyllstrom, M.E., Jensen, J.L., Vaughan, J.N., Castellano, S.E. and Oswald, J.W. (2002) Linking Birth Certificates with Medicaid Data to Enhance Population Health Assessment: Methodological Issues Addressed. *Journal of Public Health Management Practice,* 8(4), p.p. 38-44.

31. Ringland, C., Correll, P.K., Lim, K., Williamson, M. and Marks, G.B. (2006) Hospital readmissions for asthma: a feasibility study comparing strategies for linking hospital morbidity data. *Australian and New Zealand Journal of Public Health,* 30(5), p.p. 435-439.

32. Lariscy, J.T. (2011) Differential Record Linkage by Hispanic Ethnicity and Age in Linked Mortality Studies: Implications for the Epidemiologic Paradox. *Journal of Aging and Health*, 23(8), p.p. 1263-1284.



For peer review only - http://bmjopen.bmj.com/site/about/guidelines.xhtml

33. Beckmann, K.R., Bennett, A., Young, G.P. and Roder, D.M. (2014) Treatment patterns among colorectal cancer patients in South Australia: a demonstration of the utility of population-based data linkage. *Journal of Evaluation in Clinical Practice*, 20, p.p. 467-477.

34. Moore, C.L., Gidding, H.F., Law, M.G. and Amin, J. (2016) Poor record linkage sensitivity biased outcomes in a linked cohort analysis. *Journal of Clinical Epidemiology*, 75, p.p. 70-77.

35. Baldwin, E., Johnson, K., Berthoud, H. and Dublin, S. (2015) Linking mothers and infants within electronic health records: a comparison of deterministic and probabilistic algorithms. *Pharmacoepidemiology and Drug Safety*, 24, p.p. 45-51.

36. Harron, K., Dibben, C., Boyd, J., Hjern, A., Azimaee, M., Barreto, M.L. and Goldstein, H. (2017) Challenges in administrative data linkage for research. *Big Data & Society*, 4(2), p.p. 1-12.

37. Stewart, R., Soremekun, M., Perera, G., Broadbent, M., Callard, F., Denis, M., Hotopf, M., Thornicroft, G. and Lovestone, S. (2009) The South London and Maudsley NHS Foundation Trust Biomedical Research Centre (SLAM BRC) case register: development and descriptive data. *BMC Psychiatry*, 9:51.

38. Downs, J., Gilbert, R., Hayes, R.D., Hotopf, M. and Ford, T. (2017) Linking health and education data to plan evaluate services for children. *Archives of Disease in Childhood*, 102, p.p. 599-602.

39. Chang, C-K., Hayes, R.D., Broadbent, M.T.M., Hotopf, M., Davies, E., Møller, H. and Stewart, R. (2014) A cohort study on mental disorders, stage of cancer at diagnosis and subsequent survival. *BMJ Open*, 4:e004295.

40. Woodhead, C., Ashworth, M., Broadbent, M., Callard, F., Hotopf, M., Schofield, P., Soncul, M., Stewart, R.J. and Henderson, M.J. (2016) Cardiovascular disease treatment among patients with server mental illness: a data linkage study between primary and secondary care. British *Journal of General Practice*, 66, p.p. e374-e381.

41. NHS (2019) PDS Death Notification Status Code; Description. [Online] Available from: https://www.datadictionary.nhs.uk/data_dictionary/attributes/p/paya/pds_death_notification_status_code_de.asp?shownav=1



For peer review only - http://bmjopen.bmj.com/site/about/guidelines.xhtml

42. NHS Digital (2019) Summary Care Records (SCR). [Online] Available from: https://digital.nhs.uk/services/summary-care-records-scr [Accessed 16 July 2019].

43. Academy of Medical Royal Collages. Hospital Episode Statistics (HES): improving the quality and value of hospital data. A discussion document. Leeds: Health and Social Care Information Centre, 2011. [Online] Available from: http://content.digital.nhs.uk/media/1593/Hospital-Episode-Statistics-Improving-the-quality-and-value-of-hospital-data/pdf/HES__Improving_the_quality_and_value_of__hospital_data.pdf [Accessed 9 February 2018].

44. NHS Digital (2019) Secondary Uses Service (SUS). [Online] Available from: https://digital.nhs.uk/services/secondary-uses-service-sus. [Accessed 18 January 2019].

45. NHS Digital (2019) Primary Care Mortality Database. [Online] Available from: https://digital.nhs.uk/services/primary-care-mortality-database. [Accessed 16 July 2019].

46. NHS Digital (2019) Demographics. [Online] Available from: https://digital.nhs.uk/services/demographics. [Accessed 2 April 2019].

47. NHS Digital (2018) Information on type 2 opt-outs. [Online] Available from: https://digital.nhs.uk/data-access-request-service/type-2-opt-outs. [Accessed 6 March 2018].

48. Jewell, A., Pritchard, M., Barrett, K., Green, P., Markham, S., McKenzie, S., Oliver, R., Wan, M., Downs, J. and Stewart, R. (2019) The Maudsley Biomedical Research Centre (BRC) data linkage service user and carer advisory group: creating and sustaining a successful patient and public involvement group to guide research in a complex area. *Research Engagement and Involvement*, 5:20.

49. Gilbert, R., Lafferty, R., Hagger-Johnson, G., Harron, K., Zhang, L-C, Smith, P., Dibben, C. and Goldstein, H. (2018) GUILD: GUidance for Information about Linking Data sets. *Journal of Public Health*, 40(1), p.p. 191-198.



For peer review only - http://bmjopen.bmj.com/site/about/guidelines.xhtml

**Figure 1.** Flow diagram describing how the gold standard CRIS-derived date of death is entered into the SLaM electronic medical record.

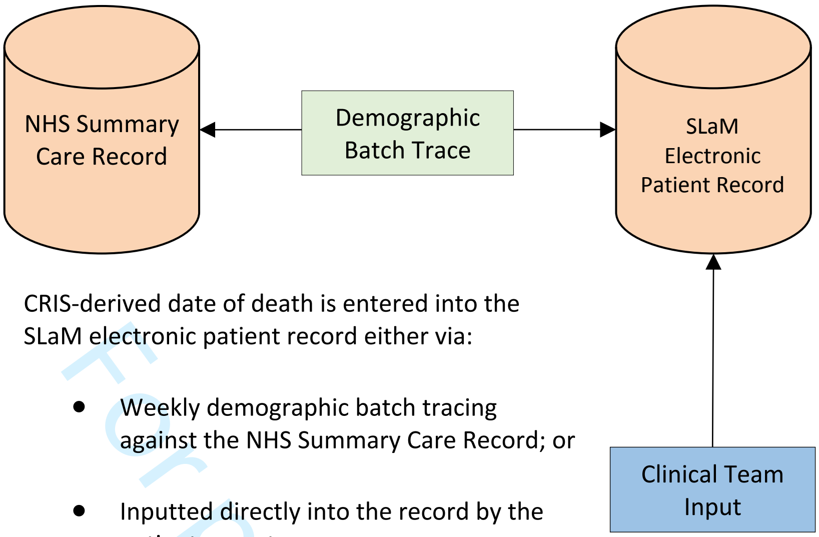


CRIS-derived date of death is entered into the SLaM electronic patient record either via:

- Weekly demographic batch tracing against the NHS Summary Care Record; or
- Inputted directly into the record by the patients care team.



For peer review only - http://bmjopen.bmj.com/site/about/guidelines.xhtml

**Figure 2.** Flow diagram of the linkage process between SLaM records and HES- ONS mortality data.

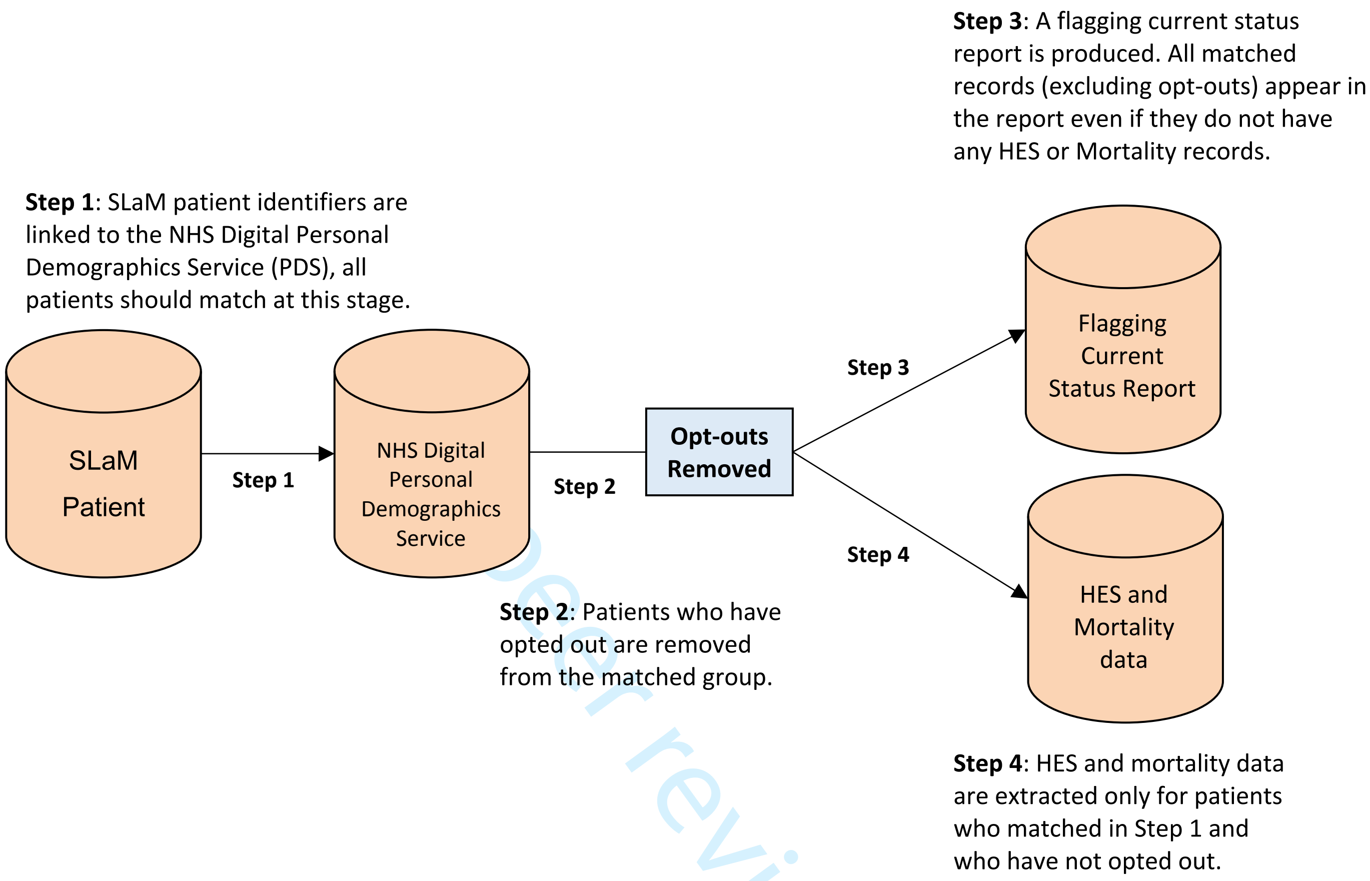




For peer review only - http://bmjopen.bmj.com/site/about/guidelines.xhtml

**Table 1.** Exposure variables.

| Exposure Variable | Description |
|---|---|
| NHS number present | Was NHS number available for linkage, binary variable (yes vs. no). |
| Date of birth present | Was date of birth available for linkage, binary variable (yes vs. no). |
| Sex present | Was sex available for linkage, binary variable (yes vs. no). |
| Postcode present | Was postcode (full or part) available for linkage, binary variable (yes vs. no). |
| Age | Age in years at time of PII extraction (20th December 2016). |
| Sex | Sex, male vs. female. |
| Patient deceased | Is the patient deceased according to the gold standard CRIS-derived mortality data (yes vs. no). |
| Ethnicity | Patient ethnicity, coded into 6 categories (i) British, Irish, or any other white ethnic groups, (ii) mixed, (iii) Indian , Pakistani, Bangladeshi, or 'other Asian', (iv) Caribbean, African, or 'other black', (v) other, and (vi) not stated. |
| Resident in local catchment area | Was the patient resident in a SLaM catchment area, i.e. Lambeth, Lewisham, Southwark, or Croydon, at the time of PII extraction, binary variable (yes vs. no). |
| Quartiles of neighbourhood deprivation | Quartiles of deprivation score: 1st (most deprived), 2nd, 3rd, 4th (least deprived). |
| Referral Status in past two years | Referral status in the two years prior to PII extraction; (i) accepted, (ii) discharged, (iii) rejected, or (iv) no referral in 2 years prior to linkage. |

For peer review only



For peer review only - http://bmjopen.bmj.com/site/about/guidelines.xhtml

| | |
|---|---|
| Primary Diagnosis Ever | A primary diagnosis ever of F00-F09: organic, including symptomatic, mental disorders, F10-F19: mental and behavioural disorders due to psychoactive substance use, F20-F29: schizophrenia, schizotypal and delusional disorders, F30-F39: mood (affective) disorders, F40-F49: neurotic, stress-related and somatoform disorders, F50-F59: behavioural syndromes associated with physiological disturbances and physical factors, F60-F69: disorders of adult personality and behaviour, F70-F79: mental retardation, F80-89: disorders of psychological development, F90-F98: behavioural and emotional disorders with onset usually occurring in childhood and adolescence, or Other diagnosis. Binary variables (yes vs. no). Other diagnosis category includes all non-mental health (i.e. non F code) ICD-10 diagnoses as well as 'F99: Mental disorder, not otherwise specified' and 'F00-F99: Mental and behavioural disorders'. Patients can have multiple primary diagnoses throughout their time at SLaM and therefore can appear in multiple groups. |
| Face to face contact | Quartiles of face to face contact with SLaM from 1st January 2006 to 20th December 2016; 1st (least face to face contact), 2nd, 3rd, 4th (most face to face contact). |
| Inpatient bed days | Number of SLaM inpatient bed days from 1st January 2006 to 20th December 2016, coded into; (i) none (0), (ii) low (1-2 days), (iii) moderate (3-31 days), and (iv) high (32+). |
| Optimal match | Binary variable (optimal match vs. non-optimal match). For those records which were successfully matched by NHS Digital only. An optimal match represents a 'perfect' match or a match rank of one (see Table 2), i.e. the records matched on all supplied patient identifiers (NHS number, sex, date of birth, and postcode). |



For peer review only - http://bmjopen.bmj.com/site/about/guidelines.xhtml

**Table 2.** NHS Digital deterministic matching steps.

| Match Rank | NHS Number | Date of Birth | Sex | Postcode[a] | |
|---|---|---|---|---|---|
| 1 | Exact | Exact | Exact | Exact | |
| 2 | Exact | Exact | Exact | | |
| 3 | Exact | Partial | Exact | Exact | |
| 4 | Exact | Partial | Exact | | |
| 5 | Exact | | | Exact | |
| 6 | | Exact | Exact | Exact | where NHS number does not contradict the match and DOB is not 1 January and the Postcode is in the 'ignore' list |
| 7 | | Exact | Exact | Exact | where NHS number does not contradict the match and DOB is not 1 January |
| 8 | Exact | | | | |

**Note.** [a] Current or most recently recorded postcode.

For peer review only



For peer review only - http://bmjopen.bmj.com/site/about/guidelines.xhtml

**Table 3.** Logistic regression analysis examining administrative, socio-demographic, and clinical associations with match status (match vs. missed match).

| Variable | Total Population (N=265,300) | Matched (*n*=248,698 93.74%) | Missed Match (*n*=16,602 6.26%) | OR (95% CI) | aOR[1] (95% CI) |
|---|---|---|---|---|---|
| **Administrative variables** | | | | | |
| NHS number present: *n* (%) | 259545 (97.83) | 248698 (100.00) | 10847 (65.34) | | |
| Date of birth present: *n* (%) | 265010 (99.89) | 248698 (100.00) | 16312 (98.25) | | |
| Sex present: *n* (%) | 265246 (99.98) | 248663 (99.99) | 16583 (99.89) | **8.14 (4.66-14.23)**** | |
| Postcode present: *n* (%) | 260466 (98.18) | 245278 (98.62) | 15188 (91.48) | **6.68 (6.26-7.12)**** | |
| **Sociodemographic variables** | | | | | |
| Age: mean (SD) | 43.40 (22.69) | 43.50 (22.79) | 41.92 (21.09) | **1.003 (1.002-1.004)**** | 0.999 (0.998-1.000) |
| Male Sex: *n* (%) | 132730 (50.04) | 123818 (49.79) | 8912 (53.74) | **0.85 (0.83-0.88)**** | **0.93 (0.89-0.96)**** |
| Patient deceased: *n* (%) | 30173 (11.37) | 29057 (11.68) | 1116 (6.72) | **1.84 (1.73-1.95)**** | **1.41 (1.30-1.53)**** |
| Ethnicity: *n* (%) | | | | | |
| British, Irish, or any other white ethnic groups | 138495 (58.54) | 131150 (58.93) | 7345 (52.35) | (reference) | (reference) |
| Mixed | 6853 (2.90) | 6477 (2.91) | 376 (2.68) | 0.96 (0.87-1.07) | **0.82 (0.73-0.91)**** |
| Indian, Pakistani, Bangladeshi, or ‘other Asian’ | 10889 (4.60) | 10155 (4.56) | 734 (5.23) | **0.77 (0.72-0.84)**** | **0.78 (0.71-0.85)**** |
| Caribbean, African, or ‘other black’ | 40725 (17.21) | 38013 (17.08) | 2712 (19.33) | **0.78 (0.75-0.82)**** | **0.70 (0.66-0.74)**** |
| Other | 16650 (7.04) | 15393 (6.92) | 1257 (8.96) | **0.69 (0.64-0.73)**** | **0.77 (0.72-0.83)**** |
| Not stated | 22961 (9.71) | 21354 (9.60) | 1607 (11.45) | **0.74 (0.70-0.79)**** | **0.89 (0.83-0.95)*** |
| Resident in local catchment area: *n* (%) | 187773 (73.42) | 177766 (73.53) | 10007 (71.47) | **1.11 (1.07-1.15)**** | 0.97 (0.93-1.02) |
| Quartiles of neighbourhood deprivation: *n* (%) | | | | | |
| 1st (most deprived) | 63476 (25.03) | 60220 (25.09) | 3256 (24.00) | (reference) | (reference) |
| 2nd | 63452 (25.02) | 59786 (24.91) | 3666 (27.02) | **0.88 (0.84-0.93)**** | **0.90 (0.86-0.95)**** |
| 3rd | 63449 (25.02) | 60052 (25.02) | 3397 (25.04) | 0.96 (0.91-1.00) | 1.01 (0.95-1.07) |
| 4th (least deprived) | 63221 (24.93) | 59973 (24.99) | 3248 (23.94) | 1.00 (0.95-1.05) | 1.06 (1.00-1.12) |
| **Clinical variables** | | | | | |
| Referral status in 2 years prior to linkage: *n* (%) | | | | | |
| Accepted | 12041 (4.56) | 11423 (4.61) | 618 (3.78) | (reference) | (reference) |
| Discharged | 45228 (17.11) | 42394 (17.10) | 2834 (17.33) | **0.81 (0.74-0.89)**** | **0.87 (0.78-0.97)*** |
| Rejected | 9628 (3.64) | 8921 (3.60) | 707 (4.32) | **0.68 (0.61-0.76)**** | 0.88 (0.76-1.01) |



For peer review only - http://bmjopen.bmj.com/site/about/guidelines.xhtml

| | | | | | |
|---|---|---|---|---|---|
| No referral in 2 years prior to linkage | 197414 (74.69) | 185221 (74.70) | 12193 (74.57) | **0.82 (0.76-0.89)*** | **0.87 (0.79-0.95)*** |
| Primary diagnosis ever: *n (%)* | | | | | |
| F00-F09: organic, including symptomatic, mental disorders | 25249 (9.52) | 24248 (9.75) | 1001 (6.03) | **1.68 (1.58-1.80)**** | **1.37 (1.26-1.49)**** |
| F10-F19: mental and behavioural disorders due to psychoactive substance use | 27755 (10.46) | 26158 (10.52) | 1597 (9.62) | **1.10 (1.05-1.16)**** | **1.12 (1.05-1.21)*** |
| F20-F29:schizophrenia, schizotypal and delusional disorders | 18516 (6.98) | 17323 (6.97) | 1193 (7.19) | 0.97 (0.91-1.03) | 1.02 (0.93-1.11) |
| F30-F39: mood (affective) disorders | 42578 (16.05) | 40515 (16.29) | 2063 (12.43) | **1.37 (1.31-1.44)**** | **1.28 (1.21-1.36)**** |
| F40-F49: neurotic, stress-related and somatoform disorders | 33248 (12.53) | 31599 (12.71) | 1649 (9.93) | **1.32 (1.25-1.39)**** | **1.27 (1.19-1.35)**** |
| F50-F59: behavioural syndromes associated with physiological disturbances and physical factors | 8743 (3.30) | 8388 (3.37) | 355 (2.14) | **1.60 (1.43-1.78)**** | **1.49 (1.31-1.69)**** |
| F60-F69: disorders of adult personality and behaviour | 6945 (2.62) | 6585 (2.65) | 360 (2.17) | **1.23 (1.10-1.37)**** | 0.95 (0.84-1.07) |
| F70-F79: mental retardation | 3018 (1.14) | 2898 (1.17) | 120 (0.72) | **1.62 (1.35-1.95)**** | **1.55 (1.26-1.89)**** |
| F80-F89: disorders of psychological development | 7242 (2.73) | 6991 (2.81) | 251 (1.51) | **1.88 (1.66-2.14)**** | **1.86 (1.61-2.15)**** |
| F90-F98: behavioural and emotional disorders with onset usually occurring in childhood and adolescence | 17011 (6.41) | 16363 (6.58) | 648 (3.90) | **1.74 (1.60-1.88)**** | **1.58 (1.45-1.74)**** |
| Other diagnosis | 119638 (45.10) | 112556 (45.26) | 7082 (42.66) | **1.11 (1.08-1.15)**** | **1.23 (1.18-1.28)**** |
| Quartiles of face to face contacts: *n* (%) | | | | | |
| 1st (least face to face contact) | 76602 (28.87) | 70822 (28.48) | 5780 (34.82) | (reference) | (reference) |
| 2nd | 64914 (24.47) | 59811 (24.02) | 5103 (30.74) | **0.96 (0.92-0.99)*** | **0.94 (0.89-0.99)*** |
| 3rd | 59337 (22.37) | 56116 (22.56) | 3221 (19.40) | **1.42 (1.36-1.49)**** | **1.15 (1.08-1.22)**** |
| 4th (most face to face contact) | 64447 (24.29) | 61949 (24.91) | 2498 (15.05) | **2.02 (1.93-2.12)**** | **1.42 (1.33-1.52)**** |
| Inpatient bed days: *n* (%) | | | | | |
| None (0) | 238573 (89.93) | 223527 (89.88) | 15046 (90.63) | (reference) | (reference) |
| Low (1-2 days) | 1446 (0.55) | 1342 (0.54) | 104 (0.63) | 0.87 (0.71-1.06) | 0.86 (0.67-1.09) |
| Moderate (3-31 days) | 9825 (3.70) | 9133 (3.67) | 692 (4.17) | **0.89 (0.82-0.96)*** | **0.90 (0.81-1.00)*** |
| High (32+ days) | 15456 (5.83) | 14696 (5.91) | 760 (4.58) | **1.30 (1.21-1.40)**** | **1.14 (1.03-1.26)*** |

**Note.** *$p$<0.05. **$p$<0.001. [1]adjusted for all other variables listed in the table. Missing data: age (*n*=275); sex (*n*=55); ethnicity (*n*=28,727); resident in local catchment area (*n*=9,537); quartiles of neighbourhood deprivation (*n*=11,702); referral status (*n*=989).



For peer review only - http://bmjopen.bmj.com/site/about/guidelines.xhtml

**Table 4.** Logistic regression analysis examining socio-demographic and clinical associations with gold standard CRIS-derived mortality.

| Variable | Total Population (N=265,300) | Patient Deceased (*n*=30,173 11.37%) | Patient Alive (*n*=235,127 88.63%) | OR (95% CI) | aOR[1] (95% CI) |
|---|---|---|---|---|---|
| **Sociodemographic variables** | | | | | |
| Age: mean (SD) | 43.40 (22.69) | 77.32 (19.06) | 39.05 (19.17) | **1.090 (1.089-1.091)**** | **1.092 (1.090-1.093)**** |
| Male Sex: *n* (%) | 132730 (50.04) | 14848 (49.21) | 117882 (50.15) | **0.96 (0.94-0.99)*** | **1.48 (1.43-1.53)**** |
| Ethnicity coded: *n* (%) | | | | | |
| British, Irish, or any other white ethnic groups | 138495 (58.54) | 21993 (78.19) | 116502 (55.89) | (reference) | (reference) |
| Mixed | 6853 (2.90) | 208 (0.74) | 6645 (3.19) | **0.17 (0.14-0.19)**** | **0.73 (0.61-0.86)**** |
| Indian, Pakistani, Bangladeshi, or 'other Asian' | 10889 (4.60) | 1006 (3.58) | 9883 (4.74) | **0.54 (0.50-0.58)**** | **0.70 (0.65-0.76)**** |
| Caribbean, African, or any 'other black' | 40725 (17.21) | 2914 (10.36) | 37811 (18.14) | **0.41 (0.39-0.43)**** | **0.63 (0.60-0.66)**** |
| Other | 16650 (7.04) | 705 (2.51) | 15945 (7.65) | **0.23 (0.22-0.25)**** | **0.52 (0.48-0.57)**** |
| Not stated | 22961 (9.71) | 1301 (4.63) | 705 (2.51) | **0.32 (0.30-0.34)**** | **0.91 (0.84-0.98)*** |
| Resident in local catchment area: *n* (%) | 187773 (73.42) | 23870 (81.41) | 163903 (72.38) | **1.67 (1.62-1.72)**** | **0.84 (0.80-0.88)**** |
| Quartiles of neighbourhood deprivation: *n* (%) | | | | | |
| 1st (most deprived) | 63476 (25.03 | 7626 (26.11) | 55850 (24.89) | (reference) | (reference) |
| 2nd | 63452 (25.02) | 7049 (24.13) | 56403 (25.14) | **0.92 (0.88-0.95)**** | **1.08 (1.03-1.13)*** |
| 3rd | 63449 (25.02) | 7375 (25.25) | 56074 (24.99) | **0.96 (0.93-1.00)*** | **1.25 (1.19-1.31)**** |
| 4th (least deprived) | 63221 (24.93) | 7161 (24.51) | 56060 (24.98) | **0.94 (0.90-0.97)**** | **1.31 (1.25-1.37)**** |
| **Clinical variables** | | | | | |
| Primary diagnosis ever: *n (%)* | | | | | |
| F00-F09: organic, including symptomatic, mental disorders | 25249 (9.52) | 12641 (41.90) | 12608 (5.36) | **12.73 (12.36-13.10)**** | **1.10 (1.05-1.15)**** |
| F10-F19: mental and behavioural disorders due to psychoactive substance use | 27755 (10.46) | 2823 (9.36) | 24932 (10.60) | **0.87 (0.84-0.91)**** | **1.39 (1.31-1.48)**** |
| F20-F29:schizophrenia, schizotypal and delusional disorders | 18516 (6.98) | 2300 (7.62) | 16216 (6.90) | **1.11 (1.06-1.17)**** | **1.08 (1.01-1.15)*** |
| F30-F39: mood (affective) disorders | 42578 (16.05) | 5027 (16.66) | 37551 (15.97) | **1.05 (1.02-1.09)*** | **0.93 (0.88-0.97)*** |



For peer review only - http://bmjopen.bmj.com/site/about/guidelines.xhtml

| | | | | | |
|---|---|---|---|---|---|
| F40-F49: neurotic, stress-related and somatoform disorders | 33248 (12.53) | 2331 (7.73) | 30917 (13.15) | **0.55 (0.53-0.58)**** | **0.73 (0.69-0.78)**** |
| F50-F59: behavioural syndromes associated with physiological disturbances and physical factors | 8743 (3.30) | 148 (0.49) | 8595 (3.66) | **0.13 (0.11-0.15)**** | **0.47 (0.39-0.57)**** |
| F60-F69: disorders of adult personality and behaviour | 6945 (2.62) | 415 (1.38) | 6530 (2.78) | **0.49 (0.44-0.54)**** | 1.02 (0.91-1.15) |
| F70-F79: mental retardation | 3018 (1.14) | 311 (1.03) | 2707 (1.15) | 0.89 (0.79-1.01) | **1.24 (1.08-1.44)*** |
| F80-F89: disorders of psychological development | 7242 (2.73) | 54 (0.18) | 7188 (3.06) | **0.06 (0.04-0.07)**** | **0.65 (0.49-0.87)*** |
| F90-F98: behavioural and emotional disorders with onset usually occurring in childhood and adolescence | 17011 (6.41) | 77 (0.26) | 16934 (7.20) | **0.03 (0.03-0.04)**** | **0.45 (0.35-0.58)**** |
| Other diagnosis | 119638 (45.10) | 11014 (36.50) | 108624 (46.20) | **0.67 (0.65-0.69)**** | **0.65 (0.63-0.67)**** |
| Quartiles of face to face contacts: *n* (%) | | | | | |
| 1st (least face to face contact) | 76602 (28.87) | 5926 (19.64) | 70676 (30.06) | (reference) | (reference) |
| 2nd | 64914 (24.47) | 7767 (25.74) | 57147 (24.30) | **1.62 (1.56-1.68)**** | **1.10 (1.04-1.16)**** |
| 3rd | 59337 (22.37) | 8798 (29.16) | 50539 (21.49) | **2.08 (2.00-2.15)**** | **1.11 (1.05-1.17)**** |
| 4th (most face to face contact) | 64447 (24.29) | 7682 (25.46) | 56765 (24.14) | **1.61 (1.56-1.67)**** | 1.00 (0.95-1.06) |
| Inpatient bed days: *n* (%) | | | | | |
| None (0) | 238573 (89.93) | 26688 (88.45) | 211885 (90.12) | (reference) | (reference) |
| Low (1-2 days) | 1446 (0.55) | 102 (0.34) | 1344 (0.57) | **0.60 (0.49-0.74)**** | **1.52 (1.20-1.93)**** |
| Moderate (3-31 days) | 9825 (3.70) | 1034 (3.43) | 8791 (3.74) | **0.93 (0.87-1.00)*** | **1.58 (1.45-1.71)**** |
| High (32+ days) | 15456 (5.83) | 2349 (7.79) | 13107 (5.57) | **1.42 (1.36-1.49)**** | **1.59 (1.49-1.70)**** |

**Note.** *$p$<0.05. **$p$<0.001. [1]adjusted for all other variables listed in the table. Missing data: age (*n*=275); sex (*n*=55); ethnicity (*n*=28,727); resident in local catchment area (*n*=9,537); quartiles of neighbourhood deprivation (*n*=11,702); referral status (*n*=989).



For peer review only - http://bmjopen.bmj.com/site/about/guidelines.xhtml

**Table 5.** Logistic regression analysis examining socio-demographic and clinical associations with linked HES-ONS mortality in the matched cohort.

| Variable | Total Population (N=248,698) | Patient Deceased (*n*=28,161 11.32%) | Patient Alive (*n*=220,537 88.68%) | OR (95% CI) | aOR[1] (95% CI) | aOR[2] (95% CI) |
|---|---|---|---|---|---|---|
| **Sociodemographic variables** | | | | | | |
| Age: mean (SD) | 43.50 (22.79) | 77.75 (18.75) | 39.13 (19.28) | **1.091 (1.090-1.092)**** | **1.093 (1.092-1.095)**** | **1.092 (1.091-1.094)**** |
| Male Sex: *n* (%) | 123818 (49.79) | 13824 (49.09) | 109994 (49.88) | **0.97 (0.95-0.99)*** | **1.51 (1.45-1.56)**** | **1.50 (1.45-1.55)**** |
| Ethnicity coded: *n* (%) | | | | | | |
| British, Irish, or any other white ethnic groups | 131150 (58.93) | 20717 (78.96) | 110433 (56.26) | (reference) | (reference) | (reference) |
| Mixed | 6477 (2.91) | 180 (0.69) | 6297 (3.21) | **0.15 (0.13-0.18)**** | **0.67 (0.56-0.80)**** | **0.65 (0.54-0.79)**** |
| Indian, Pakistani, Bangladeshi, or ‘other Asian’ | 10155 (4.56) | 915 (3.49) | 9240 (4.71) | **0.53 (0.49-0.57)**** | **0.68 (0.62-0.74)**** | **0.67 (0.62-0.73)**** |
| Caribbean, African, or any ‘other black’ | 38013 (17.08) | 2611 (9.95) | 35402 (18.03) | **0.39 (0.38-0.41)**** | **0.60 (0.57-0.64)**** | **0.60 (0.57-0.63)**** |
| Other | 15393 (6.92) | 626 (2.39) | 14767 (7.52) | **0.23 (0.21-0.25)**** | **0.50 (0.45-0.55)**** | **0.50 (0.46-0.55)**** |
| Not stated | 21354 (9.60) | 1189 (4.53) | 20165 (10.27) | **0.31 (0.30-0.33)**** | 0.94 (0.87-1.02) | **0.92 (0.86-1.00)*** |
| Resident in local catchment area: *n* (%) | 177766 (73.53) | 22329 (81.48) | 155437 (72.51) | **1.67 (1.62-1.72)**** | **0.84 (0.80-0.88)**** | **0.84 (0.80-0.88)**** |
| Quartiles of neighbourhood deprivation: *n* (%) | | | | | | |
| 1st (most deprived) | 60220 (25.09) | 7217 (26.42) | 53003 (24.92) | (reference) | (reference) | (reference) |
| 2nd | 59786 (24.91) | 6573 (24.06) | 53213 (25.02) | **0.91 (0.88-0.94)**** | **1.08 (1.03-1.13)*** | **1.08 (1.03-1.13)*** |
| 3rd | 60052 (25.02) | 6857 (25.10) | 53195 (25.01) | **0.95 (0.91-0.98)*** | **1.23 (1.17-1.30)**** | **1.23 (1.17-1.29)**** |
| 4th (least deprived) | 59973 (24.99) | 6667 (24.41) | 53306 (25.06) | **0.92 (0.89-0.95)**** | **1.30 (1.23-1.36)**** | **1.29 (1.23-1.36)**** |
| **Clinical variables** | | | | | | |
| Primary diagnosis ever[a]: *n* (%) | | | | | | |
| F00-F09: organic, including symptomatic, mental disorders | 24248 (9.75) | 11940 (42.40) | 12308 (5.58) | **12.45 (12.09-12.83)**** | **1.05 (1.00-1.11)*** | **1.06 (1.01-1.11)*** |
| F10-F19: mental and behavioural disorders due to psychoactive substance use | 26158 (10.52) | 2549 (9.05) | 23609 (10.71) | **0.83 (0.80-0.87)**** | **1.35 (1.27-1.44)**** | **1.36 (1.28-1.45)**** |



For peer review only - http://bmjopen.bmj.com/site/about/guidelines.xhtml

| | | | | | | |
|---|---|---|---|---|---|---|
| F20-F29:schizophrenia, schizotypal and delusional disorders | 17323 (6.97) | 2081 (7.39) | 15242 (6.91) | **1.07 (1.02-1.13)*** | 1.06 (0.99-1.14) | 1.05 (0.98-1.13) |
| F30-F39: mood (affective) disorders | 40515 (16.29) | 4715 (16.74) | 35800 (16.23) | **1.04 (1.00-1.07)*** | **0.92 (0.88-0.97)**** | **0.92 (0.88-0.97)*** |
| F40-F49: neurotic, stress-related and somatoform disorders | 31599 (12.71) | 2187 (7.77) | 29412 (13.34) | **0.55 (0.52-0.57)**** | **0.73 (0.68-0.77)**** | **0.72 (0.68-0.77)**** |
| F50-F59: behavioural syndromes associated with physiological disturbances and physical factors | 8388 (3.37) | 136 (0.48) | 8252 (3.74) | **0.12 (0.11-0.15)**** | **0.47 (0.38-0.57)**** | **0.48 (0.39-0.58)**** |
| F60-F69: disorders of adult personality and behaviour | 6585 (2.65) | 368 (1.31) | 6217 (2.82) | **0.46 (0.41-0.51)**** | 0.99 (0.88-1.13) | 0.98 (0.86-1.12) |
| F70-F79: mental retardation | 2898 (1.17) | 282 (1.00) | 2616 (1.19) | **0.84 (0.74-0.95)*** | **1.17 (1.01-1.37)*** | **1.17 (1.01-1.37)*** |
| F80-F89: disorders of psychological development | 6991 (2.81) | 47 (0.17) | 6944 (3.15) | **0.05 (0.04-0.07)**** | **0.60 (0.44-0.82)*** | **0.61 (0.45-0.83)*** |
| F90-F98: behavioural and emotional disorders with onset usually occurring in childhood and adolescence | 16363 (6.58) | 67 (0.24) | 16296 (7.39) | **0.03 (0.02-0.04)**** | **0.42 (0.32-0.55)**** | **0.43 (0.33-0.56)**** |
| Other diagnosis | 112556 (45.26) | 10275 (36.49) | 102281 (46.38) | **0.66 (0.65-0.68)**** | **0.64 (0.62-0.66)**** | **0.64 (0.62-0.66)**** |
| Quartiles of face to face contacts: *n* (%) | | | | | | |
| 1st (least face to face contact) | 70822 (28.48) | 5496 (19.52) | 65326 (29.62) | (reference) | (reference) | (reference) |
| 2nd | 59811 (24.05) | 7263 (25.79) | 52548 (23.83) | **1.64 (1.58-1.70)**** | **1.09 (1.03-1.15)*** | **1.09 (1.04-1.15)*** |
| 3rd | 56116 (22.56) | 8282 (29.41) | 47834 (21.69) | **2.06 (1.99-2.13)**** | **1.11 (1.05-1.17)**** | **1.11 (1.05-1.18)**** |
| 4th (most face to face contact) | 61949 (24.91) | 7120 (25.28) | 54829 (24.86) | **1.54 (1.49-1.60)**** | 0.99 (0.93-1.05) | 1.00 (0.94-1.06) |
| Inpatient bed days: *n* (%) | | | | | | |
| None (0) | 223527 (89.88) | 24994 (88.75) | 198533 (90.02) | (reference) | (reference) | (reference) |
| Low (1-2 days) | 1342 (0.54) | 86 (0.31) | 1256 (0.57) | **0.54 (0.44-0.68)**** | **1.40 (1.09-1.81)*** | **1.36 (1.04-1.78)*** |
| Moderate (3-31 days) | 9133 (3.67) | 939 (3.33) | 8194 (3.72) | **0.91 (0.85-0.98)*** | **1.54 (1.41-1.68)**** | **1.53 (1.40-1.67)**** |
| High (32+ days) | 14696 (5.91) | 2142 (7.61) | 12554 (5.69) | **1.36 (1.29-1.42)**** | **1.55 (1.45-1.66)**** | **1.55 (1.44-1.66)**** |
| Optimal Match | 203552 (84.15) | 178731 (87.81) | 24821 (12.19) | **0.63 (0.61-0.66)**** | ----- | **0.85 (0.81-0.90)**** |

**Note.** *$p<0.05$. **$p<0.001$. [1]adjusted for all other variables listed in the table (except optimal match). [2]adjusted for optimal match rank and inverse probability weighting for matching. Missing data: sex (*n*=37); ethnicity (*n*=26,156); resident in local catchment area (*n*=6,937); quartiles of neighbourhood deprivation (*n*=8,667); optimal match (*n*=6,808).



For peer review only - http://bmjopen.bmj.com/site/about/guidelines.xhtml

## SUPPLEMENTARY MATERIAL

**Table 1.** Logistic regression analysis examining administrative, socio-demographic, and clinical associations with an optimal match.

| Variable | Total Population (N=241,890) | Optimal Match (*n*=203,552 84.15%) | Non-optimal Match (*n*=38,338 15.85%) | OR (95% CI) | aOR[1] (95% CI) |
|---|---|---|---|---|---|
| **Sociodemographic variables** | | | | | |
| Age: mean (SD) | 43.70 (22.89) | 44.68 (23.01) | 38.47 (21.52) | **0.987 (0.987-0.988)**** | **0.985 (0.984-0.986)**** |
| Male Sex: *n* (%) | 119634 (49.46) | 99187 (48.73) | 20447 (53.38) | **1.20 (1.18-1.23)**** | **1.14 (1.12-1.17)**** |
| Patient deceased: *n* (%) | 28790 (11.90) | 25629 (12.59) | 3161 (8.25) | **0.63 (0.61-0.65)**** | 0.95 (0.91-1.00) |
| Ethnicity coded: *n* (%) | | | | | |
| British, Irish, or any other white ethnic groups | 128547 (59.32) | 109139 (59.71) | 19408 (57.25) | (reference) | (reference) |
| Mixed | 6306 (2.91) | 5168 (2.83) | 1138 (3.36) | **1.24 (1.16-1.32)**** | 1.00 (0.93-1.07) |
| Indian, Pakistani, Bangladeshi, or 'other Asian' | 9745 (4.50) | 8348 (4.57) | 1397 (4.12) | **0.94 (0.89-0.99)*** | **0.90 (0.85-0.96)*** |
| Caribbean, African, or any 'other black' | 37052 (17.10) | 31115 (17.02) | 5937 (17.51) | **1.07 (1.04-1.11)**** | 0.97 (0.93-1.00) |
| Other | 14728 (6.80) | 12248 (6.70) | 2480 (7.32) | **1.14 (1.09-1.19)**** | **1.08 (1.03-1.13)*** |
| Not stated | 20319 (9.38) | 16778 (9.18) | 3541 (10.45) | **1.19 (1.14-1.23)**** | **1.04 (1.00-1.09)*** |
| Resident in local catchment area: *n* (%) | 172567 (73.34) | 145594 (73.34) | 26973 (73.37) | 1.00 (0.98-1.03) | **1.13 (1.09-1.16)**** |
| Quartiles of neighbourhood deprivation: *n* (%) | | | | | |
| 1st (most deprived) | 58789 (25.15) | 49830 (25.24) | 8959 (24.67) | (reference) | (reference) |
| 2nd | 58146 (24.87) | 48785 (24.71) | 9361 (25.78) | **1.07 (1.03-1.10)**** | 1.01 (0.98-1.05) |
| 3rd | 58342 (24.96) | 49200 (24.92) | 9142 (25.18) | **1.03 (1.00-1.07)*** | 0.97 (0.94-1.01) |
| 4th (least deprived) | 58488 (25.02) | 49638 (25.14) | 8850 (24.37) | 0.99 (0.96-1.02) | **0.92 (0.89-0.96)**** |
| **Clinical variables** | | | | | |
| Referral status in past 2 years: *n* (%) | | | | | |
| Accepted | 11061 (4.59) | 9445 (4.65) | 1616 (4.23) | (reference) | (reference) |
| Discharged | 41227 (17.09) | 35007 (17.25) | 6220 (16.27) | 1.04 (0.98-1.10) | **1.11 (1.04-1.18)*** |
| Rejected | 8559 (3.55) | 7060 (3.48) | 1499 (3.92) | **1.24 (1.06-1.18)**** | **1.10 (1.01-1.21)*** |
| No referral in 2 year prior to linkage | 180330 (74.77) | 151442 (74.62) | 28888 (75.58) | **1.11 (1.06-1.18)**** | **1.24 (1.17-1.32)**** |
| Primary diagnosis ever: *n (%)* | | | | | |
| F00-F09: organic, including symptomatic, mental disorders | 24095 (9.96) | 21191 (10.41) | 2904 (7.57) | **0.71 (0.68-0.73)**** | **1.31 (1.24-1.39)**** |
| F10-F19: mental and behavioural disorders due to psychoactive substance use | 25530 (10.55) | 21124 (10.38) | 4406 (11.49) | **1.12 (1.08-1.16)**** | **1.17 (1.12-1.22)**** |

For peer review only

| | | | | | |
|---|---|---|---|---|---|
| F20-F29:schizophrenia, schizotypal and delusional disorders | 16889 (6.98) | 14328 (7.04) | 2561 (6.68) | **0.95 (0.91-0.99)*** | 1.05 (0.99-1.11) |
| F30-F39: mood (affective) disorders | 39799 (16.45) | 34512 (16.95) | 5287 (13.79) | **0.78 (0.76-0.81)**** | **0.88 (0.85-0.92)**** |
| F40-F49: neurotic, stress-related and somatoform disorders | 30943 (12.79) | 26729 (13.13) | 4214 (10.99) | **0.82 (0.79-0.85)**** | **0.83 (0.79-0.86)**** |
| F50-F59: behavioural syndromes associated with physiological disturbances and physical factors | 8086 (3.34) | 6634 (3.26) | 1452 (3.79) | **1.17 (1.10-1.24)**** | **1.12 (1.05-1.19)*** |
| F60-F69: disorders of adult personality and behaviour | 6509 (2.69) | 5521 (2.71) | 988 (2.58) | 0.95 (0.89-1.02) | 0.98 (0.91-1.05) |
| F70-F79: mental retardation | 2810 (1.16) | 2448 (1.20) | 362 (0.94) | **0.78 (0.70-0.87)**** | **0.82 (0.73-0.92)**** |
| F80-F89: disorders of psychological development | 6804 (2.81) | 5673 (2.79) | 1131 (2.95) | 1.06 (0.99-1.13) | **0.76 (0.70-0.81)**** |
| F90-F98: behavioural and emotional disorders with onset usually occurring in childhood and adolescence | 15931 (6.59) | 12877 (6.33) | 3054 (7.97) | **1.28 (1.23-1.34)**** | 0.96 (0.91-1.00) |
| Other diagnosis | 109854 (45.41) | 92623 (45.50) | 17231 (44.94) | **0.98 (0.96-1.00)*** | **0.96 (0.94-0.99)**** |
| Quartiles of face to face contacts: *n* (%) | | | | | |
| 1st (least face to face contact) | 68150 (28.17) | 56507 (27.76) | 11643 (30.37) | (reference) | (reference) |
| 2nd | 58128 (24.03) | 48702 (23.93) | 9426 (24.59) | **0.94 (0.91-0.97)**** | 1.01 (0.97-1.04) |
| 3rd | 54781 (22.65) | 46311 (22.75) | 8470 (22.09) | **0.89 (0.86-0.92)**** | **0.95 (0.91-0.99)*** |
| 4th (most face to face contact) | 60831 (25.15) | 52032 (25.56) | 8799 (22.95) | **0.82 (0.80-0.85)**** | **0.89 (0.85-0.93)**** |
| Inpatient bed days: *n* (%) | | | | | |
| None (0) | 216890 (89.66) | 182362 (89.59) | 34528 (90.06) | (reference) | (reference) |
| Low (1-2 days) | 1327 (0.55) | 1106 (0.54) | 221 (0.58) | 1.06 (0.91-1.22) | 1.06 (0.91-1.24) |
| Moderate (3-31 days) | 9064 (3.75) | 7607 (3.74) | 1457 (3.80) | 1.01 (0.96-1.07) | 1.05 (0.98-1.12) |
| High (32+ days) | 14609 (6.04) | 12477 (6.13) | 2132 (5.56) | **0.90 (0.86-0.95)**** | 1.00 (0.95-1.06) |

**Note.** Optimal match data missing for *n*=6,808 (2.75%) matched records. *****$p$<0.05. ******$p$<0.001. [1]adjusted for all other variables listed in the table. Missing data: sex (*n*=34); ethnicity (*n*=25,193); resident in local catchment area (*n*=6,604); quartiles of neighbourhood deprivation (*n*=8,125); referral status (*n*=713).

For peer review only

# The Impact of Matching Error on Linked Mortality Outcome in a Data Linkage of Secondary Mental Health Data with Hospital Episode Statistics (HES) and Mortality Records in South East London: A Cross-Sectional Study



| | |
|---|---|
| Journal: | *BMJ Open* |
| Manuscript ID | bmjopen-2019-035884.R1 |
| Article Type: | Original research |
| Date Submitted by the Author: | 13-Feb-2020 |



| | |
|---|---|
| Complete List of Authors: | Jewell, Amelia; South London and Maudsley NHS Foundation Trust, Maudsley Biomedical Research Centre (BRC)<br>Broadbent, Matthew; South London and Maudsley NHS Foundation Trust, Maudsley Biomedical Research Centre (BRC)<br>Hayes, Richard; Institute of Psychiatry, Kings College London, Psychological Medicine<br>Gilbert, Ruth; UCL Institute of Child Health, Centre for Paediatric Epidemiology and Biostatistics<br>Stewart, Robert; King's College London, Institute of Psychiatry<br>Downs, Johnny; Kings College London, Institute of Psychiatry, Psychology and Neuroscience |



| | |
|---|---|
| <b>Primary Subject Heading</b>: | Health informatics |
| Secondary Subject Heading: | Mental health |
| Keywords: | Health informatics < BIOTECHNOLOGY & BIOINFORMATICS, MENTAL HEALTH, PSYCHIATRY |
| | |


SCHOLARONE™
Manuscripts

*I, the Submitting Author has the right to grant and does grant on behalf of all authors of the Work (as defined in the below author licence), an exclusive licence and/or a non-exclusive licence for contributions from authors who are: i) UK Crown employees; ii) where BMJ has agreed a CC-BY licence shall apply, and/or iii) in accordance with the terms applicable for US Federal Government officers or employees acting as part of their official duties; on a worldwide, perpetual, irrevocable, royalty-free basis to BMJ Publishing Group Ltd ("BMJ") its licensees and where the relevant Journal is co-owned by BMJ to the co-owners of the Journal, to publish the Work in this journal and any other BMJ products and to exploit all rights, as set out in our licence.*

*The Submitting Author accepts and understands that any supply made under these terms is made by BMJ to the Submitting Author unless you are acting as an employee on behalf of your employer or a postgraduate student of an affiliated institution which is paying any applicable article publishing charge ("APC") for Open Access articles. Where the Submitting Author wishes to make the Work available on an Open Access basis (and intends to pay the relevant APC), the terms of reuse of such Open Access shall be governed by a Creative Commons licence – details of these licences and which Creative Commons licence will apply to this Work are set out in our licence referred to above.*

*Other than as permitted in any relevant BMJ Author's Self Archiving Policies, I confirm this Work has not been accepted for publication elsewhere, is not being considered for publication elsewhere and does not duplicate material already published. I confirm all authors consent to publication of this Work and authorise the granting of this licence.*

For peer review only

# The Impact of Matching Error on Linked Mortality Outcome in a Data Linkage of Secondary Mental Health Data with Hospital Episode Statistics (HES) and Mortality Records in South East London: A Cross-Sectional Study

Amelia Jewell[1*], Matthew Broadbent[1], Richard D Hayes[2], Ruth Gilbert[3], Robert Stewart[1,2], and Johnny Downs[1,2]

* Corresponding author contact information:

Amelia Jewell, Biomedical Research Centre (BRC) Nucleus (PO92), Kings College London, DeCrespigny Park, London, SE5 8AF, United Kingdom. Telephone: +44 20 3228 8554; E-mail: amelia.jewell@slam.nhs.uk

**Author details:**

[1] South London and Maudsley NHS Foundation Trust, London, UK. [2] Institute of Psychiatry, Psychology and Neuroscience, King's College London, London, UK. [3] University College London Great Ormond Street Institute of Child Health.



For peer review only

For peer review only - http://bmjopen.bmj.com/site/about/guidelines.xhtml

## ABSTRACT

**Objectives:** Linkage of electronic health records (EHRs) to Hospital Episode Statistics (HES)-Office for National Statistics (ONS) mortality data has provided compelling evidence for lower life expectancy in people with severe mental illness. However, linkage error may underestimate these estimates. Using a clinical sample (N=265,300) of individuals accessing mental health services, we examined potential biases introduced through missed matching, and examined the impact on the association between clinical disorders and mortality.

**Setting:** The South London and Maudsley NHS Foundation Trust (SLaM) is a secondary mental health care provider in London. A de-identified version of SLaM's EHR was available via the Clinical Record Interactive Search (CRIS) system linked to HES-ONS mortality records.

**Participants:** Records from SLaM for patients active between January 2006 and December 2016.

**Outcome measures:** Two sources of death data were available for SLaM participants: accurate and contemporaneous date of death via local batch tracing (gold standard), and date of death via linked HES-ONS mortality data. The effect of linkage error on mortality estimates was evaluated by comparing sociodemographic and clinical risk factor analyses using gold standard death data, against HES-ONS mortality records.

**Results:** Of the total sample, 93.74% were successfully matched to HES-ONS records. We found a number of statistically significant administrative, socio-demographic, and clinical differences between matched and unmatched records. Of note, schizophrenia diagnosis showed a significant association with higher mortality using gold standard data (OR=1.08; 95% CI=1.01, 1.15; $p$=0.02), but not in HES-ONS data (OR=1.05; 95% CI=0.98, 1.13; $p$=0.16). Otherwise little change was found in the strength of associated risk factors and mortality, after accounting for missed matching bias.

**Conclusions:** Despite significant clinical and socio-demographic differences between matched and unmatched records, changes in mortality estimates were minimal. However, researchers and policy analysts using HES-ONS linked resources should be aware that administrative linkage processes can introduce error.




For peer review only - http://bmjopen.bmj.com/site/about/guidelines.xhtml

**Strengths and limitations of this study:**

- The findings of our study demonstrate that although there are significant differences between matched and unmatched records, these did not significantly change the relative differences in mortality according to type of psychiatric disorder.

- The findings from this study are novel, in that data linkage error has not previously been evaluated in a routine mental health database and this may be useful for other researchers looking to conduct research using electronic health records linked to HES-ONS mortality data by NHS Digital.

- A significant strength of the study is our access to the gold standard death data within the mental health record, which can be used to compare with the linked mortality data; this is useful as the mental health discrepancy in mortality is a particular focus on issues of parity/ inequality for health policy.

- Results may be generalisable to other NHS cohorts being linked to HES-ONS Mortality data via NHS Digital as national standard matching methodology was used for the data linkage and the quality and type of administrative data available for matching is likely to be similar within most NHS Trusts.

- False matches were not examined; therefore, data linkage error within the linked dataset may be higher.

For peer review only

## INTRODUCTION

Individuals with mental health disorders have substantially lower life expectancy in comparison to those without mental health disorders [1,2]. The United Kingdom (UK) government has been working to reduce the mortality gap between those with and without mental health disorders by implementing policies aimed at improving the physical health of individuals with mental illness [3]. To inform changes in health policy it is essential that we have good data available on health (physical and mental) and mortality in order to understand the trends and underlying mechanisms.

Electronic health record (EHR) data from the South London and Maudsley NHS Foundation Trust (SLaM) Clinical Record Interactive Search (CRIS) system [4] linked to Hospital Episode Statistics (HES) and death certificate data from the Office for National Statistics (ONS) have previously been utilised to provide evidence of the mental health mortality gap and investigate potential underlying mechanisms [5-9]. Data linkages such as that between CRIS and HES-ONS mortality resources are an important tool for increasing the use of existing data resources to support research [10]. However, the success of any data linkage and the quality of the subsequent linked dataset rely on multiple factors, including the data quality and linkage methodology. The two main types of linkage error which can occur during the data linkage process are false matches (i.e. false positives) and missed matches (i.e. false negatives). Errors in data linkage may result in systematic bias in reported outcomes [11]. False positives in data linkages can dilute the association between variables [12] whereas false negatives can cause an underestimation of the risk outcome [13]; for example, a US study found that linking on an infant's medical insurance record, as opposed to the mother's, led to a significant underestimation in infant mortality due to the number of missed matches [14].

There are a number of approaches to evaluating linkage quality; for example by comparing characteristics of linked and unlinked records to identify potential sources of bias [12]. Previous studies have identified significant differences in socio-demographic characteristics between matched and unmatched samples in data linkages, including sex [15-21], age [15,19,22-27], ethnicity [16,19,20-22,26-32], socioeconomic status [20,27,33,34], marital status [15,22,28,35], and level of education [20,34,35], providing some indication that linked data may not always be representative of the population of interest [12]. Reference or 'gold standard' datasets in which the true match status is known can be used to evaluate the impact of data linkage error by quantifying missed or false matches [36]; however, gold standard data either do not exist or are rarely available to researchers using linked data due to issues around governance and data protection [12].

If linked data are being used to inform health policy it is important that the effect of linkage error is estimated. At present, for example, it is not clear whether any potential error in the linkage between CRIS and HES-ONS mortality data has a bearing on the research being conducted using the linked data. It was therefore our aim to investigate the effect of linkage error on the prediction of a



For peer review only - http://bmjopen.bmj.com/site/about/guidelines.xhtml

linked outcome measure in this mental health population by, firstly, examining the administrative, socio-demographic, and clinical characteristics of matched and unmatched records, and secondly, by comparing the prediction of linked mortality outcome with gold-standard death data provided by the local mental health record.

## METHODS

*The Mental Health Dataset - CRIS*

The Maudsley Biomedical Research Centre (BRC) CRIS system, described previously in detail [4,37], is a deidentified database of electronic medical records for mental health service users within SLaM: one of the largest providers of secondary mental health care in Europe. SLaM provides local area mental health services predominately for the London boroughs of Lambeth, Southwark, Croydon, and Lewisham (the SLaM 'catchment area'), as well as some national specialist services [4].Records from CRIS have previously been linked to a number of external databases including the National Pupil Database [38], the Thames Cancer Registry [39], and General Practice (GP) data [40].

All individuals (children and adults) who were active in SLaM between 1st January 2006 and 20th December 2016, not including IAPT (Improving Access to Psychological Therapies) services, were included in the current study. Exposure variables (Table 1) were collected from structured fields within the CRIS system and included socio-demographic (e.g. age, sex, ethnicity, and mortality), clinical (e.g. mental health diagnosis, referral status, level of service use), and administrative variables (i.e. patient identifiers available for linkage).

Mortality data from CRIS provided the 'gold standard' death data using both formal and informal notification sources [41]. All-cause mortality data is entered into the SLaM EHR either by the care team directly (for example after being informed by family members) or via automatic tracing, whereby the SLaM Trust run a demographic batch trace weekly for all patients (past or current) against the NHS Summary Care Record (SCR). This returns date of death for those patients who have died (Figure 1). The SCR is an electronic record of important patient information created from GP medical records which can be seen and used by any authorised staff in areas of the health system who are involved in a patients' direct care. The SCR is administered by NHS Digital [42]. Date of death in the SCR is entered at the point of occurrence, i.e. the death may not have been formally registered yet.

*The Hospital Episode Statistics and ONS Mortality Dataset*

HES data are a national dataset, governed by NHS Digital, which holds details of all admissions, outpatient appointments, and accident and emergency attendances at NHS hospitals in England [43]. HES data are derived from the Secondary Uses Service (SUS) database which collects submissions routinely from all NHS acute hospital and mental health trusts in England. The data accuracy and



For peer review only - http://bmjopen.bmj.com/site/about/guidelines.xhtml

quality within these submissions are important, especially to NHS Trusts, as they are used to calculate payments for the care provided [44]. HES data are designed to enable secondary use, e.g. for research, and can be linked to other external data sources.

The ONS mortality data consists of the Primary Care Mortality Database (PCMD) which includes date and cause of death for all deaths registered in England and Wales. The data are collected by the ONS and access to the data is managed by NHS Digital [45]. Mortality data from the PCMD are only returned for individuals who have matched and have died; individuals without a mortality record are therefore assumed to be alive.

*Linkage Procedures*

The linkage between CRIS, HES-ONS mortality data under analysis here was conducted in January 2017. Patient Identifiable Information (PII), i.e. date of birth, sex, NHS number, and postcode, for all SLaM records (excluding anybody who had previously opted out of CRIS) was extracted on 20th December 2016. The data linkage was conducted by NHS Digital following deterministic matching procedures. Date of birth, sex, NHS number, and most recently recorded postcode were used as the personal identifiers to match records. Table 2 demonstrates the match ranks used by NHS Digital, with the quality of the matching decreasing from step one to eight.

Initially records are matched to the Personal Demographics Service (PDS) using this method. The PDS is the national electronic database of NHS patient details such as name, address, date of birth, and NHS number [46]. The PDS is also involved in the creation and updating of the NHS SCR, described above. Theoretically, every CRIS record should match to the PDS, even if they do not have any HES episodes or a mortality record. Following matching to the PDS, patients who have opted out of their data being used for secondary purposes, known as 'type 2' opt-outs [47], are removed from the cohort. A report is produced containing details of all the matched records, excluding unmatched records and opt-outs, known as the Flagging Current Status report. Mortality and HES data are then extracted for matched records only (see Figure 2).

Match status outcome measure (i.e. matched vs. missed match) was determined using the Flagging Current Status report following the linkage of CRIS records by NHS Digital. Unmatched records included both records which were not matched (i.e. missed matches) as well as opt-outs.

For those records which were successfully matched, we examined optimal match rank, whereby an optimal match represents a 'perfect' match or a match rank of one (see Table 2), i.e. the records matched on all supplied patient identifiers. The optimal match variable was provided by NHS Digital within the HES data following completion of the data linkage.

*Patient and Public Involvement*



For peer review only - http://bmjopen.bmj.com/site/about/guidelines.xhtml

Patient and public involvement was key to the initial development and ongoing oversight of the CRIS system. The data linkage between CRIS and the HES-ONS mortality data, as well as this project specifically, were approved by the service-user chaired CRIS Oversight Committee (project reference: 17-065). The CRIS, HES-ONS mortality data linkage was also presented to the Data Linkage Service User and Carer Advisory Group [48], a regular meeting of people with lived experience of mental illness, all of whom have an interest in mental health research involving data linkage. The group provide ongoing advice and feedback to researchers conducting projects using the linked CRIS and HES-ONS mortality data.

*Statistical Analysis*

*Step 1: Missed Matches Analysis*

Data were analysed using STATA version 15. Univariable logistic regression was first performed on all administrative, socio-demographic, and clinical characteristics to examine the association with match status outcome for HES-ONS linkage (i.e. those with a positive match on the Flagging Current Status report). Multivariable logistic regression was then performed to identify factors which remained significant predictors of HES-ONS matching after controlling for all other examined variables.

*Step 2: Optimal Match Analysis*

In order to determine which variables predicted an optimal match, univariable logistic regression analysis was performed on a subsample of the total cohort (i.e. only those records which were matched with HES-ONS). Multivariable logistic regression was then performed to identify factors associated with an optimal match outcome after accounting for all other examined variables.

*Step 3: CRIS Derived Mortality Analysis*

In order to examine the effect of potential matching bias on mortality outcome we first examined factors associated with all-cause mortality using the gold standard CRIS-derived death data in univariable and multivariable analyses.

*Step 4: Linked Mortality Analysis*

We then examined predictors of mortality using the linked mortality outcome (i.e. patients with a death date in the HES-ONS matched dataset), in the HES-ONS matched group only, in univariable and multivariable analyses. We used inverse probability weighting calculated from the Step 1 analysis and the optimal match variable to adjust the final model for missed matching bias.



For peer review only - http://bmjopen.bmj.com/site/about/guidelines.xhtml

**RESULTS**

Within the total cohort (N=265,300), 93.7% (*n*=248,698) of patients were successfully matched by NHS Digital to the HES-ONS data via the PDS. The mean age for the matched sample was 43.40 (Range 5 months to 117 years; SD 22.69), at the time the patient identifiers were extracted, with half the sample being male (50.0%). The majority of the matched group (58.5%) were of white ethnicity and were resident in the four London Boroughs serviced by SLaM, i.e. Lambeth, Southwark, Lewisham, and Croydon (73.4%). The Strengthening The Reporting of Observational studies in Epidemiology (STROBE) checklist [49] is reported in Table 1 of the Supplementary Material.

*Step 1: Missed Matches Analysis*

Table 3 provides the administrative, socio-demographic, and clinical characteristics of the CRIS sample, according to matched and unmatched status. With regards to the administrative variables examined (i.e. NHS number, date of birth, sex, and postcode present in PII sent to NHS Digital), records which did not have an NHS number or date of birth were not matched (*n*=5755, 2.17%). Records where sex was present in the PII sent to NHS Digital were eight times more likely to be matched than records where sex was not present (OR=8.14; 95% CI=4.66, 14.23; $p$<0.001). Similarly, records where a postcode was present were almost seven times more likely to be matched in comparison to records where no postcode was available for matching (OR=6.68; 95% CI= 6.26, 7.12; $p$<0.001). Patients who had died prior to the PII being extracted, i.e. 20$^{th}$ December 2016, (according to the gold standard CRIS-derived death data) were significantly more likely to match than patients who were alive.

Within the adjusted analysis, we found the likelihood of matching was significantly associated with a number of socio-demographic and clinical factors. Males and patients of non-white ethnicities, i.e. (i) mixed, (ii) Indian, Pakistani, Bangladeshi or 'other Asian', (iii) Caribbean, African, or 'other black', or (iv) other ethnicity, were all significantly less likely to match. Individuals without a stated ethnicity were also significantly less likely to match. Compared to patients in the lowest quartile of deprivation, patients in the second quartile were significantly less likely to match. We found no significant differences between patients in the third and fourth quartiles of deprivation ($p$>0.05).

With regards to clinical variables, patients who had been discharged or who had not had a referral in the two years prior to the PII being extracted were significantly less likely to match than patients who had an accepted referral in the past 2 years. Similarly, patients with the most face to face contact (third and fourth quartiles) were significantly more likely to match, whilst patients in the second quartile of face to face contact were significantly less likely to match. Patients with the highest number of inpatient bed days (32+) were significantly more likely to match than those with no inpatient bed days, whereas patients with a moderate number of inpatient bed days (3-31) were significantly less likely to match than those who no inpatient bed days.



For peer review only - http://bmjopen.bmj.com/site/about/guidelines.xhtml

In terms of diagnosis, patients who had ever received a primary diagnosis within the majority F00-98 ICD-10 diagnosis codes (see Table 3) were all significantly more likely to match than patients who had never received an ICD-10 F diagnosis. However, primary diagnoses of schizophrenia, schizotypal, and delusional disorders (F20-F29) or disorders of adult personality and behaviour (F60-F69) were not significantly associated with match status.

*Step 2: Optimal Match Analysis*

For records which were matched (*n*=248,698), we examined predictors of an optimal match. An optimal match represents a 'perfect match', i.e. where the records were matched on all four patient identifiers provided to NHS Digital (NHS number, sex, date of birth, and postcode). Within the adjusted model (see Table 2 in Supplementary Material) older age, male sex, an 'other' or 'not stated' ethnicity, living in the SLaM catchment area, deprivation, referral status in the two years prior to the linkage, low levels of face to face contact, and a mental health diagnosis were all significantly associated with an optimal match. All-cause mortality and number of inpatient bed days were not significantly associated with optimal match outcome.

*Step 3: CRIS Derived Mortality Analysis*

Table 4 displays clinical and socio-demographic factors associated with mortality using the gold standard CRIS-derived death data. Within the fully adjusted model ($aOR^2$) older age, male sex, deprivation, a primary diagnosis ever of organic, including symptomatic, mental disorders (F00-F09), mental and behavioural disorders due to psychoactive substance use (F10-F19), schizophrenia, schizotypal and delusional disorders (F20-F29), mental retardation (F70-F79), amount of face to face contact, and inpatient bed days were all associated with a significant increased risk of mortality.

Whilst, non-white ethnicity, a primary diagnosis ever of mood (affective) disorders (F30-F39), neurotic, stress-related and somatoform disorders (F40-F49), behavioural syndromes associated with physiological disturbances and physical factors (F50-F59), behavioural and emotional disorders with onset usually occurring in childhood and adolescence (F90-F98), or other diagnoses were all associated with a significant decreased risk of mortality.

*Step 4: Linked Mortality Analysis*

We examined the associations between mortality and socio-demographic and clinical factors using the linked HES-ONS mortality data for the matched group only (*n*=248,698). Within the adjusted model (see Table 5) we found that older age, male sex, and the second, third and fourth (i.e. least deprived) quartiles of deprivation were significantly associated with mortality. With regards to clinical variables, a primary diagnosis of organic, including symptomatic, mental disorders (F00-F09), mental



For peer review only - http://bmjopen.bmj.com/site/about/guidelines.xhtml

and behavioural disorders due to psychoactive substance use (F10-F19), mental retardation (F70-F79), and a low, moderate, or high number of inpatient bed days were all significantly associated with an increased risk of mortality. Similarly, patients with higher levels of face to face contact (2nd and 3rd quartiles) were significantly more likely to have died when compared to patients with the least amount of face to face contact.

Non-white ethnicity, and a primary diagnosis of mood (affective) disorders (F30-F39), neurotic, stress-related and somatoform disorders (F40-F49), behavioural syndromes associated with physiological disturbances and physical factors (F50-F59), disorders of psychological development (F80-F89), and behavioural and emotional disorders with onset usually occurring in childhood and adolescence (F90-F98) were all negatively associated with mortality.

After further adjustments, using the optimal match variable and an inverse probability weighting, calculated during the missed match analysis in Step 1, to account for potential linkage bias, we found these associations with mortality persisted (Table 5).

We compared the output of the Step 3 and Step 4 analyses (Table 4 - aOR[1] and Table 5), i.e. predictors of CRIS-derived mortality and predictors of linked ONS mortality in the matched group (controlling for optimal matching and matching probability). The two final models were largely the same with slight differences in some of the odds ratios. However, there was a difference between the models within the diagnosis variables. In the model predicting CRIS-derived mortality, a primary diagnosis ever of schizophrenia, schizotypal and delusional disorders (F20-F29) was significantly associated with all-cause mortality (OR=1.08; 95% CI=1.01, 1.15; $p$=0.02). Whereas in the model predicting linked mortality in the matched group, a primary diagnosis of F20-F29 was not significantly associated with mortality (OR=1.05; 95% CI=0.98, 1.13; $p$>0.05), despite the odds ratios being virtually identical (1.08 for the CRIS-derived death model compared to 1.05 for the linked death model). The only other difference between the two models was regarding the F60-F69 diagnosis variable. The association between a primary diagnosis of a disorder of adult personality and behaviour (F60-F69) and mortality in the CRIS-derived mortality model produced an odds ratio of 1.02, whereas this effect was reversed in the linked mortality model (OR: 0.98); however, neither of these findings were statistically significant.

*Post-hoc Analysis*

In Step 1 of the analysis we found that records which did not have an NHS number or date of birth were not matched. We therefore conducted a post-hoc analysis to investigate the association between sociodemographic factors (i.e. age, sex, ethnicity, resident in the SLaM catchment area, and deprivation) and presence of NHS number and date of birth for matching.



For peer review only - http://bmjopen.bmj.com/site/about/guidelines.xhtml

With regards to NHS number, 5755 (2.17%) individuals did not have an NHS number available for matching. After conducting a multivariate logistic regression analysis (see Table 3 in Supplementary Material), we found that all the sociodemographic variables examined were significantly associated with the availability of NHS number. Specifically, older age, mixed ethnicity, and being resident in the SLaM catchment area were positively associated with having an NHS number in the electronic patient record (EPR). Whereas, male sex, non-white ethnicity (expect 'mixed') and the 2$^{nd}$ and 4th (least deprived) quartiles of deprivation were all negatively associated with having an NHS number in the EPR.

In terms of date of birth, only 290 (0.11%) records were missing a date of birth. In univariate logistic regression analysis (see Table 4 in Supplementary Material) Asian and 'Other' ethnicities were negatively associated with having a date of birth whilst the 2nd and 4th (last deprived) quartiles of deprivation were positively associated with having a date of birth in the EPR. Despite this, none of the sociodemographic variables examined were significantly associated with the availability of date of birth for matching when examined in multivariate logistic regression analysis.

## DISCUSSION

*Statement of the Principal Findings*

By evaluating NHS electronic health records linked via standard deterministic procedures to HES-ONS mortality data, we found no evidence of substantial bias distorting risk-factor associations with the linked outcome measure analysed (i.e. mortality). Of the 265,300 SLaM records sent to NHS Digital for matching, 93.74% were successfully matched. Despite a number of significant administrative, socio-demographic, and clinical differences between records that matched and those that did not, we found minimal effects on the clinical associations with mortality. Matching error did not appear to impact the strength and direction of effects between mortality and a number of socio-demographic and clinical factors using linked mortality data after adjusting for matching probability using an inverse probability weighting statistical technique. Furthermore, after comparing the fully adjusted models predicting gold standard CRIS-derived mortality in the full cohort and linked mortality in the matched group we found that the two models were largely the same with only slight differences in some of the odds ratios.

*Putting the Results in Context*

Consistent with previous research, we found a number of significant socio-demographic differences between the matched and unmatched group including gender, ethnicity, and deprivation. Previous research has demonstrated how linkage error disproportionately affects marginalised people and, therefore, missed matches result in underestimating their needs [16,19-22,26-34]. In comparison



For peer review only - http://bmjopen.bmj.com/site/about/guidelines.xhtml

to the white ethnic group (i.e. British, Irish, or any other white ethnic groups), we found that individuals of all other recorded ethnicities examined were significantly less likely to match. This is potentially relevant to researchers examining health outcome variations by ethnicity, and those conducting studies within similarly diverse, urban populations [4]. We found that being resident in the SLaM catchment area was not associated with match status after controlling for all other examined sociodemographic and clinical factors. Generally, patients receiving care from SLaM national specialist services, as opposed to local area services, are of increased complexity and greater clinical contact which tends to improve administrative accuracy however, this does not seem to have had an impact on match status in the current data linkage.

This is one of the first studies to examine clinical factors associated with matching quality in a mental healthcare population and we identified a number of novel findings with regards to the clinical characteristics of the matched and unmatched groups. Patients who had not had a clinical contact with SLaM in the two years prior to the linkage were significantly less likely to be matched, whereas, patients with high levels of clinical contact, indicated by frequency of face to face contact or inpatient bed days, were significantly more likely to match. Similarly, patients who had ever received a primary diagnosis within the majority F00-98 ICD-10 diagnosis codes were significantly more likely to match than patients who had never received an ICD-10 F diagnosis. Our findings provide some indication that patients with higher severity of illness and clinical service contacts proximal to the date of matching are more likely to match, possibly through these factors driving greater administrative data accuracy. Risk factors for match status may operate through different mechanisms, e.g. (i) through the type of condition and severity, (ii) as determinants of linkage error, or (iii) both, and further research is required to understand the underlying mechanisms.

Improving the quality of data linkages, by reducing missing information in the source data, is important for scientific as well as legal and ethical reasons [25]. Within the current study we found that records which did not contain an NHS number or date of birth in the source SLaM data were not matched. In post-hoc analysis we found that the presence of NHS number in the EPR was significantly associated with a number of sociodemographic factors, including age, sex, and ethnicity. These findings give some indication that there is a bias in the recording of NHS numbers. Future work should aim to examine the reasons why some records are missing important administrative data in order to reduce the number of missing NHS numbers and dates of birth which would subsequently help improve the missed match rate and quality of future data linkages.

The evaluation of linkage quality can guide decisions about appropriate study designs [12]. However, data linkages tend to be carried out by an independent body and information about linkage processes are not always made available to researchers, known as the ‘separation principle’ [50]. By taking account of matching rank, in our sample we found that variation in linkage quality appeared to



For peer review only - http://bmjopen.bmj.com/site/about/guidelines.xhtml

have very little effect on the prediction of mortality. We were able to demonstrate this as the information on missed matches and match rank was provided to us by NHS Digital. Key to health researchers being able to account for the impact of matching on the analysis of interest (in our case clinical risk factor for death), are organisations which conduct data linkages being able to provide information on match accuracy (i.e. match rates, match ranks, etc.) so that potential linkage error can be accounted for in subsequent analyses. In the current study, matching was on a one to one basis (i.e. an individual was matched to a single record on the PDS), however, it is worth noting that in circumstances where matching is not one to one (e.g. matching individuals to multiple episodes of care) controlling for linkage error is more problematic even when information on match accuracy is provided.

*Strengths and Weaknesses of the Study*

This is a large scale study of linkage error in a population of people receiving mental healthcare and one of the first studies to examine the association between patient characteristics and an optimal match. An optimal match (i.e. a match rank of one - see Table 2) indicates a higher quality match and therefore improving the number of records which achieve an optimal match is one way of improving the validity of data linkages. By identifying factors associated with an optimal match we can target specific records in the source data which can be improved (e.g. adding in missing information) in order to maximise the quality of the data linkage.

Gold standard data are rarely available but provide an easily interpretable way to measure linkage error [12], a major strength of the study is the use of gold standard death data (i.e. CRIS-derived mortality) as a reference category for examining error in the linked records. Within the gold-standard data we would expect the majority of deaths to be picked up either via automatic tracing or through the Trust being informed directly (for example via family members), however there may be situations where the Trust is not aware of the death of a patient, for example if the patient died outside of the UK. Furthermore, matching error due to false or missed matches is not the only reason that we may see discrepancies between the CRIS-derived mortality data and the linked mortality data from NHS Digital. For example, if the death was not registered in the UK it would not be included in the mortality data obtained from the ONS. Similarly, there are sometimes delays in death registration due to coroner investigations which can take several months. Although SLaM may be informed at the time of death, and therefore the EPR updated to reflect this, it could be several months before the official registration takes places. Despite this, it is not always possible to identify potential missing death data or the reasons for discrepancies within the data and therefore researchers should be aware of these issues when analysing linked data.



For peer review only - http://bmjopen.bmj.com/site/about/guidelines.xhtml

There are some limitations of this study. The study sample was largely derived from a diverse urban and semi-urban population in South London, and is therefore not nationally representative. However, the findings may be generalisable to other NHS cohorts, as the quality and type of administrative data available for matching is likely to be similar within most NHS Trusts, and national standard NHS Digital matching methodology was used for the data linkage. Future studies should aim to replicate this work using linked administrative data in different settings in order to check for the reproducibility of findings. A further limitation is that within the unmatched group we were unable to tell apart missed matches and patients who had opted out of their data being used for secondary purposes. This is due to the strict governance requirements around opt-outs [47]. However, any researcher using data from NHS Digital, will have to account for missed matching in the same way, irrespective of whether non-matching was due to opt-out. Within the current study, it was not possible for us to tease apart the impact of providing a full or part postcode on match status. In future it may be beneficial to separate the postcode variable in order to determine the impact of full and part postcodes on matching.

The data linkage in the current study was conducted by NHS Digital using deterministic methods; however, previous research has found that following probabilistic linkage methods can reduce the number of missed matches [26]. Therefore, the inclusion of probabilistic matching techniques may help to improve linkage error in future refreshes of the data linkage. Furthermore, within the current study we only examined missed matches and not false matches, and therefore we may have underestimated the effect of linkage error. Future research should aim to examine the rate of false matches in order to gain a clearer picture of the total effect of linkage error in this population.

*Conclusion*

There are significant administrative, socio-demographic, and clinical differences between the matched and unmatched records in the data linkage examined. Despite this, after adjusting for matching probability, missed matches do not appear to have had an effect on the prediction of mortality. Furthermore, after comparing models predicting gold standard CRIS-derived mortality and linked mortality we found that the models were largely the same with only slight differences in some of the odds ratios. Together these provide some indication that this effect is not driven by selection bias from matching error and provide some reassurance to studies using NHS linked data to investigate mortality.



For peer review only - http://bmjopen.bmj.com/site/about/guidelines.xhtml

**Acknowledgements**

We would like to acknowledge NHS Digital as the provider of the HES and mortality data; those who carried out the original collection and analysis of the data bear no responsibility for their further analysis or interpretation. We would also like to acknowledge the staff at the Maudsley Biomedical Research Centre (BRC) Nucleus for their continued support of the study.

**Contributors**

AJ and JD conceived the study design. Data extraction was completed by AJ with support from JD and MB. Data analysis was conducted by AJ and JD. Reporting of findings was led by AJ and JD with support from MB, RG, RH, and RS. All authors contributed to the manuscript preparation and approved the final version.

**Funding**

This paper represents independent research part funded by the National Institute for Health Research (NIHR) Biomedical Research Centre (BRC) at the South London and Maudsley NHS Foundation Trust and King's College London. The work described here was part-supported by the Mental Health Data Pathfinder award to King's College London from the Medical Research Council (reference MC_PC_17214). The views expressed are those of the authors and not necessarily those of the NHS, the NIHR, the MRC, or the Department of Health. AJ, MB, RH, and RS receive salary support from the National Institute for Health Research (NIHR) Mental Health Biomedical Research Centre at South London and Maudsley NHS Foundation Trust and King's College London.

**Competing Interests**

RH has received research funding from Roche, Pfizer, Janssen, and Lundbeck. The other authors have declared that no competing interests exist.

**Ethics Approval**

CRIS was approved as a dataset for secondary analysis by the Oxford Research Ethics Committee C (18/SC/0372). Approval to conduct the data linkage was granted by the Health Research Authority (HRA) Confidentiality Advisory Group (CAG) in July 2011 (ECC 3-04(f)/2011) and by NHS Digital's Data Access Advisory Group (DAAG) in October 2016 (NIC-292279-Z2S5T). Approval to conduct the study was granted in July 2017 by the CRIS Oversight Committee (17-065).

**Data Sharing Statement**



For peer review only - http://bmjopen.bmj.com/site/about/guidelines.xhtml

For peer review only

Data are owned by a third party, Maudsley Biomedical Research Centre (BRC) Clinical Records Interactive Search (CRIS) tool, which provides access to anonymised data derived from SLaM electronic medical records. These data can only be accessed by permitted individuals from within a secure firewall (i.e., the data cannot be sent elsewhere), in the same manner as the authors. For more information please contact: cris.administrator@slam.nhs.uk.

For peer review only



For peer review only - http://bmjopen.bmj.com/site/about/guidelines.xhtml

**REFERENCES**

1. Chesney, E., Goodwin, G.M. and Fazel, S. (2014) Risks of all-cause and suicide mortality in mental disorders: a meta-review. *World Psychiatry*, 13, p.p. 153-160.

2. Walker, E.R., McGee, R.E. and Druss, B.G. (2015) Mortality in Mental Disorders and Global Disease Burden Implications: A Systematic Review and Meta-analysis. *JAMA Psychiatry*, 72(4), p.p. 334-341.

3. Ilyas, A., Chesney, E. and Patel, R. (2017) Improving life expectancy in people with serious mental illness: should we place more emphasis on primary prevention. *The British Journal of Psychiatry*, 211(4), p.p. 194-197.

4. Perera, G., Broadbent, M., Callard, F., Chang, C-K., Downs, J., Dutta, R., Fernandes, A., Hayes, R.D., Henderson, M., Jackson, R., Jewell, A., Kadra, G., Little, R., Pritchard, M., Shetty, H., Tulloch, A., Stewart, R. (2016) Cohort profile of the South London and Maudsley NHS Foundation Trust Biomedical Research Centre (SLaM BRC) Case Register: current status and recent enhancement of an Electronic Mental Health Record- derived data resource. *BMJ Open*, 6:e008721.

5. Chang, C-K., Hayes, R.D., Perera, G., Broadbent, M.T.M., Fernandes, A.C., Lee, W.E., Hotopf, M. and Stewart, R. (2011) Life Expectancy at Birth for People with Serious Mental Illness and Other Major Disorders from a Secondary Mental Health Care Case Register in London. *PLoS ONE*, 6(5): e19590.

6. Fok, M.L-Y, Hayes, R.D., Chang, C-K., Stewart, R., Callard, F.J. and Moran, P. (2012) Life expectancy at birth and all-cause mortality among people with personality disorder. *Journal of Psychosomatic Research*, 73(2), p.p. 104-107.

7. Hayes, R.D., Chang, C-K., Fernandes, A., Begum, A., To, D., Broadbent, M., Hotopf, M. and Stewart, R. (2012) Associations between symptoms and all-cause mortality in individuals with serious mental illness. *Journal of Psychosomatic Research*, 72(2), p.p. 114-119.



For peer review only

For peer review only - http://bmjopen.bmj.com/site/about/guidelines.xhtml

8. Das-Munshi, J., Chang, C-K., Dutta, R., Morgan, C., Nazroo, J., Stewart, R. and Prince, M.J. (2017) Ethnicity and excess mortality in severe mental illness: a cohort study. *The Lancet Psychiatry*, 4, p.p. 389-399.

9. Jayatilleke, N., Hayes, R.D., Dutta, R., Shetty, H., Hotopf, M., Chang, C-K. and Stewart, R. (2017) Contributions of specific causes of death to lost life expectancy in severe mental illness. *European Psychiatry*, 43, p.p. 109-115.

10. Green, E., Ritchie, F., Mytton, J., Webber, D.J., Deave, T., Montgomery, A., Woolfrey, L., ul-Baset, K. and Chowdhury, S. (2015) Enabling Data Linkage to Maximise the Value of Public Health Research Data: full report. Project Report. Wellcome Trust, London. [Online] Available from: https://wellcome.ac.uk/sites/default/files/enabling-data-linkage-to-maximise-value-of-public-health-research-data-phrdf-mar15.pdf. [Accessed 7 March 2018].

11. Bohensky, M.A., Jolley, D., Sundararajan, V., Evans, S., Pilcher, D.V., Scott, I. and Brand, C.A. (2010) Data Linkage: A powerful research tool with potential problems. *BMC Health Services Research*, 10:346.

12. Harron, K.L., Doidge, J.C., Knight, H.E., Gilbert, R.E., Goldstein, H., Cromwell, D.A. and van der Meulen, J.H. (2017) A guide to evaluating linkage quality for the analysis of linked data. *International Journal of Epidemiology*, 0 (0), p.p. 1-12.

13. Blakely, T. and Salmond, C. (2002) Probabilistic record linkage and a method to calculate the positive predictive value. *International Journal of Epidemiology*, 31, p.p. 1246-1252.

14. Buescher, P.A. (1999) Method of Linking Medicaid Records to Birth Certificates May Affect Infant Outcome Statistics. *American Journal of Public Health*, 89(4), p.p. 564-566.

15. Bopp, M. and Minder, C.E. (2003) Mortality by education in German speaking Switzerland, 1990-1997: results from the Swizz National Cohort. *International Epidemiological Association*, 32, p.p. 346-354.

16. Zingmond, D.S., Ye, Z., Ettner, S.L. and Honghu, L. (2004) Linking hospital discharge and death records - accuracy and sources of bias. *Journal of Clinical Epidemiology*, 57, p.p. 21-29.



For peer review only - http://bmjopen.bmj.com/site/about/guidelines.xhtml

17. Jebamani, L.S., Burchill, C.A. and Martens, P.J. (2005) Using data linkage to identify First National Manitobans: technical, ethical, and political issues. *Canadian Journal of Public Health*, 96(1): S28-32.

18. Maizlish, N.A. and Herrera, L. (2005) A Record Linkage Protocol for a Diabetes Registry at Ethnically Diverse Community Health Centers. *Journal of the American Medical Informatics Association*, 12(3), p.p. 331-337.

19. Schmidlin, K., Clough-Gorr, K.M., Spoerri, A., Egger, M. and Zwahlen, M. (2013) Impact of unlinked deaths and coding changes on mortality trends in the Swiss National Cohort. *BMC Medical Informatics and Decision Making*, 13:1.

20. Kabudula, C.W., Clark, B.D., Gómez-Olive, F.X., Tollman, S., Menken, J. and Reniers, G. (2014) The promise of record linkage for assessing the uptake of health services in resource constrained settings: a pilot study from South Africa. *BMC Medical Research Methodology*, 14:71.

21. McIsaac, K.E., MacDonald, S.F., Chong, N., Moser, A., Moineddin, R., Colantonio, A., Nathens, A. and Matheson, F.I. (2016) Creating a powerful platform to explore health in a correctional population: A record linkage study. *PLOS one*, 11(8): e0161173.

22. Rosman, D.L. and Knuiman, M.W. (1994) A comparison of hospital and police road injury data. *Accident; Analysis and Prevention,* 26(2), p.p. 215-222.

23. Cryer, P.C., Westrup, S., Cook, A.C., Ashwell, V., Bridger, P. and Clarke, C. (2001) Investigation of bias after data linkage of hospital admissions data to police road traffic crash reports. *Injury Prevention,* 7, p.p. 234-241.

24. Clark, D., King, A., Sharpe, K., Connelly, G., Elliot, L., Macpherson, L.M.D., McMahon, A.D., Milligan, I., Wilson, P., Conway, D.I. and Wood, R. (2017) Linking routinely collected social work, education and health data to enable monitoring of the health and health care of school-aged children in state care ('looked after children') in Scotland: a national demonstration project. *Public Health*, 150, p.p. 101-111.



For peer review only
For peer review only - http://bmjopen.bmj.com/site/about/guidelines.xhtml

25. Hagger-Johnson, G., Harron, K., Fleming, T., Gilbert, R., Goldstein, H., Landy, R. and Parslow, R.C. (2015) Data linkage errors in hospital administrative data when applying a pseudonymisation algorithm to paediatric intensive care records. *BMJ Open*, 5:e008118.

26. Hagger-Johnson, G., Harron, K., Goldstein, H., Aldridge, R. and Gilbert, R. (2017) Probabilistic linking to enhance deterministic algorithms and reduce linkage errors in hospital administrative data. *Journal of Innovation in Health Informatics*, 24(2), p.p. 234-246.

27. Downs, J.M., Ford, T., Stewart, R., Epstein, S., Shetty, H., Little, R., Jewell, A., Broadbent, M., Deighton, J., Mostafa, T., Gilbert, R., Hotopf, M. and Hayes, R. (2019) An approach to linking education, social care and electronic health records for children and young people in South London: a linkage study of child and adolescent mental health service data. *BMJ Open*, 9:e024355.

28. Adams, M.M., Wilson, H.G., Casto, D.L., Berg, C.J., McDermott, J.M., Gaudino, J.A. and McCarthy, B.J. (1997) Constructing Reproductive Histories by Linking Vital Records. *American Journal of Epidemiology*, 145(4), p.p. 339-348.

29. Blakely, T., Woodward, A. and Salmond, C. (2000) Anonymous linkage of New Zealand mortality and Census data. *Australian and New Zealand Journal of Public Health,* 24(1), p.p. 92-95.

30. Gyllstrom, M.E., Jensen, J.L., Vaughan, J.N., Castellano, S.E. and Oswald, J.W. (2002) Linking Birth Certificates with Medicaid Data to Enhance Population Health Assessment: Methodological Issues Addressed. *Journal of Public Health Management Practice,* 8(4), p.p. 38-44.

31. Ringland, C., Correll, P.K., Lim, K., Williamson, M. and Marks, G.B. (2006) Hospital readmissions for asthma: a feasibility study comparing strategies for linking hospital morbidity data. *Australian and New Zealand Journal of Public Health,* 30(5), p.p. 435-439.

32. Lariscy, J.T. (2011) Differential Record Linkage by Hispanic Ethnicity and Age in Linked Mortality Studies: Implications for the Epidemiologic Paradox. *Journal of Aging and Health*, 23(8), p.p. 1263-1284.



For peer review only - http://bmjopen.bmj.com/site/about/guidelines.xhtml

33. Beckmann, K.R., Bennett, A., Young, G.P. and Roder, D.M. (2014) Treatment patterns among colorectal cancer patients in South Australia: a demonstration of the utility of population-based data linkage. *Journal of Evaluation in Clinical Practice*, 20, p.p. 467-477.

34. Moore, C.L., Gidding, H.F., Law, M.G. and Amin, J. (2016) Poor record linkage sensitivity biased outcomes in a linked cohort analysis. *Journal of Clinical Epidemiology*, 75, p.p. 70-77.

35. Baldwin, E., Johnson, K., Berthoud, H. and Dublin, S. (2015) Linking mothers and infants within electronic health records: a comparison of deterministic and probabilistic algorithms. *Pharmacoepidemiology and Drug Safety*, 24, p.p. 45-51.

36. Harron, K., Dibben, C., Boyd, J., Hjern, A., Azimaee, M., Barreto, M.L. and Goldstein, H. (2017) Challenges in administrative data linkage for research. *Big Data & Society*, 4(2), p.p. 1-12.

37. Stewart, R., Soremekun, M., Perera, G., Broadbent, M., Callard, F., Denis, M., Hotopf, M., Thornicroft, G. and Lovestone, S. (2009) The South London and Maudsley NHS Foundation Trust Biomedical Research Centre (SLAM BRC) case register: development and descriptive data. *BMC Psychiatry*, 9:51.

38. Downs, J., Gilbert, R., Hayes, R.D., Hotopf, M. and Ford, T. (2017) Linking health and education data to plan evaluate services for children. *Archives of Disease in Childhood*, 102, p.p. 599-602.

39. Chang, C-K., Hayes, R.D., Broadbent, M.T.M., Hotopf, M., Davies, E., Møller, H. and Stewart, R. (2014) A cohort study on mental disorders, stage of cancer at diagnosis and subsequent survival. *BMJ Open*, 4:e004295.

40. Woodhead, C., Ashworth, M., Broadbent, M., Callard, F., Hotopf, M., Schofield, P., Soncul, M., Stewart, R.J. and Henderson, M.J. (2016) Cardiovascular disease treatment among patients with server mental illness: a data linkage study between primary and secondary care. British *Journal of General Practice*, 66, p.p. e374-e381.

41. NHS (2019) PDS Death Notification Status Code; Description. [Online] Available from: https://www.datadictionary.nhs.uk/data_dictionary/attributes/p/paya/pds_death_notification_status_code_de.asp?shownav=1



For peer review only - http://bmjopen.bmj.com/site/about/guidelines.xhtml

42. NHS Digital (2019) Summary Care Records (SCR). [Online] Available from: https://digital.nhs.uk/services/summary-care-records-scr [Accessed 16 July 2019].

43. Academy of Medical Royal Collages. Hospital Episode Statistics (HES): improving the quality and value of hospital data. A discussion document. Leeds: Health and Social Care Information Centre, 2011. [Online] Available from: http://content.digital.nhs.uk/media/1593/Hospital-Episode-Statistics-Improving-the-quality-and-value-of-hospital-data/pdf/HES__Improving_the_quality_and_value_of__hospital_data.pdf [Accessed 9 February 2018].

44. NHS Digital (2019) Secondary Uses Service (SUS). [Online] Available from: https://digital.nhs.uk/services/secondary-uses-service-sus. [Accessed 18 January 2019].

45. NHS Digital (2019) Primary Care Mortality Database. [Online] Available from: https://digital.nhs.uk/services/primary-care-mortality-database. [Accessed 16 July 2019].

46. NHS Digital (2019) Demographics. [Online] Available from: https://digital.nhs.uk/services/demographics. [Accessed 2 April 2019].

47. NHS Digital (2018) Information on type 2 opt-outs. [Online] Available from: https://digital.nhs.uk/data-access-request-service/type-2-opt-outs. [Accessed 6 March 2018].

48. Jewell, A., Pritchard, M., Barrett, K., Green, P., Markham, S., McKenzie, S., Oliver, R., Wan, M., Downs, J. and Stewart, R. (2019) The Maudsley Biomedical Research Centre (BRC) data linkage service user and carer advisory group: creating and sustaining a successful patient and public involvement group to guide research in a complex area. *Research Engagement and Involvement*, 5:20.

49. Elm, E.V., Altman, D.G., Egger, M., Pocock, S.J., Gøtzche, P.C., Vanderbroucke, J.P., STROBE Initiative (2007) Strengthening the reporting of observational studies in epidemiology (STROBE) statement: guidelines for reporting observational studies. BMJ, 335:806.



For peer review only - http://bmjopen.bmj.com/site/about/guidelines.xhtml

50. Gilbert, R., Lafferty, R., Hagger-Johnson, G., Harron, K., Zhang, L-C, Smith, P., Dibben, C. and Goldstein, H. (2018) GUILD: GUidance for Information about Linking Data sets. *Journal of Public Health*, 40(1), p.p. 191-198.

For peer review only



For peer review only - http://bmjopen.bmj.com/site/about/guidelines.xhtml

**Figure Captions**

**Figure 1.** Flow diagram describing how the gold standard CRIS-derived date of death is entered into the SLaM electronic medical record.

**Figure 2.** Flow diagram of the linkage process between SLaM records and HES- ONS mortality data.

For peer review only



For peer review only - http://bmjopen.bmj.com/site/about/guidelines.xhtml

**Table 1.** Exposure variables.

| **Exposure Variable** | **Description** |
|---|---|
| NHS number present | Was NHS number available for linkage, binary variable (yes vs. no). |
| Date of birth present | Was date of birth available for linkage, binary variable (yes vs. no). |
| Sex present | Was sex available for linkage, binary variable (yes vs. no). |
| Postcode present | Was postcode (full or part) available for linkage, binary variable (yes vs. no). |
| Age | Age in years at time of PII extraction (20$^{th}$ December 2016). |
| Sex | Sex, male vs. female. |
| Patient deceased | Is the patient deceased according to the gold standard CRIS-derived mortality data (yes vs. no). |
| Ethnicity | Patient ethnicity, coded into 6 categories (i) British, Irish, or any other white ethnic groups, (ii) mixed, (iii) Indian , Pakistani, Bangladeshi, or 'other Asian', (iv) Caribbean, African, or 'other black', (v) other, and (vi) not stated. |
| Resident in SLaM catchment area | Was the patient resident in the SLaM catchment area, i.e. Lambeth, Lewisham, Southwark, or Croydon, at the time of PII extraction, binary variable (yes vs. no). |
| Quartiles of neighbourhood deprivation | Quartiles of deprivation score: 1$^{st}$ (most deprived), 2$^{nd}$, 3$^{rd}$, 4$^{th}$ (least deprived). |
| Referral Status in past two years | Referral status in the two years prior to PII extraction; (i) accepted, (ii) discharged, (iii) rejected, or (iv) no referral in 2 years prior to linkage. |



For peer review only - http://bmjopen.bmj.com/site/about/guidelines.xhtml

| | |
|---|---|
| Primary Diagnosis Ever | A primary diagnosis ever of F00-F09: organic, including symptomatic, mental disorders, F10-F19: mental and behavioural disorders due to psychoactive substance use, F20-F29: schizophrenia, schizotypal and delusional disorders, F30-F39: mood (affective) disorders, F40-F49: neurotic, stress-related and somatoform disorders, F50-F59: behavioural syndromes associated with physiological disturbances and physical factors, F60-F69: disorders of adult personality and behaviour, F70-F79: mental retardation, F80-89: disorders of psychological development, F90-F98: behavioural and emotional disorders with onset usually occurring in childhood and adolescence, or Other diagnosis. Binary variables (yes vs. no). Other diagnosis category includes all non-mental health (i.e. non F code) ICD-10 diagnoses as well as 'F99: Mental disorder, not otherwise specified' and 'F00-F99: Mental and behavioural disorders'. Patients can have multiple primary diagnoses throughout their time at SLaM and therefore can appear in multiple groups. |
| Face to face contact | Quartiles of face to face contact with SLaM from 1st January 2006 to 20th December 2016; 1st (least face to face contact), 2nd, 3rd, 4th (most face to face contact). |
| Inpatient bed days | Number of SLaM inpatient bed days from 1st January 2006 to 20th December 2016, coded into; (i) none (0), (ii) low (1-2 days), (iii) moderate (3-31 days), and (iv) high (32+). |
| Optimal match | Binary variable (optimal match vs. non-optimal match). For those records which were successfully matched by NHS Digital only. An optimal match represents a 'perfect' match or a match rank of one (see Table 2), i.e. the records matched on all supplied patient identifiers (NHS number, sex, date of birth, and postcode). |



For peer review only - http://bmjopen.bmj.com/site/about/guidelines.xhtml

**Table 2.** NHS Digital deterministic matching steps.

| Match Rank | NHS Number | Date of Birth | Sex | Postcode[a] | |
|---|---|---|---|---|---|
| 1 | Exact | Exact | Exact | Exact | |
| 2 | Exact | Exact | Exact | | |
| 3 | Exact | Partial | Exact | Exact | |
| 4 | Exact | Partial | Exact | | |
| 5 | Exact | | | Exact | |
| 6 | | Exact | Exact | Exact | where NHS number does not contradict the match and DOB is not 1 January and the Postcode is in the 'ignore' list[b] |
| 7 | | Exact | Exact | Exact | where NHS number does not contradict the match and DOB is not 1 January |
| 8 | Exact | | | | |

**Note.** [a] Current or most recently recorded postcode. [b] The 'ignore' list includes postcodes for communal establishments such as hospitals, care homes, prisons, and boarding schools.



For peer review only - http://bmjopen.bmj.com/site/about/guidelines.xhtml

**Table 3.** Logistic regression analysis examining administrative, socio-demographic, and clinical associations with match status (match vs. missed match).

| Variable | Total Population (N=265,300) | Matched (*n*=248,698 93.74%) | Missed Match (*n*=16,602 6.26%) | OR (95% CI) | aOR[1] (95% CI) |
|---|---|---|---|---|---|
| **Administrative variables** | | | | | |
| NHS number present: *n* (%) | 259545 (97.83) | 248698 (100.00) | 10847 (65.34) | | |
| Date of birth present: *n* (%) | 265010 (99.89) | 248698 (100.00) | 16312 (98.25) | | |
| Sex present: *n* (%) | 265246 (99.98) | 248663 (99.99) | 16583 (99.89) | **8.14 (4.66-14.23)**** | |
| Postcode present: *n* (%) | 260466 (98.18) | 245278 (98.62) | 15188 (91.48) | **6.68 (6.26-7.12)**** | |
| **Sociodemographic variables** | | | | | |
| Age: mean (SD) | 43.40 (22.69) | 43.50 (22.79) | 41.92 (21.09) | **1.003 (1.002-1.004)**** | 0.999 (0.998-1.000) |
| Male Sex: *n* (%) | 132730 (50.04) | 123818 (49.79) | 8912 (53.74) | **0.85 (0.83-0.88)**** | **0.93 (0.89-0.96)**** |
| Patient deceased: *n* (%) | 30173 (11.37) | 29057 (11.68) | 1116 (6.72) | **1.84 (1.73-1.95)**** | **1.41 (1.30-1.53)**** |
| Ethnicity: *n* (%) | | | | | |
| British, Irish, or any other white ethnic groups | 138495 (58.54) | 131150 (58.93) | 7345 (52.35) | (reference) | (reference) |
| Mixed | 6853 (2.90) | 6477 (2.91) | 376 (2.68) | 0.96 (0.87-1.07) | **0.82 (0.73-0.91)**** |
| Indian, Pakistani, Bangladeshi, or ‘other Asian’ | 10889 (4.60) | 10155 (4.56) | 734 (5.23) | **0.77 (0.72-0.84)**** | **0.78 (0.71-0.85)**** |
| Caribbean, African, or ‘other black’ | 40725 (17.21) | 38013 (17.08) | 2712 (19.33) | **0.78 (0.75-0.82)**** | **0.70 (0.66-0.74)**** |
| Other | 16650 (7.04) | 15393 (6.92) | 1257 (8.96) | **0.69 (0.64-0.73)**** | **0.77 (0.72-0.83)**** |
| Not stated | 22961 (9.71) | 21354 (9.60) | 1607 (11.45) | **0.74 (0.70-0.79)**** | **0.89 (0.83-0.95)*** |
| Resident in SLaM catchment area: *n* (%) | 187773 (73.42) | 177766 (73.53) | 10007 (71.47) | **1.11 (1.07-1.15)**** | 0.97 (0.93-1.02) |
| Quartiles of neighbourhood deprivation: *n* (%) | | | | | |
| 1st (most deprived) | 63476 (25.03) | 60220 (25.09) | 3256 (24.00) | (reference) | (reference) |
| 2nd | 63452 (25.02) | 59786 (24.91) | 3666 (27.02) | **0.88 (0.84-0.93)**** | **0.90 (0.86-0.95)**** |
| 3rd | 63449 (25.02) | 60052 (25.02) | 3397 (25.04) | 0.96 (0.91-1.00) | 1.01 (0.95-1.07) |
| 4th (least deprived) | 63221 (24.93) | 59973 (24.99) | 3248 (23.94) | 1.00 (0.95-1.05) | 1.06 (1.00-1.12) |
| **Clinical variables** | | | | | |
| Referral status in 2 years prior to linkage: *n* (%) | | | | | |
| Accepted | 12041 (4.56) | 11423 (4.61) | 618 (3.78) | (reference) | (reference) |
| Discharged | 45228 (17.11) | 42394 (17.10) | 2834 (17.33) | **0.81 (0.74-0.89)**** | **0.87 (0.78-0.97)*** |
| Rejected | 9628 (3.64) | 8921 (3.60) | 707 (4.32) | **0.68 (0.61-0.76)**** | 0.88 (0.76-1.01) |



For peer review only - http://bmjopen.bmj.com/site/about/guidelines.xhtml

| | | | | | |
|---|---|---|---|---|---|
| No referral in 2 years prior to linkage | 197414 (74.69) | 185221 (74.70) | 12193 (74.57) | **0.82 (0.76-0.89)*** | **0.87 (0.79-0.95)*** |
| Primary diagnosis ever: *n (%)* | | | | | |
| F00-F09: organic, including symptomatic, mental disorders | 25249 (9.52) | 24248 (9.75) | 1001 (6.03) | **1.68 (1.58-1.80)**** | **1.37 (1.26-1.49)**** |
| F10-F19: mental and behavioural disorders due to psychoactive substance use | 27755 (10.46) | 26158 (10.52) | 1597 (9.62) | **1.10 (1.05-1.16)**** | **1.12 (1.05-1.21)*** |
| F20-F29:schizophrenia, schizotypal and delusional disorders | 18516 (6.98) | 17323 (6.97) | 1193 (7.19) | 0.97 (0.91-1.03) | 1.02 (0.93-1.11) |
| F30-F39: mood (affective) disorders | 42578 (16.05) | 40515 (16.29) | 2063 (12.43) | **1.37 (1.31-1.44)**** | **1.28 (1.21-1.36)**** |
| F40-F49: neurotic, stress-related and somatoform disorders | 33248 (12.53) | 31599 (12.71) | 1649 (9.93) | **1.32 (1.25-1.39)**** | **1.27 (1.19-1.35)**** |
| F50-F59: behavioural syndromes associated with physiological disturbances and physical factors | 8743 (3.30) | 8388 (3.37) | 355 (2.14) | **1.60 (1.43-1.78)**** | **1.49 (1.31-1.69)**** |
| F60-F69: disorders of adult personality and behaviour | 6945 (2.62) | 6585 (2.65) | 360 (2.17) | **1.23 (1.10-1.37)**** | 0.95 (0.84-1.07) |
| F70-F79: mental retardation | 3018 (1.14) | 2898 (1.17) | 120 (0.72) | **1.62 (1.35-1.95)**** | **1.55 (1.26-1.89)**** |
| F80-F89: disorders of psychological development | 7242 (2.73) | 6991 (2.81) | 251 (1.51) | **1.88 (1.66-2.14)**** | **1.86 (1.61-2.15)**** |
| F90-F98: behavioural and emotional disorders with onset usually occurring in childhood and adolescence | 17011 (6.41) | 16363 (6.58) | 648 (3.90) | **1.74 (1.60-1.88)**** | **1.58 (1.45-1.74)**** |
| Other diagnosis | 119638 (45.10) | 112556 (45.26) | 7082 (42.66) | **1.11 (1.08-1.15)**** | **1.23 (1.18-1.28)**** |
| Quartiles of face to face contacts: *n* (%) | | | | | |
| 1st (least face to face contact) | 76602 (28.87) | 70822 (28.48) | 5780 (34.82) | (reference) | (reference) |
| 2nd | 64914 (24.47) | 59811 (24.02) | 5103 (30.74) | **0.96 (0.92-0.99)*** | **0.94 (0.89-0.99)*** |
| 3rd | 59337 (22.37) | 56116 (22.56) | 3221 (19.40) | **1.42 (1.36-1.49)**** | **1.15 (1.08-1.22)**** |
| 4th (most face to face contact) | 64447 (24.29) | 61949 (24.91) | 2498 (15.05) | **2.02 (1.93-2.12)**** | **1.42 (1.33-1.52)**** |
| Inpatient bed days: *n* (%) | | | | | |
| None (0) | 238573 (89.93) | 223527 (89.88) | 15046 (90.63) | (reference) | (reference) |
| Low (1-2 days) | 1446 (0.55) | 1342 (0.54) | 104 (0.63) | 0.87 (0.71-1.06) | 0.86 (0.67-1.09) |
| Moderate (3-31 days) | 9825 (3.70) | 9133 (3.67) | 692 (4.17) | **0.89 (0.82-0.96)*** | **0.90 (0.81-1.00)*** |
| High (32+ days) | 15456 (5.83) | 14696 (5.91) | 760 (4.58) | **1.30 (1.21-1.40)**** | **1.14 (1.03-1.26)*** |

**Note.** *$p$<0.05. **$p$<0.001. [1]adjusted for all other variables listed in the table. Missing data: age (*n*=275); sex (*n*=55); ethnicity (*n*=28,727); resident in local catchment area (*n*=9,537); quartiles of neighbourhood deprivation (*n*=11,702); referral status (*n*=989).



For peer review only - http://bmjopen.bmj.com/site/about/guidelines.xhtml

**Table 4.** Logistic regression analysis examining socio-demographic and clinical associations with gold standard CRIS-derived mortality.

| Variable | Total Population (N=265,300) | Patient Deceased (*n*=30,173 11.37%) | Patient Alive (*n*=235,127 88.63%) | OR (95% CI) | aOR[1] (95% CI) |
|---|---|---|---|---|---|
| **Sociodemographic variables** | | | | | |
| Age: mean (SD) | 43.40 (22.69) | 77.32 (19.06) | 39.05 (19.17) | **1.090 (1.089-1.091)**** | **1.092 (1.090-1.093)**** |
| Male Sex: *n* (%) | 132730 (50.04) | 14848 (49.21) | 117882 (50.15) | **0.96 (0.94-0.99)*** | **1.48 (1.43-1.53)**** |
| Ethnicity coded: *n* (%) | | | | | |
| British, Irish, or any other white ethnic groups | 138495 (58.54) | 21993 (78.19) | 116502 (55.89) | (reference) | (reference) |
| Mixed | 6853 (2.90) | 208 (0.74) | 6645 (3.19) | **0.17 (0.14-0.19)**** | **0.73 (0.61-0.86)**** |
| Indian, Pakistani, Bangladeshi, or 'other Asian' | 10889 (4.60) | 1006 (3.58) | 9883 (4.74) | **0.54 (0.50-0.58)**** | **0.70 (0.65-0.76)**** |
| Caribbean, African, or any 'other black' | 40725 (17.21) | 2914 (10.36) | 37811 (18.14) | **0.41 (0.39-0.43)**** | **0.63 (0.60-0.66)**** |
| Other | 16650 (7.04) | 705 (2.51) | 15945 (7.65) | **0.23 (0.22-0.25)**** | **0.52 (0.48-0.57)**** |
| Not stated | 22961 (9.71) | 1301 (4.63) | 705 (2.51) | **0.32 (0.30-0.34)**** | **0.91 (0.84-0.98)*** |
| Resident in SLaM catchment area: *n* (%) | 187773 (73.42) | 23870 (81.41) | 163903 (72.38) | **1.67 (1.62-1.72)**** | **0.84 (0.80-0.88)**** |
| Quartiles of neighbourhood deprivation: *n* (%) | | | | | |
| 1st (most deprived) | 63476 (25.03 | 7626 (26.11) | 55850 (24.89) | (reference) | (reference) |
| 2nd | 63452 (25.02) | 7049 (24.13) | 56403 (25.14) | **0.92 (0.88-0.95)**** | **1.08 (1.03-1.13)*** |
| 3rd | 63449 (25.02) | 7375 (25.25) | 56074 (24.99) | **0.96 (0.93-1.00)*** | **1.25 (1.19-1.31)**** |
| 4th (least deprived) | 63221 (24.93) | 7161 (24.51) | 56060 (24.98) | **0.94 (0.90-0.97)**** | **1.31 (1.25-1.37)**** |
| **Clinical variables** | | | | | |
| Primary diagnosis ever: *n (%)* | | | | | |
| F00-F09: organic, including symptomatic, mental disorders | 25249 (9.52) | 12641 (41.90) | 12608 (5.36) | **12.73 (12.36-13.10)**** | **1.10 (1.05-1.15)**** |
| F10-F19: mental and behavioural disorders due to psychoactive substance use | 27755 (10.46) | 2823 (9.36) | 24932 (10.60) | **0.87 (0.84-0.91)**** | **1.39 (1.31-1.48)**** |
| F20-F29:schizophrenia, schizotypal and delusional disorders | 18516 (6.98) | 2300 (7.62) | 16216 (6.90) | **1.11 (1.06-1.17)**** | **1.08 (1.01-1.15)*** |
| F30-F39: mood (affective) disorders | 42578 (16.05) | 5027 (16.66) | 37551 (15.97) | **1.05 (1.02-1.09)*** | **0.93 (0.88-0.97)*** |



For peer review only

| | | | | | |
|---|---|---|---|---|---|
| F40-F49: neurotic, stress-related and somatoform disorders | 33248 (12.53) | 2331 (7.73) | 30917 (13.15) | **0.55 (0.53-0.58)**** | **0.73 (0.69-0.78)**** |
| F50-F59: behavioural syndromes associated with physiological disturbances and physical factors | 8743 (3.30) | 148 (0.49) | 8595 (3.66) | **0.13 (0.11-0.15)**** | **0.47 (0.39-0.57)**** |
| F60-F69: disorders of adult personality and behaviour | 6945 (2.62) | 415 (1.38) | 6530 (2.78) | **0.49 (0.44-0.54)**** | 1.02 (0.91-1.15) |
| F70-F79: mental retardation | 3018 (1.14) | 311 (1.03) | 2707 (1.15) | 0.89 (0.79-1.01) | **1.24 (1.08-1.44)*** |
| F80-F89: disorders of psychological development | 7242 (2.73) | 54 (0.18) | 7188 (3.06) | **0.06 (0.04-0.07)**** | **0.65 (0.49-0.87)*** |
| F90-F98: behavioural and emotional disorders with onset usually occurring in childhood and adolescence | 17011 (6.41) | 77 (0.26) | 16934 (7.20) | **0.03 (0.03-0.04)**** | **0.45 (0.35-0.58)**** |
| Other diagnosis | 119638 (45.10) | 11014 (36.50) | 108624 (46.20) | **0.67 (0.65-0.69)**** | **0.65 (0.63-0.67)**** |
| Quartiles of face to face contacts: *n* (%) | | | | | |
| 1st (least face to face contact) | 76602 (28.87) | 5926 (19.64) | 70676 (30.06) | (reference) | (reference) |
| 2nd | 64914 (24.47) | 7767 (25.74) | 57147 (24.30) | **1.62 (1.56-1.68)**** | **1.10 (1.04-1.16)**** |
| 3rd | 59337 (22.37) | 8798 (29.16) | 50539 (21.49) | **2.08 (2.00-2.15)**** | **1.11 (1.05-1.17)**** |
| 4th (most face to face contact) | 64447 (24.29) | 7682 (25.46) | 56765 (24.14) | **1.61 (1.56-1.67)**** | 1.00 (0.95-1.06) |
| Inpatient bed days: *n* (%) | | | | | |
| None (0) | 238573 (89.93) | 26688 (88.45) | 211885 (90.12) | (reference) | (reference) |
| Low (1-2 days) | 1446 (0.55) | 102 (0.34) | 1344 (0.57) | **0.60 (0.49-0.74)**** | **1.52 (1.20-1.93)**** |
| Moderate (3-31 days) | 9825 (3.70) | 1034 (3.43) | 8791 (3.74) | **0.93 (0.87-1.00)*** | **1.58 (1.45-1.71)**** |
| High (32+ days) | 15456 (5.83) | 2349 (7.79) | 13107 (5.57) | **1.42 (1.36-1.49)**** | **1.59 (1.49-1.70)**** |

**Note.** **p*<0.05. ***p*<0.001. [1]adjusted for all other variables listed in the table. Missing data: age (*n*=275); sex (*n*=55); ethnicity (*n*=28,727); resident in local catchment area (*n*=9,537); quartiles of neighbourhood deprivation (*n*=11,702); referral status (*n*=989).



For peer review only - http://bmjopen.bmj.com/site/about/guidelines.xhtml

**Table 5.** Logistic regression analysis examining socio-demographic and clinical associations with linked HES-ONS mortality in the matched cohort.

| Variable | Total Population (N=248,698) | Patient Deceased (*n*=28,161 11.32%) | Patient Alive (*n*=220,537 88.68%) | OR (95% CI) | aOR[1] (95% CI) | aOR[2] (95% CI) |
|---|---|---|---|---|---|---|
| **Sociodemographic variables** | | | | | | |
| Age: mean (SD) | 43.50 (22.79) | 77.75 (18.75) | 39.13 (19.28) | **1.091 (1.090-1.092)**** | **1.093 (1.092-1.095)**** | **1.092 (1.091-1.094)**** |
| Male Sex: *n* (%) | 123818 (49.79) | 13824 (49.09) | 109994 (49.88) | **0.97 (0.95-0.99)*** | **1.51 (1.45-1.56)**** | **1.50 (1.45-1.55)**** |
| Ethnicity coded: *n* (%) | | | | | | |
| British, Irish, or any other white ethnic groups | 131150 (58.93) | 20717 (78.96) | 110433 (56.26) | (reference) | (reference) | (reference) |
| Mixed | 6477 (2.91) | 180 (0.69) | 6297 (3.21) | **0.15 (0.13-0.18)**** | **0.67 (0.56-0.80)**** | **0.65 (0.54-0.79)**** |
| Indian, Pakistani, Bangladeshi, or 'other Asian' | 10155 (4.56) | 915 (3.49) | 9240 (4.71) | **0.53 (0.49-0.57)**** | **0.68 (0.62-0.74)**** | **0.67 (0.62-0.73)**** |
| Caribbean, African, or any 'other black' | 38013 (17.08) | 2611 (9.95) | 35402 (18.03) | **0.39 (0.38-0.41)**** | **0.60 (0.57-0.64)**** | **0.60 (0.57-0.63)**** |
| Other | 15393 (6.92) | 626 (2.39) | 14767 (7.52) | **0.23 (0.21-0.25)**** | **0.50 (0.45-0.55)**** | **0.50 (0.46-0.55)**** |
| Not stated | 21354 (9.60) | 1189 (4.53) | 20165 (10.27) | **0.31 (0.30-0.33)**** | 0.94 (0.87-1.02) | **0.92 (0.86-1.00)*** |
| Resident in SLaM catchment area: *n* (%) | 177766 (73.53) | 22329 (81.48) | 155437 (72.51) | **1.67 (1.62-1.72)**** | **0.84 (0.80-0.88)**** | **0.84 (0.80-0.88)**** |
| Quartiles of neighbourhood deprivation: *n* (%) | | | | | | |
| 1st (most deprived) | 60220 (25.09) | 7217 (26.42) | 53003 (24.92) | (reference) | (reference) | (reference) |
| 2nd | 59786 (24.91) | 6573 (24.06) | 53213 (25.02) | **0.91 (0.88-0.94)**** | **1.08 (1.03-1.13)*** | **1.08 (1.03-1.13)*** |
| 3rd | 60052 (25.02) | 6857 (25.10) | 53195 (25.01) | **0.95 (0.91-0.98)*** | **1.23 (1.17-1.30)**** | **1.23 (1.17-1.29)**** |
| 4th (least deprived) | 59973 (24.99) | 6667 (24.41) | 53306 (25.06) | **0.92 (0.89-0.95)**** | **1.30 (1.23-1.36)**** | **1.29 (1.23-1.36)**** |
| **Clinical variables** | | | | | | |
| Primary diagnosis ever[a]: *n* (%) | | | | | | |
| F00-F09: organic, including symptomatic, mental disorders | 24248 (9.75) | 11940 (42.40) | 12308 (5.58) | **12.45 (12.09-12.83)**** | **1.05 (1.00-1.11)*** | **1.06 (1.01-1.11)*** |
| F10-F19: mental and behavioural disorders due to psychoactive substance use | 26158 (10.52) | 2549 (9.05) | 23609 (10.71) | **0.83 (0.80-0.87)**** | **1.35 (1.27-1.44)**** | **1.36 (1.28-1.45)**** |



For peer review only - http://bmjopen.bmj.com/site/about/guidelines.xhtml

| | | | | | | |
|---|---|---|---|---|---|---|
| F20-F29:schizophrenia, schizotypal and delusional disorders | 17323 (6.97) | 2081 (7.39) | 15242 (6.91) | 1.07 (1.02-1.13)* | 1.06 (0.99-1.14) | 1.05 (0.98-1.13) |
| F30-F39: mood (affective) disorders | 40515 (16.29) | 4715 (16.74) | 35800 (16.23) | 1.04 (1.00-1.07)* | 0.92 (0.88-0.97)** | 0.92 (0.88-0.97)* |
| F40-F49: neurotic, stress-related and somatoform disorders | 31599 (12.71) | 2187 (7.77) | 29412 (13.34) | 0.55 (0.52-0.57)** | 0.73 (0.68-0.77)** | 0.72 (0.68-0.77)** |
| F50-F59: behavioural syndromes associated with physiological disturbances and physical factors | 8388 (3.37) | 136 (0.48) | 8252 (3.74) | 0.12 (0.11-0.15)** | 0.47 (0.38-0.57)** | 0.48 (0.39-0.58)** |
| F60-F69: disorders of adult personality and behaviour | 6585 (2.65) | 368 (1.31) | 6217 (2.82) | 0.46 (0.41-0.51)** | 0.99 (0.88-1.13) | 0.98 (0.86-1.12) |
| F70-F79: mental retardation | 2898 (1.17) | 282 (1.00) | 2616 (1.19) | 0.84 (0.74-0.95)* | 1.17 (1.01-1.37)* | 1.17 (1.01-1.37)* |
| F80-F89: disorders of psychological development | 6991 (2.81) | 47 (0.17) | 6944 (3.15) | 0.05 (0.04-0.07)** | 0.60 (0.44-0.82)* | 0.61 (0.45-0.83)* |
| F90-F98: behavioural and emotional disorders with onset usually occurring in childhood and adolescence | 16363 (6.58) | 67 (0.24) | 16296 (7.39) | 0.03 (0.02-0.04)** | 0.42 (0.32-0.55)** | 0.43 (0.33-0.56)** |
| Other diagnosis | 112556 (45.26) | 10275 (36.49) | 102281 (46.38) | 0.66 (0.65-0.68)** | 0.64 (0.62-0.66)** | 0.64 (0.62-0.66)** |
| Quartiles of face to face contacts: *n* (%) | | | | | | |
| 1st (least face to face contact) | 70822 (28.48) | 5496 (19.52) | 65326 (29.62) | (reference) | (reference) | (reference) |
| 2nd | 59811 (24.05) | 7263 (25.79) | 52548 (23.83) | 1.64 (1.58-1.70)** | 1.09 (1.03-1.15)* | 1.09 (1.04-1.15)* |
| 3rd | 56116 (22.56) | 8282 (29.41) | 47834 (21.69) | 2.06 (1.99-2.13)** | 1.11 (1.05-1.17)** | 1.11 (1.05-1.18)** |
| 4th (most face to face contact) | 61949 (24.91) | 7120 (25.28) | 54829 (24.86) | 1.54 (1.49-1.60)** | 0.99 (0.93-1.05) | 1.00 (0.94-1.06) |
| Inpatient bed days: *n* (%) | | | | | | |
| None (0) | 223527 (89.88) | 24994 (88.75) | 198533 (90.02) | (reference) | (reference) | (reference) |
| Low (1-2 days) | 1342 (0.54) | 86 (0.31) | 1256 (0.57) | 0.54 (0.44-0.68)** | 1.40 (1.09-1.81)* | 1.36 (1.04-1.78)* |
| Moderate (3-31 days) | 9133 (3.67) | 939 (3.33) | 8194 (3.72) | 0.91 (0.85-0.98)* | 1.54 (1.41-1.68)** | 1.53 (1.40-1.67)** |
| High (32+ days) | 14696 (5.91) | 2142 (7.61) | 12554 (5.69) | 1.36 (1.29-1.42)** | 1.55 (1.45-1.66)** | 1.55 (1.44-1.66)** |
| Optimal Match | 203552 (84.15) | 178731 (87.81) | 24821 (12.19) | 0.63 (0.61-0.66)** | ----- | 0.85 (0.81-0.90)** |

**Note.** *$p$<0.05. **$p$<0.001. [1]adjusted for all other variables listed in the table (except optimal match). [2]adjusted for optimal match rank and inverse probability weighting for matching. Missing data: sex (*n*=37); ethnicity (*n*=26,156); resident in local catchment area (*n*=6,937); quartiles of neighbourhood deprivation (*n*=8,667); optimal match (*n*=6,808).



For peer review only - http://bmjopen.bmj.com/site/about/guidelines.xhtml

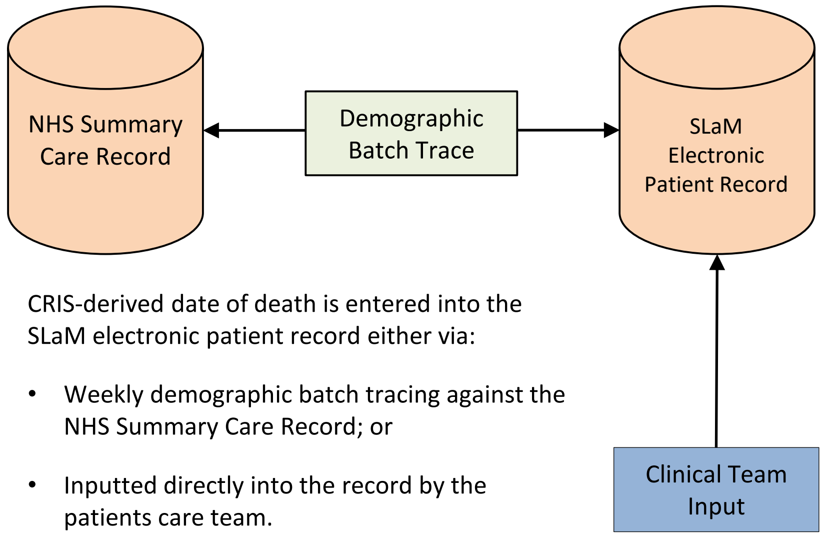
NHS Summary Care Record
Demographic Batch Trace
SLaM Electronic Patient Record
CRIS-derived date of death is entered into the SLaM electronic patient record either via:
• Weekly demographic batch tracing against the NHS Summary Care Record; or
• Inputted directly into the record by the patients care team.
Clinical Team Input

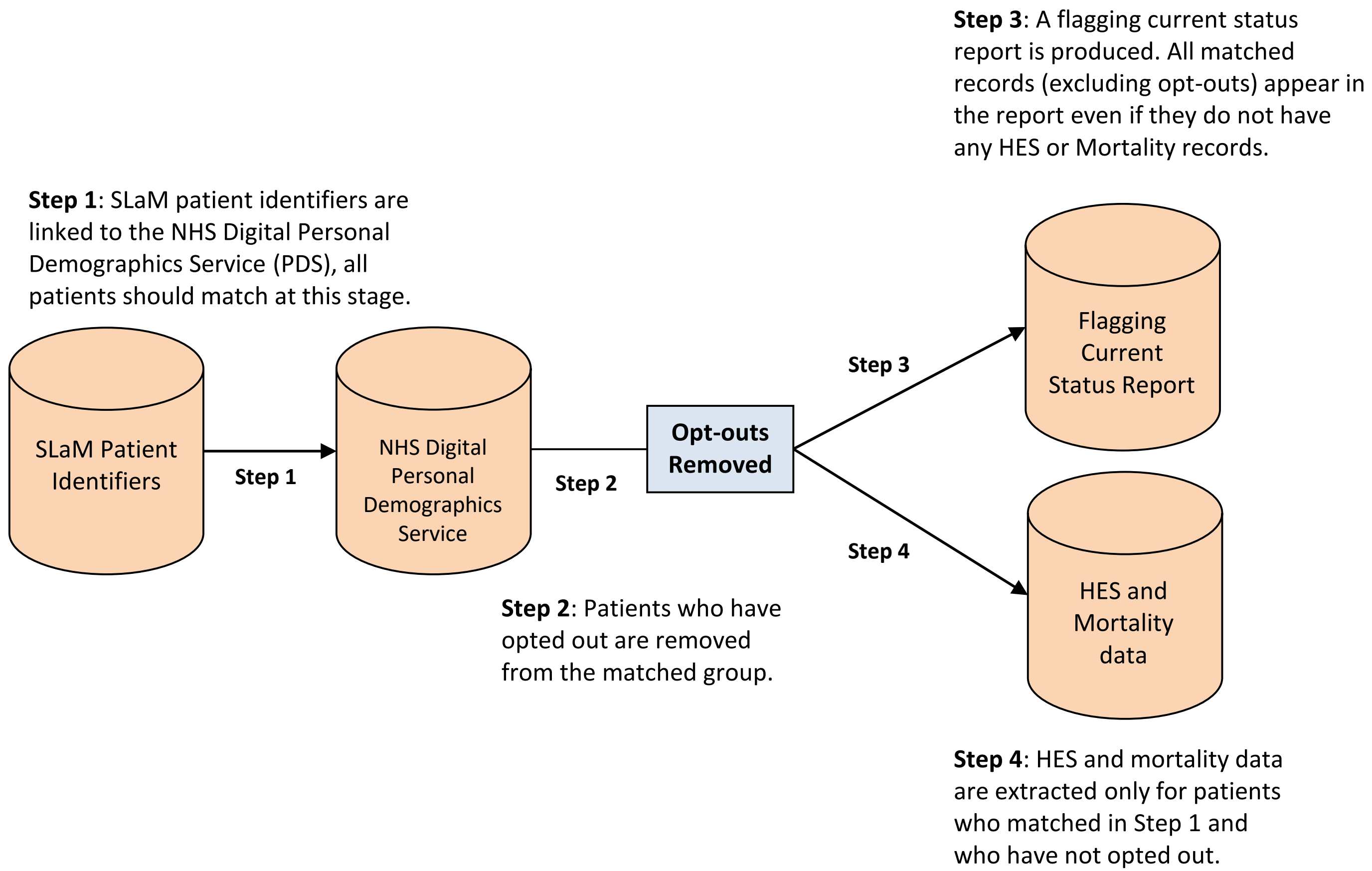
**Step 3**: A flagging current status report is produced. All matched records (excluding opt-outs) appear in the report even if they do not have any HES or Mortality records.
**Step 1**: SLaM patient identifiers are linked to the NHS Digital Personal Demographics Service (PDS), all patients should match at this stage.
SLaM Patient Identifiers
Step 1
NHS Digital Personal Demographics Service
Step 2
Opt-outs Removed
Step 3
Flagging Current Status Report
Step 4
HES and Mortality data
**Step 2**: Patients who have opted out are removed from the matched group.
**Step 4**: HES and mortality data are extracted only for patients who matched in Step 1 and who have not opted out.

**Table 1.** STROBE 2007 (v4) Statement: The Impact of Matching Error on Linked Mortality Outcome in a Data Linkage of Secondary Mental Health Data with Hospital Episode Statistics (HES) and Mortality Records in South East London: A Cross-Sectional Study

| Section/Topic | Item # | Recommendation | Reported on page # |
|---|---|---|---|
| **Title and abstract** | 1 | (*a*) Indicate the study's design with a commonly used term in the title or the abstract | 1 |
| | | (*b*) Provide in the abstract an informative and balanced summary of what was done and what was found | 2 |
| **Introduction** | | | |
| Background/rationale | 2 | Explain the scientific background and rationale for the investigation being reported | 4 |
| Objectives | 3 | State specific objectives, including any prespecified hypotheses | 4/5 |
| **Methods** | | | |
| Study design | 4 | Present key elements of study design early in the paper | 5 |
| Setting | 5 | Describe the setting, locations, and relevant dates, including periods of recruitment, exposure, follow-up, and data collection | 5/6 |
| Participants | 6 | (*a*) Give the eligibility criteria, and the sources and methods of selection of participants | 5 |
| Variables | 7 | Clearly define all outcomes, exposures, predictors, potential confounders, and effect modifiers. Give diagnostic criteria, if applicable | 5/24 |
| Data sources/ measurement | 8* | For each variable of interest, give sources of data and details of methods of assessment (measurement). Describe comparability of assessment methods if there is more than one group | 5/6 |
| Bias | 9 | Describe any efforts to address potential sources of bias | n/a |
| Study size | 10 | Explain how the study size was arrived at | 5 |
| Quantitative variables | 11 | Explain how quantitative variables were handled in the analyses. If applicable, describe which groupings were chosen and why | 24 |
| Statistical methods | 12 | (*a*) Describe all statistical methods, including those used to control for confounding | 7 |
| | | (*b*) Describe any methods used to examine subgroups and interactions | 7 |
| | | (*c*) Explain how missing data were addressed | n/a |
| | | (*d*) If applicable, describe analytical methods taking account of sampling strategy | n/a |
| | | (*e*) Describe any sensitivity analyses | 7 |

For peer review only

| | | | |
|---|---|---|---|
| **Results** | | | |
| Participants | 13* | (a) Report numbers of individuals at each stage of study—eg numbers potentially eligible, examined for eligibility, confirmed eligible, included in the study, completing follow-up, and analysed | 8 |
| | | (b) Give reasons for non-participation at each stage | n/a |
| | | (c) Consider use of a flow diagram | n/a |
| Descriptive data | 14* | (a) Give characteristics of study participants (eg demographic, clinical, social) and information on exposures and potential confounders | 8 |
| | | (b) Indicate number of participants with missing data for each variable of interest | 28/30/32 |
| Outcome data | 15* | Report numbers of outcome events or summary measures | 8/9/10 |
| Main results | 16 | (*a*) Give unadjusted estimates and, if applicable, confounder-adjusted estimates and their precision (eg, 95% confidence interval). Make clear which confounders were adjusted for and why they were included | 8/9/10/28/30/32 |
| | | (*b*) Report category boundaries when continuous variables were categorized | n/a |
| | | (*c*) If relevant, consider translating estimates of relative risk into absolute risk for a meaningful time period | n/a |
| Other analyses | 17 | Report other analyses done—eg analyses of subgroups and interactions, and sensitivity analyses | 8/9/10/11 |
| **Discussion** | | | |
| Key results | 18 | Summarise key results with reference to study objectives | 11/12 |
| Limitations | 19 | Discuss limitations of the study, taking into account sources of potential bias or imprecision. Discuss both direction and magnitude of any potential bias | 13/14 |
| Interpretation | 20 | Give a cautious overall interpretation of results considering objectives, limitations, multiplicity of analyses, results from similar studies, and other relevant evidence | 11/12 |
| Generalisability | 21 | Discuss the generalisability (external validity) of the study results | 13/14 |
| **Other information** | | | |
| Funding | 22 | Give the source of funding and the role of the funders for the present study and, if applicable, for the original study on which the present article is based | 15 |

*Give information separately for cases and controls in case-control studies and, if applicable, for exposed and unexposed groups in cohort and cross-sectional studies.

For peer review only

**Table 2.** Logistic regression analysis examining administrative, socio-demographic, and clinical associations with an optimal match.

| Variable | Total Population (N=241,890) | Optimal Match (*n*=203,552 84.15%) | Non-optimal Match (*n*=38,338 15.85%) | OR (95% CI) | aOR[1] (95% CI) |
|---|---|---|---|---|---|
| **Sociodemographic variables** | | | | | |
| Age: mean (SD) | 43.70 (22.89) | 44.68 (23.01) | 38.47 (21.52) | **0.987 (0.987-0.988)**** | **0.985 (0.984-0.986)**** |
| Male Sex: *n* (%) | 119634 (49.46) | 99187 (48.73) | 20447 (53.38) | **1.20 (1.18-1.23)**** | **1.14 (1.12-1.17)**** |
| Patient deceased: *n* (%) | 28790 (11.90) | 25629 (12.59) | 3161 (8.25) | **0.63 (0.61-0.65)**** | 0.95 (0.91-1.00) |
| Ethnicity coded: *n* (%) | | | | | |
| British, Irish, or any other white ethnic groups | 128547 (59.32) | 109139 (59.71) | 19408 (57.25) | (reference) | (reference) |
| Mixed | 6306 (2.91) | 5168 (2.83) | 1138 (3.36) | **1.24 (1.16-1.32)**** | 1.00 (0.93-1.07) |
| Indian, Pakistani, Bangladeshi, or 'other Asian' | 9745 (4.50) | 8348 (4.57) | 1397 (4.12) | **0.94 (0.89-0.99)*** | **0.90 (0.85-0.96)*** |
| Caribbean, African, or any 'other black' | 37052 (17.10) | 31115 (17.02) | 5937 (17.51) | **1.07 (1.04-1.11)**** | 0.97 (0.93-1.00) |
| Other | 14728 (6.80) | 12248 (6.70) | 2480 (7.32) | **1.14 (1.09-1.19)**** | **1.08 (1.03-1.13)*** |
| Not stated | 20319 (9.38) | 16778 (9.18) | 3541 (10.45) | **1.19 (1.14-1.23)**** | **1.04 (1.00-1.09)*** |
| Resident in SLaM catchment area: *n* (%) | 172567 (73.34) | 145594 (73.34) | 26973 (73.37) | 1.00 (0.98-1.03) | **1.13 (1.09-1.16)**** |
| Quartiles of neighbourhood deprivation: *n* (%) | | | | | |
| 1st (most deprived) | 58789 (25.15) | 49830 (25.24) | 8959 (24.67) | (reference) | (reference) |
| 2nd | 58146 (24.87) | 48785 (24.71) | 9361 (25.78) | **1.07 (1.03-1.10)**** | 1.01 (0.98-1.05) |
| 3rd | 58342 (24.96) | 49200 (24.92) | 9142 (25.18) | **1.03 (1.00-1.07)*** | 0.97 (0.94-1.01) |
| 4th (least deprived) | 58488 (25.02) | 49638 (25.14) | 8850 (24.37) | 0.99 (0.96-1.02) | **0.92 (0.89-0.96)**** |
| **Clinical variables** | | | | | |
| Referral status in past 2 years: *n* (%) | | | | | |
| Accepted | 11061 (4.59) | 9445 (4.65) | 1616 (4.23) | (reference) | (reference) |
| Discharged | 41227 (17.09) | 35007 (17.25) | 6220 (16.27) | 1.04 (0.98-1.10) | **1.11 (1.04-1.18)*** |
| Rejected | 8559 (3.55) | 7060 (3.48) | 1499 (3.92) | **1.24 (1.06-1.18)**** | **1.10 (1.01-1.21)*** |
| No referral in 2 year prior to linkage | 180330 (74.77) | 151442 (74.62) | 28888 (75.58) | **1.11 (1.06-1.18)**** | **1.24 (1.17-1.32)**** |
| Primary diagnosis ever: *n (%)* | | | | | |
| F00-F09: organic, including symptomatic, mental disorders | 24095 (9.96) | 21191 (10.41) | 2904 (7.57) | **0.71 (0.68-0.73)**** | **1.31 (1.24-1.39)**** |
| F10-F19: mental and behavioural disorders due to psychoactive substance use | 25530 (10.55) | 21124 (10.38) | 4406 (11.49) | **1.12 (1.08-1.16)**** | **1.17 (1.12-1.22)**** |
| F20-F29:schizophrenia, schizotypal and delusional disorders | 16889 (6.98) | 14328 (7.04) | 2561 (6.68) | **0.95 (0.91-0.99)*** | 1.05 (0.99-1.11) |

For peer review only

| | | | | | |
|---|---|---|---|---|---|
| F30-F39: mood (affective) disorders | 39799 (16.45) | 34512 (16.95) | 5287 (13.79) | **0.78 (0.76-0.81)**** | **0.88 (0.85-0.92)**** |
| F40-F49: neurotic, stress-related and somatoform disorders | 30943 (12.79) | 26729 (13.13) | 4214 (10.99) | **0.82 (0.79-0.85)**** | **0.83 (0.79-0.86)**** |
| F50-F59: behavioural syndromes associated with physiological disturbances and physical factors | 8086 (3.34) | 6634 (3.26) | 1452 (3.79) | **1.17 (1.10-1.24)**** | **1.12 (1.05-1.19)*** |
| F60-F69: disorders of adult personality and behaviour | 6509 (2.69) | 5521 (2.71) | 988 (2.58) | 0.95 (0.89-1.02) | 0.98 (0.91-1.05) |
| F70-F79: mental retardation | 2810 (1.16) | 2448 (1.20) | 362 (0.94) | **0.78 (0.70-0.87)**** | **0.82 (0.73-0.92)**** |
| F80-F89: disorders of psychological development | 6804 (2.81) | 5673 (2.79) | 1131 (2.95) | 1.06 (0.99-1.13) | **0.76 (0.70-0.81)**** |
| F90-F98: behavioural and emotional disorders with onset usually occurring in childhood and adolescence | 15931 (6.59) | 12877 (6.33) | 3054 (7.97) | **1.28 (1.23-1.34)**** | 0.96 (0.91-1.00) |
| Other diagnosis | 109854 (45.41) | 92623 (45.50) | 17231 (44.94) | **0.98 (0.96-1.00)*** | **0.96 (0.94-0.99)**** |
| Quartiles of face to face contacts: *n* (%) | | | | | |
| 1st (least face to face contact) | 68150 (28.17) | 56507 (27.76) | 11643 (30.37) | (reference) | (reference) |
| 2nd | 58128 (24.03) | 48702 (23.93) | 9426 (24.59) | **0.94 (0.91-0.97)**** | 1.01 (0.97-1.04) |
| 3rd | 54781 (22.65) | 46311 (22.75) | 8470 (22.09) | **0.89 (0.86-0.92)**** | **0.95 (0.91-0.99)*** |
| 4th (most face to face contact) | 60831 (25.15) | 52032 (25.56) | 8799 (22.95) | **0.82 (0.80-0.85)**** | **0.89 (0.85-0.93)**** |
| Inpatient bed days: *n* (%) | | | | | |
| None (0) | 216890 (89.66) | 182362 (89.59) | 34528 (90.06) | (reference) | (reference) |
| Low (1-2 days) | 1327 (0.55) | 1106 (0.54) | 221 (0.58) | 1.06 (0.91-1.22) | 1.06 (0.91-1.24) |
| Moderate (3-31 days) | 9064 (3.75) | 7607 (3.74) | 1457 (3.80) | 1.01 (0.96-1.07) | 1.05 (0.98-1.12) |
| High (32+ days) | 14609 (6.04) | 12477 (6.13) | 2132 (5.56) | **0.90 (0.86-0.95)**** | 1.00 (0.95-1.06) |

**Note.** Optimal match data missing for *n*=6,808 (2.75%) matched records. **p*<0.05. ***p*<0.001. [1]adjusted for all other variables listed in the table. Missing data: sex (*n*=34); ethnicity (*n*=25,193); resident in local catchment area (*n*=6,604); quartiles of neighbourhood deprivation (*n*=8,125); referral status (*n*=713).

For peer review only

**Table 3.** Logistic regression analysis examining the association between sociodemographic factors and availability of NHS number for matching.

| Variable | Total Population (N=265,300) | NHS Number Present (*n*=259,545 97.83%) | NHS Number Missing (*n*=5,755 2.17%) | OR (95% CI) | aOR[1] (95% CI) |
|---|---|---|---|---|---|
| **Sociodemographic variables** | | | | | |
| Age: mean (SD) | 43.40 (22.69) | 43.49 (22.79) | 39.19 (16.57) | **1.009 (1.008-1.010)**** | **1.010 (1.008-1.012)**** |
| Male Sex: *n* (%) | 132730 (50.04) | 126085 (49.74) | 3645 (63.47) | **0.57 (0.54-0.60)**** | **0.60 (0.56-0.66)**** |
| Ethnicity: *n* (%) | | | | | |
| British, Irish, or any other white ethnic groups | 138495(58.54) | 136495 (58.69) | 2265 (50.99) | (reference) | (reference) |
| Mixed | 6853 (2.90) | 6782 (2.92) | 71 (1.60) | **1.59 (1.25-2.01)**** | **1.60 (1.16-2.19)*** |
| Indian, Pakistani, Bangladeshi, or 'other Asian' | 10889 (4.60) | 10586 (4.56) | 303 (6.82) | **0.58 (0.51-0.66)**** | **0.44 (0.38-0.51)**** |
| Caribbean, African, or 'other black' | 40725 (17.21) | 40008 (17.24) | 717 (16.14) | 0.93 (0.85-1.01) | **0.73 (0.65-0.82)**** |
| Other | 16650 (7.04) | 16120 (6.94) | 530 (11.93) | **0.51 (0.46-0.56)**** | **0.45 (0.39-0.51)**** |
| Not stated | 22961 (9.71) | 22405 (9.65 | 556 (12.52) | **0.67 (0.61-0.74)**** | **0.58 (0.51-0.65)**** |
| Resident in SLaM Catchment Area: *n* (%) | 187773 (73.42) | 185393 (73.51) | 2380 (66.93) | **1.37 (1.28-1.47)**** | **1.15 (1.04-1.26)*** |
| Quartiles of neighbourhood deprivation: *n* (%) | | | | | |
| 1st (most deprived) | 63476 (25.03) | 62832 (25.10) | 644 (19.93) | (reference) | (reference) |
| 2nd | 63452 (25.02) | 62605 (25.01) | 847 (26.21) | **0.76 (0.68-0.84)**** | **0.66 (0.59-0.75)**** |
| 3rd | 63449 (25.02) | 62557 (24.99) | 892 (27.60) | **0.72 (0.65-0.80)**** | **0.68 (0.60-0.77)**** |
| 4th (least deprived) | 63221 (24.93 | 62371 (24.91) | 849 (26.27) | **0.75 (0.68-0.83)**** | **0.70 (0.61-0.79)**** |

**Note.** *$p$<0.05. **$p$<0.001. [1]adjusted for all other variables listed in the table. Missing data: age (*n*=275); sex (*n*=55); ethnicity (*n*=28,727); quartiles of neighbourhood deprivation (*n*=11,702).

**Table 4.** Logistic regression analysis examining the association between sociodemographic factors and availability of date of birth for matching.

| Variable | Total Population (N=265,300) | Date of Birth Present (*n*=265,010 99.89%) | Date of Birth Missing (*n*=290 0.11%) | OR (95% CI) | aOR[1] (95% CI) |
|---|---|---|---|---|---|
| **Sociodemographic variables** | | | | | |
| Age: mean (SD) | 43.40 (22.69) | 43.40 (22.69) | 32.93 (20.49) | 1.025 (0.997-1.054) | 1.033 (0.974-1.096) |
| Male Sex: *n* (%) | 132730 (50.04) | 132585 (50.04) | 145 (50.17) | 0.99 (0.79-1.25) | 1.78 (0.29-10.77) |
| Ethnicity: *n* (%) | | | | | |
| British, Irish, or any other white ethnic groups | 138495(58.54) | 138440 (58.55) | 55 (50.00) | (reference) | (reference) |
| Mixed | 6853 (2.90) | 6853 (2.90) | 0 (0.00) | ---- | ---- |
| Indian, Pakistani, Bangladeshi, or 'other Asian' | 10889 (4.60) | 10877 (4.60) | 12 (10.91) | **0.36 (0.19-0.67)*** | 0.08 (0.01-1.22) |
| Caribbean, African, or 'other black' | 40725 (17.21) | 40710 (17.22) | 15 (13.64) | 1.08 (0.61-1.91) | ---- |
| Other | 16650 (7.04) | 16632 (7.03) | 18 (16.36) | **0.37 (0.22-0.63)**** | 0.10 (0.01-1.73) |
| Not stated | 22961 (9.71) | 22951 (9.71) | 10 (9.09) | 0.91 (046-1.79) | 0.11 (0.01-1.23) |
| Resident in SLaM Catchment Area: *n* (%) | 187773(73.42) | 187661 (73.42) | 112 (73.68) | 0.99 (0.69-1.42) | 3.23 (0.46-22.53) |
| Quartiles of neighbourhood deprivation: *n* (%) | | | | | |
| 1st (most deprived) | 63476 (25.03) | 63428 (25.03) | 48 (33.33) | (reference) | (reference) |
| 2nd | 63452 (25.02) | 63427 (25.03) | 25 (17.36) | **1.92 (1.18-3.11)*** | 1.43 (0.12-16.75) |
| 3rd | 63449 (25.02) | 63405 (25.02) | 44 (30.56) | 1.09 (0.72-1.64) | 1.26 (0.10-15.36) |
| 4th (least deprived) | 63221 (24.93 | 63194 (24.93) | 27 (18.75) | **1.77 (1.11-2.84)*** | 1.23 (0.10-15.00) |

**Note.** **p*<0.05. ***p*<0.001. [1]adjusted for all other variables listed in the table. Missing data: age (*n*=275); sex (*n*=55); ethnicity (*n*=28,727); quartiles of neighbourhood deprivation (*n*=11,702).

For peer review only

BMJ Open

# The Impact of Matching Error on Linked Mortality Outcome in a Data Linkage of Secondary Mental Health Data with Hospital Episode Statistics (HES) and Mortality Records in South East London: A Cross-Sectional Study



| | |
|---|---|
| Journal: | *BMJ Open* |
| Manuscript ID | bmjopen-2019-035884.R2 |
| Article Type: | Original research |
| Date Submitted by the Author: | 20-May-2020 |



| | |
|---|---|
| Complete List of Authors: | Jewell, Amelia; South London and Maudsley NHS Foundation Trust, Maudsley Biomedical Research Centre (BRC)<br>Broadbent, Matthew; South London and Maudsley NHS Foundation Trust, Maudsley Biomedical Research Centre (BRC)<br>Hayes, Richard; Institute of Psychiatry, Kings College London, Psychological Medicine<br>Gilbert, Ruth; UCL Institute of Child Health, Centre for Paediatric Epidemiology and Biostatistics<br>Stewart, Robert; King's College London, Institute of Psychiatry<br>Downs, Johnny; Kings College London, Institute of Psychiatry, Psychology and Neuroscience |



| | |
|---|---|
| <b>Primary Subject Heading</b>: | Health informatics |
| Secondary Subject Heading: | Mental health |
| Keywords: | Health informatics < BIOTECHNOLOGY & BIOINFORMATICS, MENTAL HEALTH, PSYCHIATRY |
| | |


SCHOLARONE™
Manuscripts

*I, the Submitting Author has the right to grant and does grant on behalf of all authors of the Work (as defined in the below author licence), an exclusive licence and/or a non-exclusive licence for contributions from authors who are: i) UK Crown employees; ii) where BMJ has agreed a CC-BY licence shall apply, and/or iii) in accordance with the terms applicable for US Federal Government officers or employees acting as part of their official duties; on a worldwide, perpetual, irrevocable, royalty-free basis to BMJ Publishing Group Ltd ("BMJ") its licensees and where the relevant Journal is co-owned by BMJ to the co-owners of the Journal, to publish the Work in this journal and any other BMJ products and to exploit all rights, as set out in our licence.*

*The Submitting Author accepts and understands that any supply made under these terms is made by BMJ to the Submitting Author unless you are acting as an employee on behalf of your employer or a postgraduate student of an affiliated institution which is paying any applicable article publishing charge ("APC") for Open Access articles. Where the Submitting Author wishes to make the Work available on an Open Access basis (and intends to pay the relevant APC), the terms of reuse of such Open Access shall be governed by a Creative Commons licence – details of these licences and which Creative Commons licence will apply to this Work are set out in our licence referred to above.*

*Other than as permitted in any relevant BMJ Author's Self Archiving Policies, I confirm this Work has not been accepted for publication elsewhere, is not being considered for publication elsewhere and does not duplicate material already published. I confirm all authors consent to publication of this Work and authorise the granting of this licence.*

For peer review only

**The Impact of Matching Error on Linked Mortality Outcome in a Data Linkage of Secondary Mental Health Data with Hospital Episode Statistics (HES) and Mortality Records in South East London: A Cross-Sectional Study**

Amelia Jewell[1*], Matthew Broadbent[1], Richard D Hayes[2], Ruth Gilbert[3], Robert Stewart[1,2], and Johnny Downs[1,2]

* Corresponding author contact information:

Amelia Jewell, Biomedical Research Centre (BRC) Nucleus (PO92), Kings College London, DeCrespigny Park, London, SE5 8AF, United Kingdom. Telephone: +44 20 3228 8554; E-mail: amelia.jewell@slam.nhs.uk

**Author details:**

[1] South London and Maudsley NHS Foundation Trust, London, UK. [2] Institute of Psychiatry, Psychology and Neuroscience, King's College London, London, UK. [3] University College London Great Ormond Street Institute of Child Health, London, UK.

For peer review only



For peer review only - http://bmjopen.bmj.com/site/about/guidelines.xhtml

**ABSTRACT**

**Objectives:** Linkage of electronic health records (EHRs) to Hospital Episode Statistics (HES)-Office for National Statistics (ONS) mortality data has provided compelling evidence for lower life expectancy in people with severe mental illness. However, linkage error may underestimate these estimates. Using a clinical sample (N=265,300) of individuals accessing mental health services, we examined potential biases introduced through missed matching, and examined the impact on the association between clinical disorders and mortality.

**Setting:** The South London and Maudsley NHS Foundation Trust (SLaM) is a secondary mental health care provider in London. A de-identified version of SLaM's EHR was available via the Clinical Record Interactive Search (CRIS) system linked to HES-ONS mortality records.

**Participants:** Records from SLaM for patients active between January 2006 and December 2016.

**Outcome measures:** Two sources of death data were available for SLaM participants: accurate and contemporaneous date of death via local batch tracing (gold standard), and date of death via linked HES-ONS mortality data. The effect of linkage error on mortality estimates was evaluated by comparing sociodemographic and clinical risk factor analyses using gold standard death data, against HES-ONS mortality records.

**Results:** Of the total sample, 93.74% were successfully matched to HES-ONS records. We found a number of statistically significant administrative, socio-demographic, and clinical differences between matched and unmatched records. Of note, schizophrenia diagnosis showed a significant association with higher mortality using gold standard data (OR=1.08; 95% CI=1.01, 1.15; *p*=0.02), but not in HES-ONS data (OR=1.05; 95% CI=0.98, 1.13; *p*=0.16). Otherwise little change was found in the strength of associated risk factors and mortality, after accounting for missed matching bias.

**Conclusions:** Despite significant clinical and socio-demographic differences between matched and unmatched records, changes in mortality estimates were minimal. However, researchers and policy analysts using HES-ONS linked resources should be aware that administrative linkage processes can introduce error.



For peer review only - http://bmjopen.bmj.com/site/about/guidelines.xhtml

**Strengths and limitations of this study:**

- The findings of our study demonstrate that although there are significant differences between matched and unmatched records, these did not significantly change the relative differences in mortality according to type of psychiatric disorder.
- The findings from this study are novel, in that data linkage error has not previously been evaluated in a routine mental health database and this may be useful for other researchers looking to conduct research using electronic health records linked to HES-ONS mortality data by NHS Digital.
- A significant strength of the study is our access to the gold standard death data within the mental health record, which can be used to compare with the linked mortality data; this is useful as the mental health discrepancy in mortality is a particular focus on issues of parity/ inequality for health policy.
- Results may be generalisable to other NHS cohorts being linked to HES-ONS mortality data via NHS Digital as national standard matching methodology was used for the data linkage and the quality and type of administrative data available for matching is likely to be similar within most NHS Trusts.
- False matches were not examined; therefore, total data linkage error within the linked dataset may be higher.

For peer review only



For peer review only - http://bmjopen.bmj.com/site/about/guidelines.xhtml

## INTRODUCTION

Individuals with mental health disorders have substantially lower life expectancy in comparison to those without mental health disorders [1,2]. The United Kingdom (UK) government has been working to reduce the mortality gap between those with and without mental health disorders by implementing policies aimed at improving the physical health of individuals with mental illness [3]. To inform changes in health policy it is essential that we have good data available on health (physical and mental) and mortality in order to understand the trends and underlying mechanisms.

Electronic health record (EHR) data from the South London and Maudsley NHS Foundation Trust (SLaM) Clinical Record Interactive Search (CRIS) system [4] linked to Hospital Episode Statistics (HES) and death certificate data from the Office for National Statistics (ONS) have previously been utilised to provide evidence of the mental health mortality gap and investigate potential underlying mechanisms [5-9]. Data linkages such as that between CRIS and HES-ONS mortality resources are an important tool for increasing the use of existing data resources to support research [10]. However, the success of any data linkage and the quality of the subsequent linked dataset rely on multiple factors, including the data quality and linkage methodology. The two main types of linkage error which can occur during the data linkage process are false matches (i.e. false positives) and missed matches (i.e. false negatives). Errors in data linkage may result in systematic bias in reported outcomes [11]. False positives in data linkages can dilute the association between variables [12] whereas false negatives can cause an underestimation of the risk outcome [13]; for example, a US study found that linking on an infant's medical insurance record, as opposed to the mother's, led to a significant underestimation in infant mortality due to the number of missed matches [14].

There are a number of approaches to evaluating linkage quality; for example by comparing characteristics of linked and unlinked records to identify potential sources of bias [12]. Previous studies have identified significant differences in socio-demographic characteristics between matched and unmatched samples in data linkages, including sex [15-21], age [15,19,22-27], ethnicity [16,19,20-22,26-32], socioeconomic status [20,27,33,34], marital status [15,22,28,35], and level of education [20,34,35], providing some indication that linked data may not always be representative of the population of interest [12]. Reference or 'gold standard' datasets in which the true match status is known can be used to evaluate the impact of data linkage error by quantifying missed or false matches [36]; however, gold standard data either do not exist or are rarely available to researchers using linked data due to issues around governance and data protection [12].

If linked data are being used to inform health policy it is important that the effect of linkage error is estimated. At present, for example, it is not clear whether any potential error in the linkage between CRIS and HES-ONS mortality data has a bearing on the research being conducted using the linked data. It was therefore our aim to investigate the effect of linkage error on the prediction of a



For peer review only - http://bmjopen.bmj.com/site/about/guidelines.xhtml

linked outcome measure in this mental health population by, firstly, examining the administrative, socio-demographic, and clinical characteristics of matched and unmatched records, and secondly, by comparing the prediction of linked mortality outcome with gold-standard death data provided by the local mental health record.

## METHODS

*The Mental Health Dataset - CRIS*

The Maudsley Biomedical Research Centre (BRC) CRIS system, described previously in detail [4,37], is a deidentified database of electronic medical records for mental health service users within SLaM: one of the largest providers of secondary mental health care in Europe. SLaM provides local area mental health services predominately for the London boroughs of Lambeth, Southwark, Croydon, and Lewisham (the SLaM 'catchment area'), as well as some national specialist services [4]. Records from CRIS have previously been linked to a number of external databases including the National Pupil Database [27,38], the Thames Cancer Registry [39], and General Practice (GP) data [40].

All individuals (children and adults) who were active in SLaM between 1st January 2006 and 20th December 2016, not including IAPT (Improving Access to Psychological Therapies) services, were included in the current study. Exposure variables (Table 1) were collected from structured fields within the CRIS system and included socio-demographic (e.g. age, sex, ethnicity, and mortality), clinical (e.g. mental health diagnosis, referral status, level of service use), and administrative variables (i.e. patient identifiers available for linkage).

Mortality data from CRIS provided the 'gold standard' death data using both formal and informal notification sources [41]. All-cause mortality data is entered into the SLaM EHR either by the care team directly (for example after being informed by family members) or via automatic tracing, whereby the SLaM Trust run a demographic batch trace weekly for all patients (past or current) against the NHS Summary Care Record (SCR). This returns date of death for those patients who have died (Figure 1). The SCR is an electronic record of important patient information created from GP medical records which can be seen and used by any authorised staff in areas of the health system who are involved in a patients' direct care. The SCR is administered by NHS Digital [42]. Date of death in the SCR is entered at the point of occurrence, i.e. the death may not have been formally registered yet.

*The Hospital Episode Statistics and ONS Mortality Dataset*

HES data are a national dataset, governed by NHS Digital, which holds details of all admissions, outpatient appointments, and accident and emergency attendances at NHS hospitals in England [43]. HES data are derived from the Secondary Uses Service (SUS) database which collects submissions routinely from all NHS acute hospital and mental health trusts in England. The data accuracy and



For peer review only - http://bmjopen.bmj.com/site/about/guidelines.xhtml

quality within these submissions are important, especially to NHS Trusts, as they are used to calculate payments for the care provided [44]. HES data are designed to enable secondary use, e.g. for research, and can be linked to other external data sources.

The ONS mortality data consists of the Primary Care Mortality Database (PCMD) which includes date and cause of death for all deaths registered in England and Wales. The data are collected by the ONS and access to the data is managed by NHS Digital [45]. Mortality data from the PCMD are only returned for individuals who have matched and have died; individuals without a mortality record are therefore assumed to be alive.

*Linkage Procedures*

The linkage between CRIS, HES-ONS mortality data under analysis here was conducted in January 2017. Patient Identifiable Information (PII), i.e. date of birth, sex, NHS number, and postcode, for all SLaM records (excluding anybody who had previously opted out of CRIS) was extracted on 20th December 2016. The data linkage was conducted by NHS Digital following deterministic matching procedures. Date of birth, sex, NHS number, and most recently recorded postcode were used as the personal identifiers to match records. Table 2 demonstrates the match ranks used by NHS Digital, with the quality of the matching decreasing from step one to eight.

Initially records are matched to the Personal Demographics Service (PDS) using this method. The PDS is the national electronic database of NHS patient details such as name, address, date of birth, and NHS number [46]. The PDS is also involved in the creation and updating of the NHS SCR, described above. Theoretically, every CRIS record should match to the PDS, even if they do not have any HES episodes or a mortality record. Following matching to the PDS, patients who have opted out of their data being used for secondary purposes, known as 'type 2' opt-outs [47], are removed from the cohort. A report is produced containing details of all the matched records, excluding unmatched records and opt-outs, known as the Flagging Current Status report. Mortality and HES data are then extracted for matched records only (see Figure 2).

Match status outcome measure (i.e. matched vs. missed match) was determined using the Flagging Current Status report following the linkage of CRIS records by NHS Digital. Unmatched records included both records which were not matched (i.e. missed matches) as well as opt-outs.

For those records which were successfully matched, we examined optimal match rank, whereby an optimal match represents a 'perfect' match or a match rank of one (see Table 2), i.e. the records matched on all supplied patient identifiers. The optimal match variable was provided by NHS Digital within the HES data following completion of the data linkage.

*Patient and Public Involvement*



For peer review only - http://bmjopen.bmj.com/site/about/guidelines.xhtml

Patient and public involvement was key to the initial development and ongoing oversight of the CRIS system. The data linkage between CRIS and the HES-ONS mortality data, as well as this project specifically, were approved by the service-user chaired CRIS Oversight Committee (project reference: 17-065). The CRIS, HES-ONS mortality data linkage was also presented to the Data Linkage Service User and Carer Advisory Group [48], a regular meeting of people with lived experience of mental illness, all of whom have an interest in mental health research involving data linkage. The group provide ongoing advice and feedback to researchers conducting projects using the linked CRIS and HES-ONS mortality data.

*Statistical Analysis*

*Step 1: Missed Matches Analysis*

Data were analysed using STATA version 15. Univariable logistic regression was first performed on all administrative, socio-demographic, and clinical characteristics to examine the association with match status outcome for HES-ONS linkage (i.e. those with a positive match on the Flagging Current Status report). Multivariable logistic regression was then performed to identify factors which remained significant predictors of HES-ONS matching after controlling for all other examined variables.

*Step 2: Optimal Match Analysis*

In order to determine which variables predicted an optimal match, univariable logistic regression analysis was performed on a subsample of the total cohort (i.e. only those records which were matched with HES-ONS). Multivariable logistic regression was then performed to identify factors associated with an optimal match outcome after accounting for all other examined variables.

*Step 3: CRIS Derived Mortality Analysis*

In order to examine the effect of potential matching bias on mortality outcome we first examined factors associated with all-cause mortality using the gold standard CRIS-derived death data in univariable and multivariable analyses.

*Step 4: Linked Mortality Analysis*

We then examined predictors of mortality using the linked mortality outcome (i.e. patients with a death date in the HES-ONS matched dataset), in the HES-ONS matched group only, in univariable and multivariable analyses. We used inverse probability weighting calculated from the Step 1 analysis and the optimal match variable to adjust the final model for missed matching bias.



For peer review only - http://bmjopen.bmj.com/site/about/guidelines.xhtml

**RESULTS**

Within the total cohort (N=265,300), 93.7% (*n*=248,698) of patients were successfully matched by NHS Digital to the HES-ONS data via the PDS. The mean age for the matched sample was 43.40 (Range 5 months to 117 years; SD 22.69), at the time the patient identifiers were extracted, with half the sample being male (50.0%). The majority of the matched group (58.5%) were of white ethnicity and were resident in the four London Boroughs serviced by SLaM, i.e. Lambeth, Southwark, Lewisham, and Croydon (73.4%). The 'Strengthening The Reporting of Observational studies in Epidemiology' (STROBE) checklist [49] is reported in Table 1 of the Supplementary Material.

*Step 1: Missed Matches Analysis*

Table 3 provides the administrative, socio-demographic, and clinical characteristics of the CRIS sample, according to matched and unmatched status. With regards to the administrative variables examined (i.e. NHS number, date of birth, sex, and postcode present in PII sent to NHS Digital), records which did not have an NHS number or date of birth were not matched (*n*=5755, 2.17%). Records where sex was present in the PII sent to NHS Digital were eight times more likely to be matched than records where sex was not present (OR=8.14; 95% CI=4.66, 14.23; $p<0.001$). Similarly, records where a postcode was present were almost seven times more likely to be matched in comparison to records where no postcode was available for matching (OR=6.68; 95% CI= 6.26, 7.12; $p<0.001$). Patients who had died prior to the PII being extracted, i.e. 20th December 2016, (according to the gold standard CRIS-derived death data) were significantly more likely to match than patients who were alive.

Within the adjusted analysis, we found the likelihood of matching was significantly associated with a number of socio-demographic and clinical factors. Males and patients of non-white ethnicities, i.e. (i) mixed, (ii) Indian, Pakistani, Bangladeshi or 'other Asian', (iii) Caribbean, African, or 'other black', or (iv) other ethnicity, were all significantly less likely to match. Individuals without a stated ethnicity were also significantly less likely to match. Compared to patients in the lowest quartile of deprivation, patients in the second quartile were significantly less likely to match. We found no significant differences between patients in the third and fourth quartiles of deprivation ($p>0.05$).

With regards to clinical variables, patients who had been discharged or who had not had a referral in the two years prior to the PII being extracted were significantly less likely to match than patients who had an accepted referral in the past 2 years. Similarly, patients with the most face to face contact (third and fourth quartiles) were significantly more likely to match, whilst patients in the second quartile of face to face contact were significantly less likely to match. Patients with the highest number of inpatient bed days (32+) were significantly more likely to match than those with no inpatient bed days, whereas patients with a moderate number of inpatient bed days (3-31) were significantly less likely to match than those who no inpatient bed days.



For peer review only - http://bmjopen.bmj.com/site/about/guidelines.xhtml

In terms of diagnosis, patients who had ever received a primary diagnosis within the majority F00-98 ICD-10 diagnosis codes (see Table 3) were all significantly more likely to match than patients who had never received an ICD-10 F diagnosis. However, primary diagnoses of schizophrenia, schizotypal, and delusional disorders (F20-F29) or disorders of adult personality and behaviour (F60-F69) were not significantly associated with match status.

*Step 2: Optimal Match Analysis*

For records which were matched (*n*=248,698), we examined predictors of an optimal match. An optimal match represents a 'perfect match', i.e. where the records were matched on all four patient identifiers provided to NHS Digital (NHS number, sex, date of birth, and postcode). Within the adjusted model (see Table 2 in Supplementary Material) older age, male sex, an 'other' or 'not stated' ethnicity, living in the SLaM catchment area, deprivation, referral status in the two years prior to the linkage, low levels of face to face contact, and a mental health diagnosis were all significantly associated with an optimal match. All-cause mortality and number of inpatient bed days were not significantly associated with optimal match outcome.

*Step 3: CRIS Derived Mortality Analysis*

Table 4 displays clinical and socio-demographic factors associated with mortality using the gold standard CRIS-derived death data. Within the fully adjusted model (aOR[2]) older age, male sex, deprivation, a primary diagnosis ever of organic, including symptomatic, mental disorders (F00-F09), mental and behavioural disorders due to psychoactive substance use (F10-F19), schizophrenia, schizotypal and delusional disorders (F20-F29), mental retardation (F70-F79), amount of face to face contact, and inpatient bed days were all associated with a significant increased risk of mortality.

Whilst, non-white ethnicity, a primary diagnosis ever of mood (affective) disorders (F30-F39), neurotic, stress-related and somatoform disorders (F40-F49), behavioural syndromes associated with physiological disturbances and physical factors (F50-F59), behavioural and emotional disorders with onset usually occurring in childhood and adolescence (F90-F98), or other diagnoses were all associated with a significant decreased risk of mortality.

*Step 4: Linked Mortality Analysis*

We examined the associations between mortality and socio-demographic and clinical factors using the linked HES-ONS mortality data for the matched group only (*n*=248,698). Within the adjusted model (see Table 5) we found that older age, male sex, and the second, third and fourth (i.e. least deprived) quartiles of deprivation were significantly associated with mortality. With regards to clinical variables, a primary diagnosis of organic, including symptomatic, mental disorders (F00-F09), mental



For peer review only - http://bmjopen.bmj.com/site/about/guidelines.xhtml

and behavioural disorders due to psychoactive substance use (F10-F19), mental retardation (F70-F79), and a low, moderate, or high number of inpatient bed days were all significantly associated with an increased risk of mortality. Similarly, patients with higher levels of face to face contact (2nd and 3rd quartiles) were significantly more likely to have died when compared to patients with the least amount of face to face contact.

Non-white ethnicity, and a primary diagnosis of mood (affective) disorders (F30-F39), neurotic, stress-related and somatoform disorders (F40-F49), behavioural syndromes associated with physiological disturbances and physical factors (F50-F59), disorders of psychological development (F80-F89), and behavioural and emotional disorders with onset usually occurring in childhood and adolescence (F90-F98) were all negatively associated with mortality.

After further adjustments, using the optimal match variable and an inverse probability weighting, calculated during the missed match analysis in Step 1, to account for potential linkage bias, we found these associations with mortality persisted (Table 5).

We compared the output of the Step 3 and Step 4 analyses (Table 4 - $aOR^1$ and Table 5 - $aOR^2$), i.e. predictors of CRIS-derived mortality and predictors of linked ONS mortality in the matched group (controlling for optimal matching and matching probability). The two final models were largely the same with slight differences in some of the odds ratios. However, there was a difference between the models within the diagnosis variables. In the model predicting CRIS-derived mortality, a primary diagnosis ever of schizophrenia, schizotypal and delusional disorders (F20-F29) was significantly associated with all-cause mortality (OR=1.08; 95% CI=1.01, 1.15; $p$=0.02). Whereas in the model predicting linked mortality in the matched group, a primary diagnosis of F20-F29 was not significantly associated with mortality (OR=1.05; 95% CI=0.98, 1.13; $p$>0.05), despite the odds ratios being virtually identical (1.08 for the CRIS-derived death model compared to 1.05 for the linked death model). The only other difference between the two models was regarding the F60-F69 diagnosis variable. The association between a primary diagnosis of a disorder of adult personality and behaviour (F60-F69) and mortality in the CRIS-derived mortality model produced an odds ratio of 1.02, whereas this effect was reversed in the linked mortality model (OR: 0.98); however, neither of these findings were statistically significant.

*Post-hoc Analysis*

In Step 1 of the analysis we found that records which did not have an NHS number or date of birth were not matched. We therefore conducted a post-hoc analysis to investigate the association between sociodemographic factors (i.e. age, sex, ethnicity, resident in the SLaM catchment area, and deprivation) and presence of NHS number and date of birth for matching.



For peer review only - http://bmjopen.bmj.com/site/about/guidelines.xhtml

With regards to NHS number, 5755 (2.17%) individuals did not have an NHS number available for matching. After conducting a multivariate logistic regression analysis (see Table 3 in Supplementary Material), we found that all the sociodemographic variables examined were significantly associated with the availability of NHS number. Specifically, older age, mixed ethnicity, and being resident in the SLaM catchment area were positively associated with having an NHS number in the electronic patient record (EPR). Whereas, male sex, non-white ethnicity (except 'mixed') and the 2nd and 4th (least deprived) quartiles of deprivation were all negatively associated with having an NHS number in the EPR.

In terms of date of birth, only 290 (0.11%) records were missing a date of birth. In univariate logistic regression analysis (see Table 4 in Supplementary Material) Asian and 'Other' ethnicities were negatively associated with having a date of birth whilst the 2nd and 4th (last deprived) quartiles of deprivation were positively associated with having a date of birth in the EPR. Despite this, none of the sociodemographic variables examined were significantly associated with the availability of date of birth for matching when examined in multivariate logistic regression analysis.

**DISCUSSION**

*Statement of the Principal Findings*

By evaluating NHS electronic health records linked via standard deterministic procedures to HES-ONS mortality data, we found no evidence of substantial bias distorting risk-factor associations with the linked outcome measure analysed (i.e. mortality). Of the 265,300 SLaM records sent to NHS Digital for matching, 93.74% were successfully matched. Despite a number of significant administrative, socio-demographic, and clinical differences between records that matched and those that did not, we found minimal effects on the clinical associations with mortality. Matching error did not appear to impact the strength and direction of effects between mortality and a number of socio-demographic and clinical factors using linked mortality data after adjusting for matching probability using an inverse probability weighting statistical technique. Furthermore, after comparing the fully adjusted models predicting gold standard CRIS-derived mortality in the full cohort and linked mortality in the matched group we found that the two models were largely the same with only slight differences in some of the odds ratios.

*Putting the Results in Context*

Consistent with previous research, we found a number of significant socio-demographic differences between the matched and unmatched group including gender, ethnicity, and deprivation. Previous research has demonstrated how linkage error disproportionately affects marginalised people and, therefore, missed matches result in underestimating their needs [16,19-22,26-34]. In comparison



For peer review only

to the white ethnic group (i.e. British, Irish, or any other white ethnic groups), we found that individuals of all other recorded ethnicities examined were significantly less likely to match. This is potentially relevant to researchers examining health outcome variations by ethnicity, and those conducting studies within similarly diverse, urban populations [4]. We found that being resident in the SLaM catchment area was not associated with match status after controlling for all other examined sociodemographic and clinical factors. Generally, patients receiving care from SLaM national specialist services, as opposed to local area services, are of increased complexity and greater clinical contact which tends to improve administrative accuracy however, this does not seem to have had an impact on match status in the current data linkage.

This is one of the first studies to examine clinical factors associated with matching quality in a mental healthcare population and we identified a number of novel findings with regards to the clinical characteristics of the matched and unmatched groups. Patients who had not had a clinical contact with SLaM in the two years prior to the linkage were significantly less likely to be matched, whereas, patients with high levels of clinical contact, indicated by frequency of face to face contact or inpatient bed days, were significantly more likely to match. Similarly, patients who had ever received a primary diagnosis within the majority F00-98 ICD-10 diagnosis codes were significantly more likely to match than patients who had never received an ICD-10 F diagnosis. Our findings provide some indication that patients with higher severity of illness and clinical service contacts proximal to the date of matching are more likely to match, possibly through these factors driving greater administrative data accuracy. Risk factors for match status may operate through different mechanisms, e.g. (i) through the type of condition and severity, (ii) as determinants of linkage error, or (iii) both, and further research is required to understand the underlying mechanisms.

Improving the quality of data linkages, by reducing missing information in the source data, is important for scientific as well as legal and ethical reasons [25]. Within the current study we found that records which did not contain an NHS number or date of birth in the source SLaM data were not matched. In post-hoc analysis we found that the presence of NHS number in the EPR was significantly associated with a number of sociodemographic factors, including age, sex, and ethnicity. These findings give some indication that there is a bias in the recording of NHS numbers. Future work should aim to examine the reasons why some records are missing important administrative data in order to reduce the number of missing NHS numbers and dates of birth which would subsequently help improve the missed match rate and quality of future data linkages.

The evaluation of linkage quality can guide decisions about appropriate study designs [12]. However, data linkages tend to be carried out by an independent body and information about linkage processes are not always made available to researchers, known as the ‘separation principle’ [50]. By taking account of matching rank, in our sample we found that variation in linkage quality appeared to



For peer review only - http://bmjopen.bmj.com/site/about/guidelines.xhtml

have very little effect on the prediction of mortality. We were able to demonstrate this as the information on missed matches and match rank was provided to us by NHS Digital. Key to health researchers being able to account for the impact of matching on the analysis of interest (in our case clinical risk factor for death), are organisations which conduct data linkages being able to provide information on match accuracy (i.e. match rates, match ranks, etc.) so that potential linkage error can be accounted for in subsequent analyses. In the current study, matching was on a one to one basis (i.e. an individual was matched to a single record on the PDS), however, it is worth noting that in circumstances where matching is not one to one (e.g. matching individuals to multiple episodes of care) controlling for linkage error is more problematic even when information on match accuracy is provided.

*Strengths and Weaknesses of the Study*

This is a large-scale study of linkage error in a population of people receiving mental healthcare and one of the first studies to examine the association between patient characteristics and an optimal match. An optimal match (i.e. a match rank of one - see Table 2) indicates a higher quality match. Improving the number of records which achieve an optimal match is one way of improving the validity of the data linkage. By identifying factors associated with an optimal match we can target which specific records require additional administrative work (e.g. adding in missing information) to maximise the quality of the data linkage. Furthermore, the findings of this study may be generalisable to other NHS cohorts, as the quality and type of administrative data available for matching is likely to be similar within most NHS Trusts. Furthermore, standard NHS Digital matching methodology was used for the data linkage.

Gold standard data are rarely available but provide an easily interpretable way to measure linkage error [12], a major strength of the study is the use of gold standard death data (i.e. CRIS-derived mortality) as a reference category for examining error in the linked records. Within the gold-standard data we would expect the majority of deaths to be picked up either via automatic tracing or through the Trust being informed directly, for example via family members. However, there may be situations where the Trust is not aware of the death of a patient, for example if the patient died outside of the UK. Furthermore, matching error due to false or missed matches is not the only reason that we may see discrepancies between the CRIS-derived mortality data and the linked mortality data from NHS Digital. For example, if the death was not registered in the UK it would not be included in the mortality data obtained from the ONS. Similarly, there are sometimes delays in death registration due to coroner investigations which can take several months. Although SLaM may be informed at the time of death, and therefore the EPR updated to reflect this, it could be several months before the official registration takes places. Despite this, it is not always possible to identify potential missing



For peer review only - http://bmjopen.bmj.com/site/about/guidelines.xhtml

death data or the reasons for discrepancies within the data and therefore researchers should be aware of these issues when analysing linked data.

There are some limitations of this study. Within the unmatched group, we were unable to tell apart missed matches and patients who had opted out of their data being used for secondary purposes. This is due to the strict governance requirements around opt-outs [47]. However, any researcher using data from NHS Digital, will have to account for missed matching in the same way, irrespective of whether non-matching was due to opt-out. Within the current study, it was not possible for us to tease apart the impact of providing a full or part postcode on match status. In future, it may be beneficial to separate the postcode variable in order to determine whether submitting full or part postcodes has an impact on matching. Furthermore, we only examined missed matches and not false matches, and therefore we may have underestimated the effect of linkage error. Future research should aim to examine the rate of false matches in order to gain a clearer picture of the total effect of linkage error in this population.

The data linkage in the current study was conducted by NHS Digital using deterministic matching methods; however, previous research has found that following probabilistic linkage methods can reduce the number of missed matches. With regards to HES data specifically, a previous study found that the inclusion of a probabilistic matching step to the algorithm used to link together episodes of care reduced the number of missed matches which led to less biased estimates of hospital readmissions rates for certain patient groups such as ethnic minorities [26]. Similarly, in a data linkage between the National Neonatal Research Database and national laboratory infection surveillance data, deterministic match rates improved in later years due to patient identifiers being more complete, but despite this, probabilistic linkage was still able to identify matches not found using deterministic linkage alone [51]. Probabilistic matching is not currently a service provided as standard by NHS Digital, however going forward, the inclusion of probabilistic matching techniques may help to reduce the number of missed matches in future refreshes of the linkage in the current study.

*Conclusion*

There are significant administrative, socio-demographic, and clinical differences between the matched and unmatched records in the data linkage examined. Despite this, after adjusting for matching probability, missed matches do not appear to have had an effect on the prediction of mortality. Furthermore, after comparing models predicting gold standard CRIS-derived mortality and linked mortality we found that the models were largely the same with only slight differences in some of the odds ratios. Together these provide some indication that this effect is not driven by selection bias from matching error and provide some reassurance to studies using NHS linked data to investigate mortality.



For peer review only - http://bmjopen.bmj.com/site/about/guidelines.xhtml

**Acknowledgements**

We would like to acknowledge NHS Digital as the provider of the HES and mortality data; those who carried out the original collection and analysis of the data bear no responsibility for their further analysis or interpretation. We would also like to acknowledge the staff at the Maudsley Biomedical Research Centre (BRC) Nucleus for their continued support of the study.

**Contributors**

AJ and JD conceived the study design. Data extraction was completed by AJ with support from JD and MB. Data analysis was conducted by AJ and JD. Reporting of findings was led by AJ and JD with support from MB, RG, RH, and RS. All authors contributed to the manuscript preparation and approved the final version.

**Funding**

This paper represents independent research part funded by the National Institute for Health Research (NIHR) Biomedical Research Centre (BRC) at the South London and Maudsley NHS Foundation Trust and King's College London. The work described here was part-supported by the Mental Health Data Pathfinder award to King's College London from the Medical Research Council (reference MC_PC_17214). The views expressed are those of the authors and not necessarily those of the NHS, the NIHR, the MRC, or the Department of Health. AJ, MB, RH, and RS receive salary support from the National Institute for Health Research (NIHR) Mental Health Biomedical Research Centre at South London and Maudsley NHS Foundation Trust and King's College London.

**Competing Interests**

RH has received research funding from Roche, Pfizer, Janssen, and Lundbeck. The other authors have declared that no competing interests exist.

**Ethics Approval**

CRIS was approved as a dataset for secondary analysis by the Oxford Research Ethics Committee C (18/SC/0372). Approval to conduct the data linkage was granted by the Health Research Authority (HRA) Confidentiality Advisory Group (CAG) in July 2011 (ECC 3-04(f)/2011) and by NHS Digital's Data Access Advisory Group (DAAG) in October 2016 (NIC-292279-Z2S5T). Approval to conduct the study was granted in July 2017 by the CRIS Oversight Committee (17-065).

**Data Sharing Statement**

For peer review only

Data are owned by a third party, Maudsley Biomedical Research Centre (BRC) Clinical Records Interactive Search (CRIS) tool, which provides access to anonymised data derived from SLaM electronic medical records. These data can only be accessed by permitted individuals from within a secure firewall (i.e., the data cannot be sent elsewhere), in the same manner as the authors. For more information please contact: cris.administrator@slam.nhs.uk.

For peer review only

**REFERENCES**

1. Chesney, E., Goodwin, G.M. and Fazel, S. (2014) Risks of all-cause and suicide mortality in mental disorders: a meta-review. *World Psychiatry*, 13, p.p. 153-160.

2. Walker, E.R., McGee, R.E. and Druss, B.G. (2015) Mortality in Mental Disorders and Global Disease Burden Implications: A Systematic Review and Meta-analysis. *JAMA Psychiatry*, 72(4), p.p. 334-341.

3. Ilyas, A., Chesney, E. and Patel, R. (2017) Improving life expectancy in people with serious mental illness: should we place more emphasis on primary prevention. *The British Journal of Psychiatry*, 211(4), p.p. 194-197.

4. Perera, G., Broadbent, M., Callard, F., Chang, C-K., Downs, J., Dutta, R., Fernandes, A., Hayes, R.D., Henderson, M., Jackson, R., Jewell, A., Kadra, G., Little, R., Pritchard, M., Shetty, H., Tulloch, A., Stewart, R. (2016) Cohort profile of the South London and Maudsley NHS Foundation Trust Biomedical Research Centre (SLaM BRC) Case Register: current status and recent enhancement of an Electronic Mental Health Record- derived data resource. *BMJ Open*, 6:e008721.

5. Chang, C-K., Hayes, R.D., Perera, G., Broadbent, M.T.M., Fernandes, A.C., Lee, W.E., Hotopf, M. and Stewart, R. (2011) Life Expectancy at Birth for People with Serious Mental Illness and Other Major Disorders from a Secondary Mental Health Care Case Register in London. *PLoS ONE*, 6(5): e19590.

6. Fok, M.L-Y, Hayes, R.D., Chang, C-K., Stewart, R., Callard, F.J. and Moran, P. (2012) Life expectancy at birth and all-cause mortality among people with personality disorder. *Journal of Psychosomatic Research*, 73(2), p.p. 104-107.

7. Hayes, R.D., Chang, C-K., Fernandes, A., Begum, A., To, D., Broadbent, M., Hotopf, M. and Stewart, R. (2012) Associations between symptoms and all-cause mortality in individuals with serious mental illness. *Journal of Psychosomatic Research*, 72(2), p.p. 114-119.



For peer review only - http://bmjopen.bmj.com/site/about/guidelines.xhtml

8. Das-Munshi, J., Chang, C-K., Dutta, R., Morgan, C., Nazroo, J., Stewart, R. and Prince, M.J. (2017) Ethnicity and excess mortality in severe mental illness: a cohort study. *The Lancet Psychiatry*, 4, p.p. 389-399.

9. Jayatilleke, N., Hayes, R.D., Dutta, R., Shetty, H., Hotopf, M., Chang, C-K. and Stewart, R. (2017) Contributions of specific causes of death to lost life expectancy in severe mental illness. *European Psychiatry*, 43, p.p. 109-115.

10. Green, E., Ritchie, F., Mytton, J., Webber, D.J., Deave, T., Montgomery, A., Woolfrey, L., ul-Baset, K. and Chowdhury, S. (2015) Enabling Data Linkage to Maximise the Value of Public Health Research Data: full report. Project Report. Wellcome Trust, London. [Online] Available from: https://wellcome.ac.uk/sites/default/files/enabling-data-linkage-to-maximise-value-of-public-health-research-data-phrdf-mar15.pdf. [Accessed 7 March 2018].

11. Bohensky, M.A., Jolley, D., Sundararajan, V., Evans, S., Pilcher, D.V., Scott, I. and Brand, C.A. (2010) Data Linkage: A powerful research tool with potential problems. *BMC Health Services Research*, 10:346.

12. Harron, K.L., Doidge, J.C., Knight, H.E., Gilbert, R.E., Goldstein, H., Cromwell, D.A. and van der Meulen, J.H. (2017) A guide to evaluating linkage quality for the analysis of linked data. *International Journal of Epidemiology*, 0 (0), p.p. 1-12.

13. Blakely, T. and Salmond, C. (2002) Probabilistic record linkage and a method to calculate the positive predictive value. *International Journal of Epidemiology*, 31, p.p. 1246-1252.

14. Buescher, P.A. (1999) Method of Linking Medicaid Records to Birth Certificates May Affect Infant Outcome Statistics. *American Journal of Public Health*, 89(4), p.p. 564-566.

15. Bopp, M. and Minder, C.E. (2003) Mortality by education in German speaking Switzerland, 1990-1997: results from the Swizz National Cohort. *International Epidemiological Association*, 32, p.p. 346-354.

16. Zingmond, D.S., Ye, Z., Ettner, S.L. and Honghu, L. (2004) Linking hospital discharge and death records - accuracy and sources of bias. *Journal of Clinical Epidemiology*, 57, p.p. 21-29.




For peer review only - http://bmjopen.bmj.com/site/about/guidelines.xhtml

17. Jebamani, L.S., Burchill, C.A. and Martens, P.J. (2005) Using data linkage to identify First National Manitobans: technical, ethical, and political issues. *Canadian Journal of Public Health*, 96(1): S28-32.

18. Maizlish, N.A. and Herrera, L. (2005) A Record Linkage Protocol for a Diabetes Registry at Ethnically Diverse Community Health Centers. *Journal of the American Medical Informatics Association*, 12(3), p.p. 331-337.

19. Schmidlin, K., Clough-Gorr, K.M., Spoerri, A., Egger, M. and Zwahlen, M. (2013) Impact of unlinked deaths and coding changes on mortality trends in the Swiss National Cohort. *BMC Medical Informatics and Decision Making*, 13:1.

20. Kabudula, C.W., Clark, B.D., Gómez-Olive, F.X., Tollman, S., Menken, J. and Reniers, G. (2014) The promise of record linkage for assessing the uptake of health services in resource constrained settings: a pilot study from South Africa. *BMC Medical Research Methodology*, 14:71.

21. McIsaac, K.E., MacDonald, S.F., Chong, N., Moser, A., Moineddin, R., Colantonio, A., Nathens, A. and Matheson, F.I. (2016) Creating a powerful platform to explore health in a correctional population: A record linkage study. *PLOS one*, 11(8): e0161173.

22. Rosman, D.L. and Knuiman, M.W. (1994) A comparison of hospital and police road injury data. *Accident; Analysis and Prevention,* 26(2), p.p. 215-222.

23. Cryer, P.C., Westrup, S., Cook, A.C., Ashwell, V., Bridger, P. and Clarke, C. (2001) Investigation of bias after data linkage of hospital admissions data to police road traffic crash reports. *Injury Prevention,* 7, p.p. 234-241.

24. Clark, D., King, A., Sharpe, K., Connelly, G., Elliot, L., Macpherson, L.M.D., McMahon, A.D., Milligan, I., Wilson, P., Conway, D.I. and Wood, R. (2017) Linking routinely collected social work, education and health data to enable monitoring of the health and health care of school-aged children in state care ('looked after children') in Scotland: a national demonstration project. *Public Health*, 150, p.p. 101-111.



For peer review only - http://bmjopen.bmj.com/site/about/guidelines.xhtml

25. Hagger-Johnson, G., Harron, K., Fleming, T., Gilbert, R., Goldstein, H., Landy, R. and Parslow, R.C. (2015) Data linkage errors in hospital administrative data when applying a pseudonymisation algorithm to paediatric intensive care records. *BMJ Open*, 5:e008118.

26. Hagger-Johnson, G., Harron, K., Goldstein, H., Aldridge, R. and Gilbert, R. (2017) Probabilistic linking to enhance deterministic algorithms and reduce linkage errors in hospital administrative data. *Journal of Innovation in Health Informatics*, 24(2), p.p. 234-246.

27. Downs, J.M., Ford, T., Stewart, R., Epstein, S., Shetty, H., Little, R., Jewell, A., Broadbent, M., Deighton, J., Mostafa, T., Gilbert, R., Hotopf, M. and Hayes, R. (2019) An approach to linking education, social care and electronic health records for children and young people in South London: a linkage study of child and adolescent mental health service data. *BMJ Open*, 9:e024355.

28. Adams, M.M., Wilson, H.G., Casto, D.L., Berg, C.J., McDermott, J.M., Gaudino, J.A. and McCarthy, B.J. (1997) Constructing Reproductive Histories by Linking Vital Records. *American Journal of Epidemiology*, 145(4), p.p. 339-348.

29. Blakely, T., Woodward, A. and Salmond, C. (2000) Anonymous linkage of New Zealand mortality and Census data. *Australian and New Zealand Journal of Public Health,* 24(1), p.p. 92-95.

30. Gyllstrom, M.E., Jensen, J.L., Vaughan, J.N., Castellano, S.E. and Oswald, J.W. (2002) Linking Birth Certificates with Medicaid Data to Enhance Population Health Assessment: Methodological Issues Addressed. *Journal of Public Health Management Practice,* 8(4), p.p. 38-44.

31. Ringland, C., Correll, P.K., Lim, K., Williamson, M. and Marks, G.B. (2006) Hospital readmissions for asthma: a feasibility study comparing strategies for linking hospital morbidity data. *Australian and New Zealand Journal of Public Health,* 30(5), p.p. 435-439.

32. Lariscy, J.T. (2011) Differential Record Linkage by Hispanic Ethnicity and Age in Linked Mortality Studies: Implications for the Epidemiologic Paradox. *Journal of Aging and Health*, 23(8), p.p. 1263-1284.



For peer review only - http://bmjopen.bmj.com/site/about/guidelines.xhtml

33. Beckmann, K.R., Bennett, A., Young, G.P. and Roder, D.M. (2014) Treatment patterns among colorectal cancer patients in South Australia: a demonstration of the utility of population-based data linkage. *Journal of Evaluation in Clinical Practice*, 20, p.p. 467-477.

34. Moore, C.L., Gidding, H.F., Law, M.G. and Amin, J. (2016) Poor record linkage sensitivity biased outcomes in a linked cohort analysis. *Journal of Clinical Epidemiology*, 75, p.p. 70-77.

35. Baldwin, E., Johnson, K., Berthoud, H. and Dublin, S. (2015) Linking mothers and infants within electronic health records: a comparison of deterministic and probabilistic algorithms. *Pharmacoepidemiology and Drug Safety*, 24, p.p. 45-51.

36. Harron, K., Dibben, C., Boyd, J., Hjern, A., Azimaee, M., Barreto, M.L. and Goldstein, H. (2017) Challenges in administrative data linkage for research. *Big Data & Society*, 4(2), p.p. 1-12.

37. Stewart, R., Soremekun, M., Perera, G., Broadbent, M., Callard, F., Denis, M., Hotopf, M., Thornicroft, G. and Lovestone, S. (2009) The South London and Maudsley NHS Foundation Trust Biomedical Research Centre (SLAM BRC) case register: development and descriptive data. *BMC Psychiatry*, 9:51.

38. Downs, J., Gilbert, R., Hayes, R.D., Hotopf, M. and Ford, T. (2017) Linking health and education data to plan evaluate services for children. *Archives of Disease in Childhood*, 102, p.p. 599-602.

39. Chang, C-K., Hayes, R.D., Broadbent, M.T.M., Hotopf, M., Davies, E., Møller, H. and Stewart, R. (2014) A cohort study on mental disorders, stage of cancer at diagnosis and subsequent survival. *BMJ Open*, 4:e004295.

40. Woodhead, C., Ashworth, M., Broadbent, M., Callard, F., Hotopf, M., Schofield, P., Soncul, M., Stewart, R.J. and Henderson, M.J. (2016) Cardiovascular disease treatment among patients with server mental illness: a data linkage study between primary and secondary care. British *Journal of General Practice*, 66, p.p. e374-e381.

41. NHS (2019) PDS Death Notification Status Code; Description. [Online] Available from: https://www.datadictionary.nhs.uk/data_dictionary/attributes/p/paya/pds_death_notification_status_code_de.asp?shownav=1



For peer review only - http://bmjopen.bmj.com/site/about/guidelines.xhtml

42. NHS Digital (2019) Summary Care Records (SCR). [Online] Available from: https://digital.nhs.uk/services/summary-care-records-scr [Accessed 16 July 2019].

43. Academy of Medical Royal Collages. Hospital Episode Statistics (HES): improving the quality and value of hospital data. A discussion document. Leeds: Health and Social Care Information Centre, 2011. [Online] Available from: http://content.digital.nhs.uk/media/1593/Hospital-Episode-Statistics-Improving-the-quality-and-value-of-hospital-data/pdf/HES__Improving_the_quality_and_value_of___hospital_data.pdf [Accessed 9 February 2018].

44. NHS Digital (2019) Secondary Uses Service (SUS). [Online] Available from: https://digital.nhs.uk/services/secondary-uses-service-sus. [Accessed 18 January 2019].

45. NHS Digital (2019) Primary Care Mortality Database. [Online] Available from: https://digital.nhs.uk/services/primary-care-mortality-database. [Accessed 16 July 2019].

46. NHS Digital (2019) Demographics. [Online] Available from: https://digital.nhs.uk/services/demographics. [Accessed 2 April 2019].

47. NHS Digital (2018) Information on type 2 opt-outs. [Online] Available from: https://digital.nhs.uk/data-access-request-service/type-2-opt-outs. [Accessed 6 March 2018].

48. Jewell, A., Pritchard, M., Barrett, K., Green, P., Markham, S., McKenzie, S., Oliver, R., Wan, M., Downs, J. and Stewart, R. (2019) The Maudsley Biomedical Research Centre (BRC) data linkage service user and carer advisory group: creating and sustaining a successful patient and public involvement group to guide research in a complex area. *Research Engagement and Involvement*, 5:20.

49. Elm, E.V., Altman, D.G., Egger, M., Pocock, S.J., Gøtzche, P.C., Vanderbroucke, J.P., STROBE Initiative (2007) Strengthening the reporting of observational studies in epidemiology (STROBE) statement: guidelines for reporting observational studies. BMJ, 335:806.


For peer review only



For peer review only - http://bmjopen.bmj.com/site/about/guidelines.xhtml

50. Gilbert, R., Lafferty, R., Hagger-Johnson, G., Harron, K., Zhang, L-C, Smith, P., Dibben, C. and Goldstein, H. (2018) GUILD: GUidance for Information about Linking Data sets. *Journal of Public Health*, 40(1), p.p. 191-198.

51. Fraser, C., Muller-Pebody, B., Blackburn, R., Gray, J., Oddie, S.J., Gilbert, R.E. and Harron, K. (2019) Linking Surveillance and Clinical Data for Evaluating Trends in Bloodstream Infection Rates in Neonatal Units in England. *PLoS ONE*, 14(12):e0226040.

For peer review only



For peer review only - http://bmjopen.bmj.com/site/about/guidelines.xhtml

**Figure Captions**

**Figure 1.** Flow diagram describing how the gold standard CRIS-derived date of death is entered into the SLaM electronic medical record.

**Figure 2.** Flow diagram of the linkage process between SLaM records and HES- ONS mortality data.

For peer review only



For peer review only - http://bmjopen.bmj.com/site/about/guidelines.xhtml

**Table 1.** Exposure variables.

| Exposure Variable | Description |
|---|---|
| NHS number present | Was NHS number available for linkage, binary variable (yes vs. no). |
| Date of birth present | Was date of birth available for linkage, binary variable (yes vs. no). |
| Sex present | Was sex available for linkage, binary variable (yes vs. no). |
| Postcode present | Was postcode (full or part) available for linkage, binary variable (yes vs. no). |
| Age | Age in years at time of PII extraction (20th December 2016). |
| Sex | Sex, male vs. female. |
| Patient deceased | Is the patient deceased according to the gold standard CRIS-derived mortality data (yes vs. no). |
| Ethnicity | Patient ethnicity, coded into 6 categories (i) British, Irish, or any other white ethnic groups, (ii) mixed, (iii) Indian , Pakistani, Bangladeshi, or 'other Asian', (iv) Caribbean, African, or 'other black', (v) other, and (vi) not stated. |
| Resident in SLaM catchment area | Was the patient resident in the SLaM catchment area, i.e. Lambeth, Lewisham, Southwark, or Croydon, at the time of PII extraction, binary variable (yes vs. no). |
| Quartiles of neighbourhood deprivation | Quartiles of deprivation score: 1st (most deprived), 2nd, 3rd, 4th (least deprived). |
| Referral Status in past two years | Referral status in the two years prior to PII extraction; (i) accepted, (ii) discharged, (iii) rejected, or (iv) no referral in 2 years prior to linkage. |



For peer review only - http://bmjopen.bmj.com/site/about/guidelines.xhtml

| | |
|---|---|
| Primary Diagnosis Ever | A primary diagnosis ever of F00-F09: organic, including symptomatic, mental disorders, F10-F19: mental and behavioural disorders due to psychoactive substance use, F20-F29: schizophrenia, schizotypal and delusional disorders, F30-F39: mood (affective) disorders, F40-F49: neurotic, stress-related and somatoform disorders, F50-F59: behavioural syndromes associated with physiological disturbances and physical factors, F60-F69: disorders of adult personality and behaviour, F70-F79: mental retardation, F80-89: disorders of psychological development, F90-F98: behavioural and emotional disorders with onset usually occurring in childhood and adolescence, or Other diagnosis. Binary variables (yes vs. no). Other diagnosis category includes all non-mental health (i.e. non F code) ICD-10 diagnoses as well as 'F99: Mental disorder, not otherwise specified' and 'F00-F99: Mental and behavioural disorders'. Patients can have multiple primary diagnoses throughout their time at SLaM and therefore can appear in multiple groups. |
| Face to face contact | Quartiles of face to face contact with SLaM from 1st January 2006 to 20th December 2016; 1st (least face to face contact), 2nd, 3rd, 4th (most face to face contact). |
| Inpatient bed days | Number of SLaM inpatient bed days from 1st January 2006 to 20th December 2016, coded into; (i) none (0), (ii) low (1-2 days), (iii) moderate (3-31 days), and (iv) high (32+). |
| Optimal match | Binary variable (optimal match vs. non-optimal match). For those records which were successfully matched by NHS Digital only. An optimal match represents a 'perfect' match or a match rank of one (see Table 2), i.e. the records matched on all supplied patient identifiers (NHS number, sex, date of birth, and postcode). |



For peer review only - http://bmjopen.bmj.com/site/about/guidelines.xhtml

**Table 2.** NHS Digital deterministic matching steps.

| Match Rank | NHS Number | Date of Birth | Sex | Postcode[a] | |
|---|---|---|---|---|---|
| 1 | Exact | Exact | Exact | Exact | |
| 2 | Exact | Exact | Exact | | |
| 3 | Exact | Partial | Exact | Exact | |
| 4 | Exact | Partial | Exact | | |
| 5 | Exact | | | Exact | |
| 6 | | Exact | Exact | Exact | where NHS number does not contradict the match and DOB is not 1 January and the Postcode is in the 'ignore' list[b] |
| 7 | | Exact | Exact | Exact | where NHS number does not contradict the match and DOB is not 1 January |
| 8 | Exact | | | | |

**Note.** [a] Current or most recently recorded postcode. [b] The 'ignore' list includes postcodes for communal establishments such as hospitals, care homes, prisons, and boarding schools.

For peer review only

**Table 3.** Logistic regression analysis examining administrative, socio-demographic, and clinical associations with match status (match vs. missed match).

| Variable | Total Population (N=265,300) | Matched (*n*=248,698 93.74%) | Missed Match (*n*=16,602 6.26%) | OR (95% CI) | aOR[1] (95% CI) |
|---|---|---|---|---|---|
| **Administrative variables** | | | | | |
| NHS number present: *n* (%) | 259545 (97.83) | 248698 (100.00) | 10847 (65.34) | | |
| Date of birth present: *n* (%) | 265010 (99.89) | 248698 (100.00) | 16312 (98.25) | | |
| Sex present: *n* (%) | 265246 (99.98) | 248663 (99.99) | 16583 (99.89) | **8.14 (4.66-14.23)**** | |
| Postcode present: *n* (%) | 260466 (98.18) | 245278 (98.62) | 15188 (91.48) | **6.68 (6.26-7.12)**** | |
| **Sociodemographic variables** | | | | | |
| Age: mean (SD) | 43.40 (22.69) | 43.50 (22.79) | 41.92 (21.09) | **1.003 (1.002-1.004)**** | 0.999 (0.998-1.000) |
| Male Sex: *n* (%) | 132730 (50.04) | 123818 (49.79) | 8912 (53.74) | **0.85 (0.83-0.88)**** | **0.93 (0.89-0.96)**** |
| Patient deceased: *n* (%) | 30173 (11.37) | 29057 (11.68) | 1116 (6.72) | **1.84 (1.73-1.95)**** | **1.41 (1.30-1.53)**** |
| Ethnicity: *n* (%) | | | | | |
| British, Irish, or any other white ethnic groups | 138495 (58.54) | 131150 (58.93) | 7345 (52.35) | (reference) | (reference) |
| Mixed | 6853 (2.90) | 6477 (2.91) | 376 (2.68) | 0.96 (0.87-1.07) | **0.82 (0.73-0.91)**** |
| Indian, Pakistani, Bangladeshi, or 'other Asian' | 10889 (4.60) | 10155 (4.56) | 734 (5.23) | **0.77 (0.72-0.84)**** | **0.78 (0.71-0.85)**** |
| Caribbean, African, or 'other black' | 40725 (17.21) | 38013 (17.08) | 2712 (19.33) | **0.78 (0.75-0.82)**** | **0.70 (0.66-0.74)**** |
| Other | 16650 (7.04) | 15393 (6.92) | 1257 (8.96) | **0.69 (0.64-0.73)**** | **0.77 (0.72-0.83)**** |
| Not stated | 22961 (9.71) | 21354 (9.60) | 1607 (11.45) | **0.74 (0.70-0.79)**** | **0.89 (0.83-0.95)*** |
| Resident in SLaM catchment area: *n* (%) | 187773 (73.42) | 177766 (73.53) | 10007 (71.47) | **1.11 (1.07-1.15)**** | 0.97 (0.93-1.02) |
| Quartiles of neighbourhood deprivation: *n* (%) | | | | | |
| 1st (most deprived) | 63476 (25.03) | 60220 (25.09) | 3256 (24.00) | (reference) | (reference) |
| 2nd | 63452 (25.02) | 59786 (24.91) | 3666 (27.02) | **0.88 (0.84-0.93)**** | **0.90 (0.86-0.95)**** |
| 3rd | 63449 (25.02) | 60052 (25.02) | 3397 (25.04) | 0.96 (0.91-1.00) | 1.01 (0.95-1.07) |
| 4th (least deprived) | 63221 (24.93) | 59973 (24.99) | 3248 (23.94) | 1.00 (0.95-1.05) | 1.06 (1.00-1.12) |
| **Clinical variables** | | | | | |
| Referral status in 2 years prior to linkage: *n* (%) | | | | | |
| Accepted | 12041 (4.56) | 11423 (4.61) | 618 (3.78) | (reference) | (reference) |
| Discharged | 45228 (17.11) | 42394 (17.10) | 2834 (17.33) | **0.81 (0.74-0.89)**** | **0.87 (0.78-0.97)*** |
| Rejected | 9628 (3.64) | 8921 (3.60) | 707 (4.32) | **0.68 (0.61-0.76)**** | 0.88 (0.76-1.01) |

For peer review only



For peer review only - http://bmjopen.bmj.com/site/about/guidelines.xhtml

| | | | | | |
|---|---|---|---|---|---|
| No referral in 2 years prior to linkage | 197414 (74.69) | 185221 (74.70) | 12193 (74.57) | **0.82 (0.76-0.89)*** | **0.87 (0.79-0.95)*** |
| Primary diagnosis ever: *n (%)* | | | | | |
| F00-F09: organic, including symptomatic, mental disorders | 25249 (9.52) | 24248 (9.75) | 1001 (6.03) | **1.68 (1.58-1.80)**** | **1.37 (1.26-1.49)**** |
| F10-F19: mental and behavioural disorders due to psychoactive substance use | 27755 (10.46) | 26158 (10.52) | 1597 (9.62) | **1.10 (1.05-1.16)**** | **1.12 (1.05-1.21)*** |
| F20-F29:schizophrenia, schizotypal and delusional disorders | 18516 (6.98) | 17323 (6.97) | 1193 (7.19) | 0.97 (0.91-1.03) | 1.02 (0.93-1.11) |
| F30-F39: mood (affective) disorders | 42578 (16.05) | 40515 (16.29) | 2063 (12.43) | **1.37 (1.31-1.44)**** | **1.28 (1.21-1.36)**** |
| F40-F49: neurotic, stress-related and somatoform disorders | 33248 (12.53) | 31599 (12.71) | 1649 (9.93) | **1.32 (1.25-1.39)**** | **1.27 (1.19-1.35)**** |
| F50-F59: behavioural syndromes associated with physiological disturbances and physical factors | 8743 (3.30) | 8388 (3.37) | 355 (2.14) | **1.60 (1.43-1.78)**** | **1.49 (1.31-1.69)**** |
| F60-F69: disorders of adult personality and behaviour | 6945 (2.62) | 6585 (2.65) | 360 (2.17) | **1.23 (1.10-1.37)**** | 0.95 (0.84-1.07) |
| F70-F79: mental retardation | 3018 (1.14) | 2898 (1.17) | 120 (0.72) | **1.62 (1.35-1.95)**** | **1.55 (1.26-1.89)**** |
| F80-F89: disorders of psychological development | 7242 (2.73) | 6991 (2.81) | 251 (1.51) | **1.88 (1.66-2.14)**** | **1.86 (1.61-2.15)**** |
| F90-F98: behavioural and emotional disorders with onset usually occurring in childhood and adolescence | 17011 (6.41) | 16363 (6.58) | 648 (3.90) | **1.74 (1.60-1.88)**** | **1.58 (1.45-1.74)**** |
| Other diagnosis | 119638 (45.10) | 112556 (45.26) | 7082 (42.66) | **1.11 (1.08-1.15)**** | **1.23 (1.18-1.28)**** |
| Quartiles of face to face contacts: *n* (%) | | | | | |
| $1^{st}$ (least face to face contact) | 76602 (28.87) | 70822 (28.48) | 5780 (34.82) | (reference) | (reference) |
| $2^{nd}$ | 64914 (24.47) | 59811 (24.02) | 5103 (30.74) | **0.96 (0.92-0.99)*** | **0.94 (0.89-0.99)*** |
| $3^{rd}$ | 59337 (22.37) | 56116 (22.56) | 3221 (19.40) | **1.42 (1.36-1.49)**** | **1.15 (1.08-1.22)**** |
| $4^{th}$ (most face to face contact) | 64447 (24.29) | 61949 (24.91) | 2498 (15.05) | **2.02 (1.93-2.12)**** | **1.42 (1.33-1.52)**** |
| Inpatient bed days: *n* (%) | | | | | |
| None (0) | 238573 (89.93) | 223527 (89.88) | 15046 (90.63) | (reference) | (reference) |
| Low (1-2 days) | 1446 (0.55) | 1342 (0.54) | 104 (0.63) | 0.87 (0.71-1.06) | 0.86 (0.67-1.09) |
| Moderate (3-31 days) | 9825 (3.70) | 9133 (3.67) | 692 (4.17) | **0.89 (0.82-0.96)*** | **0.90 (0.81-1.00)*** |
| High (32+ days) | 15456 (5.83) | 14696 (5.91) | 760 (4.58) | **1.30 (1.21-1.40)**** | **1.14 (1.03-1.26)*** |

**Note.** *$p<0.05$. **$p<0.001$. [1]adjusted for all other variables listed in the table. Missing data: age (*n*=275); sex (*n*=55); ethnicity (*n*=28,727); resident in local catchment area (*n*=9,537); quartiles of neighbourhood deprivation (*n*=11,702); referral status (*n*=989).



For peer review only - http://bmjopen.bmj.com/site/about/guidelines.xhtml

**Table 4.** Logistic regression analysis examining socio-demographic and clinical associations with gold standard CRIS-derived mortality.

| Variable | Total Population (N=265,300) | Patient Deceased (*n*=30,173 11.37%) | Patient Alive (*n*=235,127 88.63%) | OR (95% CI) | aOR[1] (95% CI) |
|---|---|---|---|---|---|
| **Sociodemographic variables** | | | | | |
| Age: mean (SD) | 43.40 (22.69) | 77.32 (19.06) | 39.05 (19.17) | **1.090 (1.089-1.091)**** | **1.092 (1.090-1.093)**** |
| Male Sex: *n* (%) | 132730 (50.04) | 14848 (49.21) | 117882 (50.15) | **0.96 (0.94-0.99)*** | **1.48 (1.43-1.53)**** |
| Ethnicity coded: *n* (%) | | | | | |
| British, Irish, or any other white ethnic groups | 138495 (58.54) | 21993 (78.19) | 116502 (55.89) | (reference) | (reference) |
| Mixed | 6853 (2.90) | 208 (0.74) | 6645 (3.19) | **0.17 (0.14-0.19)**** | **0.73 (0.61-0.86)**** |
| Indian, Pakistani, Bangladeshi, or 'other Asian' | 10889 (4.60) | 1006 (3.58) | 9883 (4.74) | **0.54 (0.50-0.58)**** | **0.70 (0.65-0.76)**** |
| Caribbean, African, or any 'other black' | 40725 (17.21) | 2914 (10.36) | 37811 (18.14) | **0.41 (0.39-0.43)**** | **0.63 (0.60-0.66)**** |
| Other | 16650 (7.04) | 705 (2.51) | 15945 (7.65) | **0.23 (0.22-0.25)**** | **0.52 (0.48-0.57)**** |
| Not stated | 22961 (9.71) | 1301 (4.63) | 705 (2.51) | **0.32 (0.30-0.34)**** | **0.91 (0.84-0.98)*** |
| Resident in SLaM catchment area: *n* (%) | 187773 (73.42) | 23870 (81.41) | 163903 (72.38) | **1.67 (1.62-1.72)**** | **0.84 (0.80-0.88)**** |
| Quartiles of neighbourhood deprivation: *n* (%) | | | | | |
| 1st (most deprived) | 63476 (25.03 | 7626 (26.11) | 55850 (24.89) | (reference) | (reference) |
| 2nd | 63452 (25.02) | 7049 (24.13) | 56403 (25.14) | **0.92 (0.88-0.95)**** | **1.08 (1.03-1.13)*** |
| 3rd | 63449 (25.02) | 7375 (25.25) | 56074 (24.99) | **0.96 (0.93-1.00)*** | **1.25 (1.19-1.31)**** |
| 4th (least deprived) | 63221 (24.93) | 7161 (24.51) | 56060 (24.98) | **0.94 (0.90-0.97)**** | **1.31 (1.25-1.37)**** |
| **Clinical variables** | | | | | |
| Primary diagnosis ever: *n (%)* | | | | | |
| F00-F09: organic, including symptomatic, mental disorders | 25249 (9.52) | 12641 (41.90) | 12608 (5.36) | **12.73 (12.36-13.10)**** | **1.10 (1.05-1.15)**** |
| F10-F19: mental and behavioural disorders due to psychoactive substance use | 27755 (10.46) | 2823 (9.36) | 24932 (10.60) | **0.87 (0.84-0.91)**** | **1.39 (1.31-1.48)**** |
| F20-F29:schizophrenia, schizotypal and delusional disorders | 18516 (6.98) | 2300 (7.62) | 16216 (6.90) | **1.11 (1.06-1.17)**** | **1.08 (1.01-1.15)*** |
| F30-F39: mood (affective) disorders | 42578 (16.05) | 5027 (16.66) | 37551 (15.97) | **1.05 (1.02-1.09)*** | **0.93 (0.88-0.97)*** |



For peer review only - http://bmjopen.bmj.com/site/about/guidelines.xhtml

| | | | | | |
|---|---|---|---|---|---|
| F40-F49: neurotic, stress-related and somatoform disorders | 33248 (12.53) | 2331 (7.73) | 30917 (13.15) | **0.55 (0.53-0.58)**** | **0.73 (0.69-0.78)**** |
| F50-F59: behavioural syndromes associated with physiological disturbances and physical factors | 8743 (3.30) | 148 (0.49) | 8595 (3.66) | **0.13 (0.11-0.15)**** | **0.47 (0.39-0.57)**** |
| F60-F69: disorders of adult personality and behaviour | 6945 (2.62) | 415 (1.38) | 6530 (2.78) | **0.49 (0.44-0.54)**** | 1.02 (0.91-1.15) |
| F70-F79: mental retardation | 3018 (1.14) | 311 (1.03) | 2707 (1.15) | 0.89 (0.79-1.01) | **1.24 (1.08-1.44)*** |
| F80-F89: disorders of psychological development | 7242 (2.73) | 54 (0.18) | 7188 (3.06) | **0.06 (0.04-0.07)**** | **0.65 (0.49-0.87)*** |
| F90-F98: behavioural and emotional disorders with onset usually occurring in childhood and adolescence | 17011 (6.41) | 77 (0.26) | 16934 (7.20) | **0.03 (0.03-0.04)**** | **0.45 (0.35-0.58)**** |
| Other diagnosis | 119638 (45.10) | 11014 (36.50) | 108624 (46.20) | **0.67 (0.65-0.69)**** | **0.65 (0.63-0.67)**** |
| Quartiles of face to face contacts: *n* (%) | | | | | |
| 1st (least face to face contact) | 76602 (28.87) | 5926 (19.64) | 70676 (30.06) | (reference) | (reference) |
| 2nd | 64914 (24.47) | 7767 (25.74) | 57147 (24.30) | **1.62 (1.56-1.68)**** | **1.10 (1.04-1.16)**** |
| 3rd | 59337 (22.37) | 8798 (29.16) | 50539 (21.49) | **2.08 (2.00-2.15)**** | **1.11 (1.05-1.17)**** |
| 4th (most face to face contact) | 64447 (24.29) | 7682 (25.46) | 56765 (24.14) | **1.61 (1.56-1.67)**** | 1.00 (0.95-1.06) |
| Inpatient bed days: *n* (%) | | | | | |
| None (0) | 238573 (89.93) | 26688 (88.45) | 211885 (90.12) | (reference) | (reference) |
| Low (1-2 days) | 1446 (0.55) | 102 (0.34) | 1344 (0.57) | **0.60 (0.49-0.74)**** | **1.52 (1.20-1.93)**** |
| Moderate (3-31 days) | 9825 (3.70) | 1034 (3.43) | 8791 (3.74) | **0.93 (0.87-1.00)*** | **1.58 (1.45-1.71)**** |
| High (32+ days) | 15456 (5.83) | 2349 (7.79) | 13107 (5.57) | **1.42 (1.36-1.49)**** | **1.59 (1.49-1.70)**** |

**Note.** *$p$<0.05. **$p$<0.001. [1]adjusted for all other variables listed in the table. Missing data: age (*n*=275); sex (*n*=55); ethnicity (*n*=28,727); resident in local catchment area (*n*=9,537); quartiles of neighbourhood deprivation (*n*=11,702); referral status (*n*=989).



For peer review only - http://bmjopen.bmj.com/site/about/guidelines.xhtml

**Table 5.** Logistic regression analysis examining socio-demographic and clinical associations with linked HES-ONS mortality in the matched cohort.

| Variable | Total Population (N=248,698) | Patient Deceased (*n*=28,161 11.32%) | Patient Alive (*n*=220,537 88.68%) | OR (95% CI) | aOR[1] (95% CI) | aOR[2] (95% CI) |
|---|---|---|---|---|---|---|
| **Sociodemographic variables** | | | | | | |
| Age: mean (SD) | 43.50 (22.79) | 77.75 (18.75) | 39.13 (19.28) | **1.091 (1.090-1.092)**** | **1.093 (1.092-1.095)**** | **1.092 (1.091-1.094)**** |
| Male Sex: *n* (%) | 123818 (49.79) | 13824 (49.09) | 109994 (49.88) | **0.97 (0.95-0.99)*** | **1.51 (1.45-1.56)**** | **1.50 (1.45-1.55)**** |
| Ethnicity coded: *n* (%) | | | | | | |
| British, Irish, or any other white ethnic groups | 131150 (58.93) | 20717 (78.96) | 110433 (56.26) | (reference) | (reference) | (reference) |
| Mixed | 6477 (2.91) | 180 (0.69) | 6297 (3.21) | **0.15 (0.13-0.18)**** | **0.67 (0.56-0.80)**** | **0.65 (0.54-0.79)**** |
| Indian, Pakistani, Bangladeshi, or 'other Asian' | 10155 (4.56) | 915 (3.49) | 9240 (4.71) | **0.53 (0.49-0.57)**** | **0.68 (0.62-0.74)**** | **0.67 (0.62-0.73)**** |
| Caribbean, African, or any 'other black' | 38013 (17.08) | 2611 (9.95) | 35402 (18.03) | **0.39 (0.38-0.41)**** | **0.60 (0.57-0.64)**** | **0.60 (0.57-0.63)**** |
| Other | 15393 (6.92) | 626 (2.39) | 14767 (7.52) | **0.23 (0.21-0.25)**** | **0.50 (0.45-0.55)**** | **0.50 (0.46-0.55)**** |
| Not stated | 21354 (9.60) | 1189 (4.53) | 20165 (10.27) | **0.31 (0.30-0.33)**** | 0.94 (0.87-1.02) | **0.92 (0.86-1.00)*** |
| Resident in SLaM catchment area: *n* (%) | 177766 (73.53) | 22329 (81.48) | 155437 (72.51) | **1.67 (1.62-1.72)**** | **0.84 (0.80-0.88)**** | **0.84 (0.80-0.88)**** |
| Quartiles of neighbourhood deprivation: *n* (%) | | | | | | |
| 1st (most deprived) | 60220 (25.09) | 7217 (26.42) | 53003 (24.92) | (reference) | (reference) | (reference) |
| 2nd | 59786 (24.91) | 6573 (24.06) | 53213 (25.02) | **0.91 (0.88-0.94)**** | **1.08 (1.03-1.13)*** | **1.08 (1.03-1.13)*** |
| 3rd | 60052 (25.02) | 6857 (25.10) | 53195 (25.01) | **0.95 (0.91-0.98)*** | **1.23 (1.17-1.30)**** | **1.23 (1.17-1.29)**** |
| 4th (least deprived) | 59973 (24.99) | 6667 (24.41) | 53306 (25.06) | **0.92 (0.89-0.95)**** | **1.30 (1.23-1.36)**** | **1.29 (1.23-1.36)**** |
| **Clinical variables** | | | | | | |
| Primary diagnosis ever[a]: *n* (%) | | | | | | |
| F00-F09: organic, including symptomatic, mental disorders | 24248 (9.75) | 11940 (42.40) | 12308 (5.58) | **12.45 (12.09-12.83)**** | **1.05 (1.00-1.11)*** | **1.06 (1.01-1.11)*** |
| F10-F19: mental and behavioural disorders due to psychoactive substance use | 26158 (10.52) | 2549 (9.05) | 23609 (10.71) | **0.83 (0.80-0.87)**** | **1.35 (1.27-1.44)**** | **1.36 (1.28-1.45)**** |

For peer review only



For peer review only - http://bmjopen.bmj.com/site/about/guidelines.xhtml

| | | | | | | |
|---|---|---|---|---|---|---|
| F20-F29:schizophrenia, schizotypal and delusional disorders | 17323 (6.97) | 2081 (7.39) | 15242 (6.91) | **1.07 (1.02-1.13)*** | 1.06 (0.99-1.14) | 1.05 (0.98-1.13) |
| F30-F39: mood (affective) disorders | 40515 (16.29) | 4715 (16.74) | 35800 (16.23) | **1.04 (1.00-1.07)*** | **0.92 (0.88-0.97)**** | **0.92 (0.88-0.97)*** |
| F40-F49: neurotic, stress-related and somatoform disorders | 31599 (12.71) | 2187 (7.77) | 29412 (13.34) | **0.55 (0.52-0.57)**** | **0.73 (0.68-0.77)**** | **0.72 (0.68-0.77)**** |
| F50-F59: behavioural syndromes associated with physiological disturbances and physical factors | 8388 (3.37) | 136 (0.48) | 8252 (3.74) | **0.12 (0.11-0.15)**** | **0.47 (0.38-0.57)**** | **0.48 (0.39-0.58)**** |
| F60-F69: disorders of adult personality and behaviour | 6585 (2.65) | 368 (1.31) | 6217 (2.82) | **0.46 (0.41-0.51)**** | 0.99 (0.88-1.13) | 0.98 (0.86-1.12) |
| F70-F79: mental retardation | 2898 (1.17) | 282 (1.00) | 2616 (1.19) | **0.84 (0.74-0.95)*** | **1.17 (1.01-1.37)*** | **1.17 (1.01-1.37)*** |
| F80-F89: disorders of psychological development | 6991 (2.81) | 47 (0.17) | 6944 (3.15) | **0.05 (0.04-0.07)**** | **0.60 (0.44-0.82)*** | **0.61 (0.45-0.83)*** |
| F90-F98: behavioural and emotional disorders with onset usually occurring in childhood and adolescence | 16363 (6.58) | 67 (0.24) | 16296 (7.39) | **0.03 (0.02-0.04)**** | **0.42 (0.32-0.55)**** | **0.43 (0.33-0.56)**** |
| Other diagnosis | 112556 (45.26) | 10275 (36.49) | 102281 (46.38) | **0.66 (0.65-0.68)**** | **0.64 (0.62-0.66)**** | **0.64 (0.62-0.66)**** |
| Quartiles of face to face contacts: *n* (%) | | | | | | |
| 1st (least face to face contact) | 70822 (28.48) | 5496 (19.52) | 65326 (29.62) | (reference) | (reference) | (reference) |
| 2nd | 59811 (24.05) | 7263 (25.79) | 52548 (23.83) | **1.64 (1.58-1.70)**** | **1.09 (1.03-1.15)*** | **1.09 (1.04-1.15)*** |
| 3rd | 56116 (22.56) | 8282 (29.41) | 47834 (21.69) | **2.06 (1.99-2.13)**** | **1.11 (1.05-1.17)**** | **1.11 (1.05-1.18)**** |
| 4th (most face to face contact) | 61949 (24.91) | 7120 (25.28) | 54829 (24.86) | **1.54 (1.49-1.60)**** | 0.99 (0.93-1.05) | 1.00 (0.94-1.06) |
| Inpatient bed days: *n* (%) | | | | | | |
| None (0) | 223527 (89.88) | 24994 (88.75) | 198533 (90.02) | (reference) | (reference) | (reference) |
| Low (1-2 days) | 1342 (0.54) | 86 (0.31) | 1256 (0.57) | **0.54 (0.44-0.68)**** | **1.40 (1.09-1.81)*** | **1.36 (1.04-1.78)*** |
| Moderate (3-31 days) | 9133 (3.67) | 939 (3.33) | 8194 (3.72) | **0.91 (0.85-0.98)*** | **1.54 (1.41-1.68)**** | **1.53 (1.40-1.67)**** |
| High (32+ days) | 14696 (5.91) | 2142 (7.61) | 12554 (5.69) | **1.36 (1.29-1.42)**** | **1.55 (1.45-1.66)**** | **1.55 (1.44-1.66)**** |
| Optimal Match | 203552 (84.15) | 178731 (87.81) | 24821 (12.19) | **0.63 (0.61-0.66)**** | ----- | **0.85 (0.81-0.90)**** |

**Note.** *$p<0.05$. **$p<0.001$. [1]adjusted for all other variables listed in the table (except optimal match). [2]adjusted for optimal match rank and inverse probability weighting for matching. Missing data: sex (*n*=37); ethnicity (*n*=26,156); resident in local catchment area (*n*=6,937); quartiles of neighbourhood deprivation (*n*=8,667); optimal match (*n*=6,808).



For peer review only - http://bmjopen.bmj.com/site/about/guidelines.xhtml

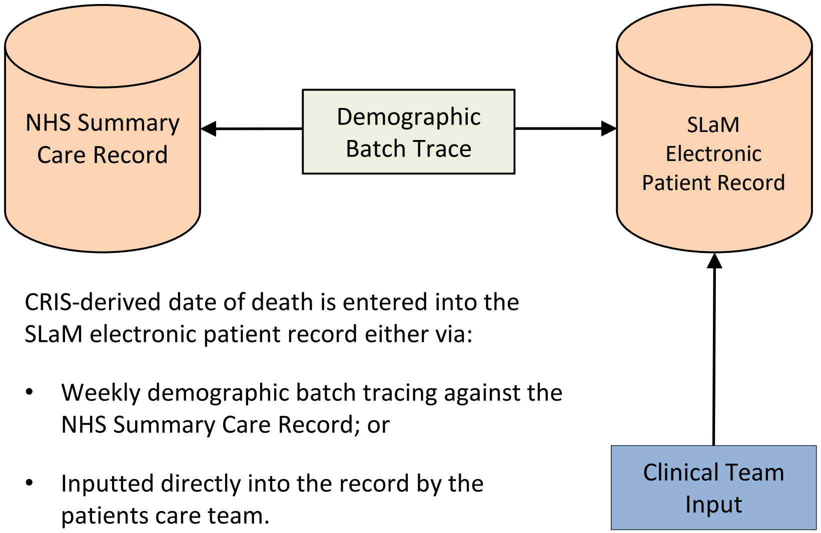
NHS Summary Care Record
Demographic Batch Trace
SLaM Electronic Patient Record
CRIS-derived date of death is entered into the SLaM electronic patient record either via:
• Weekly demographic batch tracing against the NHS Summary Care Record; or
• Inputted directly into the record by the patients care team.
Clinical Team Input

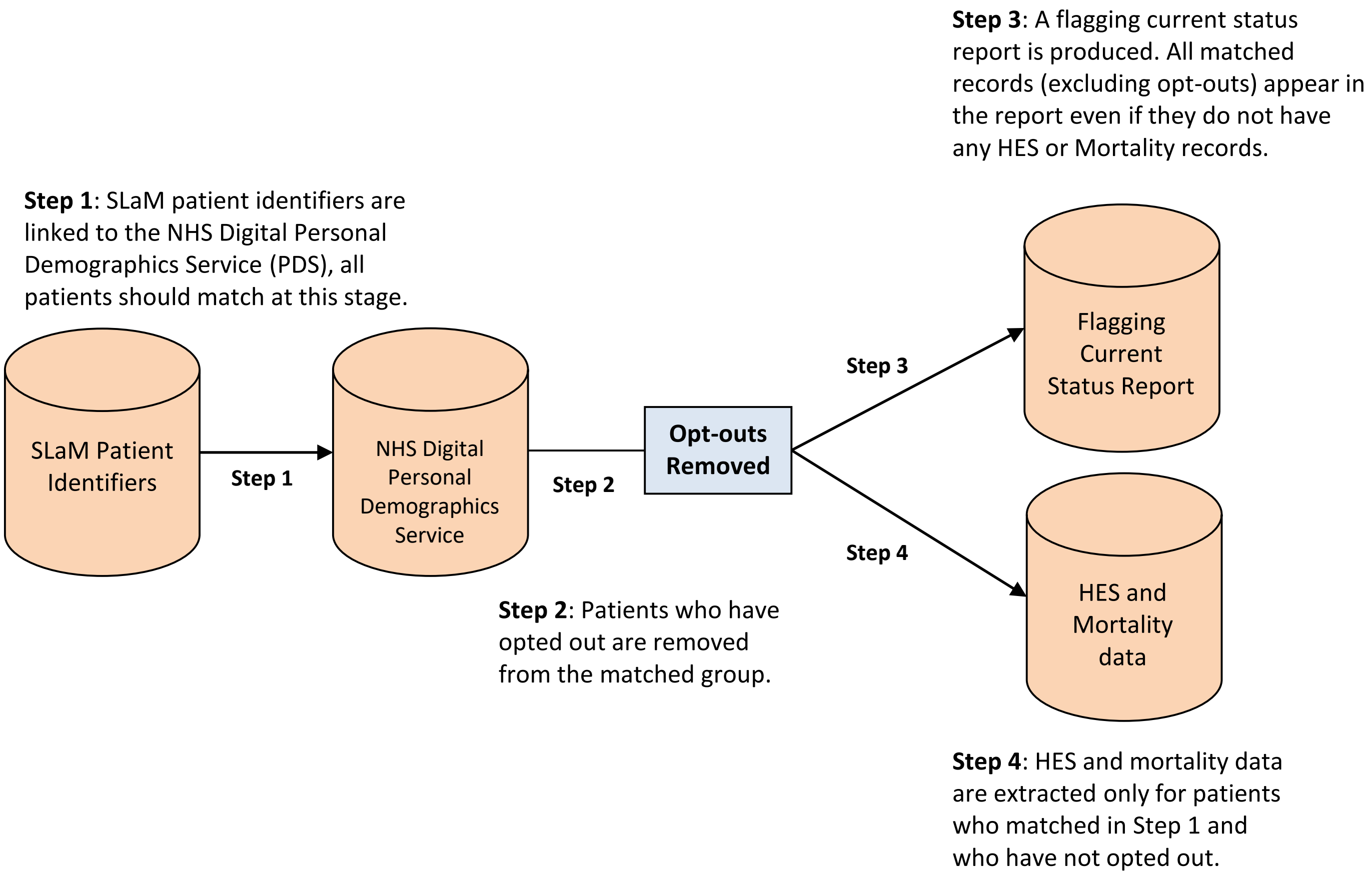
Step 1: SLaM patient identifiers are linked to the NHS Digital Personal Demographics Service (PDS), all patients should match at this stage.
SLaM Patient Identifiers
Step 1
NHS Digital Personal Demographics Service
Step 2
Opt-outs Removed
Step 2: Patients who have opted out are removed from the matched group.
Step 3
Step 3: A flagging current status report is produced. All matched records (excluding opt-outs) appear in the report even if they do not have any HES or Mortality records.
Flagging Current Status Report
Step 4
HES and Mortality data
Step 4: HES and mortality data are extracted only for patients who matched in Step 1 and who have not opted out.

**Table 1.** STROBE 2007 (v4) Statement: The Impact of Matching Error on Linked Mortality Outcome in a Data Linkage of Secondary Mental Health Data with Hospital Episode Statistics (HES) and Mortality Records in South East London: A Cross-Sectional Study

| Section/Topic | Item # | Recommendation | Reported on page # |
|---|---|---|---|
| **Title and abstract** | 1 | (*a*) Indicate the study's design with a commonly used term in the title or the abstract | 1 |
| | | (*b*) Provide in the abstract an informative and balanced summary of what was done and what was found | 2 |
| **Introduction** | | | |
| Background/rationale | 2 | Explain the scientific background and rationale for the investigation being reported | 4 |
| Objectives | 3 | State specific objectives, including any prespecified hypotheses | 4/5 |
| **Methods** | | | |
| Study design | 4 | Present key elements of study design early in the paper | 5 |
| Setting | 5 | Describe the setting, locations, and relevant dates, including periods of recruitment, exposure, follow-up, and data collection | 5/6 |
| Participants | 6 | (*a*) Give the eligibility criteria, and the sources and methods of selection of participants | 5 |
| Variables | 7 | Clearly define all outcomes, exposures, predictors, potential confounders, and effect modifiers. Give diagnostic criteria, if applicable | 5/24 |
| Data sources/ measurement | 8* | For each variable of interest, give sources of data and details of methods of assessment (measurement). Describe comparability of assessment methods if there is more than one group | 5/6 |
| Bias | 9 | Describe any efforts to address potential sources of bias | n/a |
| Study size | 10 | Explain how the study size was arrived at | 5 |
| Quantitative variables | 11 | Explain how quantitative variables were handled in the analyses. If applicable, describe which groupings were chosen and why | 24 |
| Statistical methods | 12 | (*a*) Describe all statistical methods, including those used to control for confounding | 7 |
| | | (*b*) Describe any methods used to examine subgroups and interactions | 7 |
| | | (*c*) Explain how missing data were addressed | n/a |
| | | (*d*) If applicable, describe analytical methods taking account of sampling strategy | n/a |
| | | (*e*) Describe any sensitivity analyses | 7 |

For peer review only

| | | | |
|---|---|---|---|
| **Results** | | | |
| Participants | 13* | (a) Report numbers of individuals at each stage of study—eg numbers potentially eligible, examined for eligibility, confirmed eligible, included in the study, completing follow-up, and analysed | 8 |
| | | (b) Give reasons for non-participation at each stage | n/a |
| | | (c) Consider use of a flow diagram | n/a |
| Descriptive data | 14* | (a) Give characteristics of study participants (eg demographic, clinical, social) and information on exposures and potential confounders | 8 |
| | | (b) Indicate number of participants with missing data for each variable of interest | 28/30/32 |
| Outcome data | 15* | Report numbers of outcome events or summary measures | 8/9/10 |
| Main results | 16 | (*a*) Give unadjusted estimates and, if applicable, confounder-adjusted estimates and their precision (eg, 95% confidence interval). Make clear which confounders were adjusted for and why they were included | 8/9/10/28/30/32 |
| | | (*b*) Report category boundaries when continuous variables were categorized | n/a |
| | | (*c*) If relevant, consider translating estimates of relative risk into absolute risk for a meaningful time period | n/a |
| Other analyses | 17 | Report other analyses done—eg analyses of subgroups and interactions, and sensitivity analyses | 8/9/10/11 |
| **Discussion** | | | |
| Key results | 18 | Summarise key results with reference to study objectives | 11/12 |
| Limitations | 19 | Discuss limitations of the study, taking into account sources of potential bias or imprecision. Discuss both direction and magnitude of any potential bias | 13/14 |
| Interpretation | 20 | Give a cautious overall interpretation of results considering objectives, limitations, multiplicity of analyses, results from similar studies, and other relevant evidence | 11/12 |
| Generalisability | 21 | Discuss the generalisability (external validity) of the study results | 13/14 |
| **Other information** | | | |
| Funding | 22 | Give the source of funding and the role of the funders for the present study and, if applicable, for the original study on which the present article is based | 15 |

*Give information separately for cases and controls in case-control studies and, if applicable, for exposed and unexposed groups in cohort and cross-sectional studies.

For peer review only

**Table 2.** Logistic regression analysis examining administrative, socio-demographic, and clinical associations with an optimal match.

| Variable | Total Population (N=241,890) | Optimal Match (*n*=203,552 84.15%) | Non-optimal Match (*n*=38,338 15.85%) | OR (95% CI) | aOR[1] (95% CI) |
|---|---|---|---|---|---|
| **Sociodemographic variables** | | | | | |
| Age: mean (SD) | 43.70 (22.89) | 44.68 (23.01) | 38.47 (21.52) | **0.987 (0.987-0.988)**** | **0.985 (0.984-0.986)**** |
| Male Sex: *n* (%) | 119634 (49.46) | 99187 (48.73) | 20447 (53.38) | **1.20 (1.18-1.23)**** | **1.14 (1.12-1.17)**** |
| Patient deceased: *n* (%) | 28790 (11.90) | 25629 (12.59) | 3161 (8.25) | **0.63 (0.61-0.65)**** | 0.95 (0.91-1.00) |
| Ethnicity coded: *n* (%) | | | | | |
| British, Irish, or any other white ethnic groups | 128547 (59.32) | 109139 (59.71) | 19408 (57.25) | (reference) | (reference) |
| Mixed | 6306 (2.91) | 5168 (2.83) | 1138 (3.36) | **1.24 (1.16-1.32)**** | 1.00 (0.93-1.07) |
| Indian, Pakistani, Bangladeshi, or 'other Asian' | 9745 (4.50) | 8348 (4.57) | 1397 (4.12) | **0.94 (0.89-0.99)*** | **0.90 (0.85-0.96)*** |
| Caribbean, African, or any 'other black' | 37052 (17.10) | 31115 (17.02) | 5937 (17.51) | **1.07 (1.04-1.11)**** | 0.97 (0.93-1.00) |
| Other | 14728 (6.80) | 12248 (6.70) | 2480 (7.32) | **1.14 (1.09-1.19)**** | **1.08 (1.03-1.13)*** |
| Not stated | 20319 (9.38) | 16778 (9.18) | 3541 (10.45) | **1.19 (1.14-1.23)**** | **1.04 (1.00-1.09)*** |
| Resident in SLaM catchment area: *n* (%) | 172567 (73.34) | 145594 (73.34) | 26973 (73.37) | 1.00 (0.98-1.03) | **1.13 (1.09-1.16)**** |
| Quartiles of neighbourhood deprivation: *n* (%) | | | | | |
| 1st (most deprived) | 58789 (25.15) | 49830 (25.24) | 8959 (24.67) | (reference) | (reference) |
| 2nd | 58146 (24.87) | 48785 (24.71) | 9361 (25.78) | **1.07 (1.03-1.10)**** | 1.01 (0.98-1.05) |
| 3rd | 58342 (24.96) | 49200 (24.92) | 9142 (25.18) | **1.03 (1.00-1.07)*** | 0.97 (0.94-1.01) |
| 4th (least deprived) | 58488 (25.02) | 49638 (25.14) | 8850 (24.37) | 0.99 (0.96-1.02) | **0.92 (0.89-0.96)**** |
| **Clinical variables** | | | | | |
| Referral status in past 2 years: *n* (%) | | | | | |
| Accepted | 11061 (4.59) | 9445 (4.65) | 1616 (4.23) | (reference) | (reference) |
| Discharged | 41227 (17.09) | 35007 (17.25) | 6220 (16.27) | 1.04 (0.98-1.10) | **1.11 (1.04-1.18)*** |
| Rejected | 8559 (3.55) | 7060 (3.48) | 1499 (3.92) | **1.24 (1.06-1.18)**** | **1.10 (1.01-1.21)*** |
| No referral in 2 year prior to linkage | 180330 (74.77) | 151442 (74.62) | 28888 (75.58) | **1.11 (1.06-1.18)**** | **1.24 (1.17-1.32)**** |
| Primary diagnosis ever: *n (%)* | | | | | |
| F00-F09: organic, including symptomatic, mental disorders | 24095 (9.96) | 21191 (10.41) | 2904 (7.57) | **0.71 (0.68-0.73)**** | **1.31 (1.24-1.39)**** |
| F10-F19: mental and behavioural disorders due to psychoactive substance use | 25530 (10.55) | 21124 (10.38) | 4406 (11.49) | **1.12 (1.08-1.16)**** | **1.17 (1.12-1.22)**** |
| F20-F29:schizophrenia, schizotypal and delusional disorders | 16889 (6.98) | 14328 (7.04) | 2561 (6.68) | **0.95 (0.91-0.99)*** | 1.05 (0.99-1.11) |

For peer review only

| | | | | | |
|---|---|---|---|---|---|
| F30-F39: mood (affective) disorders | 39799 (16.45) | 34512 (16.95) | 5287 (13.79) | **0.78 (0.76-0.81)**** | **0.88 (0.85-0.92)**** |
| F40-F49: neurotic, stress-related and somatoform disorders | 30943 (12.79) | 26729 (13.13) | 4214 (10.99) | **0.82 (0.79-0.85)**** | **0.83 (0.79-0.86)**** |
| F50-F59: behavioural syndromes associated with physiological disturbances and physical factors | 8086 (3.34) | 6634 (3.26) | 1452 (3.79) | **1.17 (1.10-1.24)**** | **1.12 (1.05-1.19)*** |
| F60-F69: disorders of adult personality and behaviour | 6509 (2.69) | 5521 (2.71) | 988 (2.58) | 0.95 (0.89-1.02) | 0.98 (0.91-1.05) |
| F70-F79: mental retardation | 2810 (1.16) | 2448 (1.20) | 362 (0.94) | **0.78 (0.70-0.87)**** | **0.82 (0.73-0.92)**** |
| F80-F89: disorders of psychological development | 6804 (2.81) | 5673 (2.79) | 1131 (2.95) | 1.06 (0.99-1.13) | **0.76 (0.70-0.81)**** |
| F90-F98: behavioural and emotional disorders with onset usually occurring in childhood and adolescence | 15931 (6.59) | 12877 (6.33) | 3054 (7.97) | **1.28 (1.23-1.34)**** | 0.96 (0.91-1.00) |
| Other diagnosis | 109854 (45.41) | 92623 (45.50) | 17231 (44.94) | **0.98 (0.96-1.00)*** | **0.96 (0.94-0.99)**** |
| Quartiles of face to face contacts: *n* (%) | | | | | |
| 1st (least face to face contact) | 68150 (28.17) | 56507 (27.76) | 11643 (30.37) | (reference) | (reference) |
| 2nd | 58128 (24.03) | 48702 (23.93) | 9426 (24.59) | **0.94 (0.91-0.97)**** | 1.01 (0.97-1.04) |
| 3rd | 54781 (22.65) | 46311 (22.75) | 8470 (22.09) | **0.89 (0.86-0.92)**** | **0.95 (0.91-0.99)*** |
| 4th (most face to face contact) | 60831 (25.15) | 52032 (25.56) | 8799 (22.95) | **0.82 (0.80-0.85)**** | **0.89 (0.85-0.93)**** |
| Inpatient bed days: *n* (%) | | | | | |
| None (0) | 216890 (89.66) | 182362 (89.59) | 34528 (90.06) | (reference) | (reference) |
| Low (1-2 days) | 1327 (0.55) | 1106 (0.54) | 221 (0.58) | 1.06 (0.91-1.22) | 1.06 (0.91-1.24) |
| Moderate (3-31 days) | 9064 (3.75) | 7607 (3.74) | 1457 (3.80) | 1.01 (0.96-1.07) | 1.05 (0.98-1.12) |
| High (32+ days) | 14609 (6.04) | 12477 (6.13) | 2132 (5.56) | **0.90 (0.86-0.95)**** | 1.00 (0.95-1.06) |

**Note.** Optimal match data missing for *n*=6,808 (2.75%) matched records. **p*<0.05. ***p*<0.001. [1]adjusted for all other variables listed in the table. Missing data: sex (*n*=34); ethnicity (*n*=25,193); resident in local catchment area (*n*=6,604); quartiles of neighbourhood deprivation (*n*=8,125); referral status (*n*=713).

For peer review only

**Table 3.** Logistic regression analysis examining the association between sociodemographic factors and availability of NHS number for matching.

| Variable | Total Population (N=265,300) | NHS Number Present (*n*=259,545 97.83%) | NHS Number Missing (*n*=5,755 2.17%) | OR (95% CI) | aOR[1] (95% CI) |
|---|---|---|---|---|---|
| **Sociodemographic variables** | | | | | |
| Age: mean (SD) | 43.40 (22.69) | 43.49 (22.79) | 39.19 (16.57) | **1.009 (1.008-1.010)**** | **1.010 (1.008-1.012)**** |
| Male Sex: *n* (%) | 132730 (50.04) | 126085 (49.74) | 3645 (63.47) | **0.57 (0.54-0.60)**** | **0.60 (0.56-0.66)**** |
| Ethnicity: *n* (%) | | | | | |
| British, Irish, or any other white ethnic groups | 138495(58.54) | 136495 (58.69) | 2265 (50.99) | (reference) | (reference) |
| Mixed | 6853 (2.90) | 6782 (2.92) | 71 (1.60) | **1.59 (1.25-2.01)**** | **1.60 (1.16-2.19)*** |
| Indian, Pakistani, Bangladeshi, or 'other Asian' | 10889 (4.60) | 10586 (4.56) | 303 (6.82) | **0.58 (0.51-0.66)**** | **0.44 (0.38-0.51)**** |
| Caribbean, African, or 'other black' | 40725 (17.21) | 40008 (17.24) | 717 (16.14) | 0.93 (0.85-1.01) | **0.73 (0.65-0.82)**** |
| Other | 16650 (7.04) | 16120 (6.94) | 530 (11.93) | **0.51 (0.46-0.56)**** | **0.45 (0.39-0.51)**** |
| Not stated | 22961 (9.71) | 22405 (9.65 | 556 (12.52) | **0.67 (0.61-0.74)**** | **0.58 (0.51-0.65)**** |
| Resident in SLaM Catchment Area: *n* (%) | 187773 (73.42) | 185393 (73.51) | 2380 (66.93) | **1.37 (1.28-1.47)**** | **1.15 (1.04-1.26)*** |
| Quartiles of neighbourhood deprivation: *n* (%) | | | | | |
| 1st (most deprived) | 63476 (25.03) | 62832 (25.10) | 644 (19.93) | (reference) | (reference) |
| 2nd | 63452 (25.02) | 62605 (25.01) | 847 (26.21) | **0.76 (0.68-0.84)**** | **0.66 (0.59-0.75)**** |
| 3rd | 63449 (25.02) | 62557 (24.99) | 892 (27.60) | **0.72 (0.65-0.80)**** | **0.68 (0.60-0.77)**** |
| 4th (least deprived) | 63221 (24.93 | 62371 (24.91) | 849 (26.27) | **0.75 (0.68-0.83)**** | **0.70 (0.61-0.79)**** |

**Note.** **p*<0.05. ***p*<0.001. [1]adjusted for all other variables listed in the table. Missing data: age (*n*=275); sex (*n*=55); ethnicity (*n*=28,727); quartiles of neighbourhood deprivation (*n*=11,702).

For peer review only

**Table 4.** Logistic regression analysis examining the association between sociodemographic factors and availability of date of birth for matching.

| Variable | Total Population (N=265,300) | Date of Birth Present (*n*=265,010 99.89%) | Date of Birth Missing (*n*=290 0.11%) | OR (95% CI) | aOR[1] (95% CI) |
|---|---|---|---|---|---|
| **Sociodemographic variables** | | | | | |
| Age: mean (SD) | 43.40 (22.69) | 43.40 (22.69) | 32.93 (20.49) | 1.025 (0.997-1.054) | 1.033 (0.974-1.096) |
| Male Sex: *n* (%) | 132730 (50.04) | 132585 (50.04) | 145 (50.17) | 0.99 (0.79-1.25) | 1.78 (0.29-10.77) |
| Ethnicity: *n* (%) | | | | | |
| British, Irish, or any other white ethnic groups | 138495(58.54) | 138440 (58.55) | 55 (50.00) | (reference) | (reference) |
| Mixed | 6853 (2.90) | 6853 (2.90) | 0 (0.00) | ---- | ---- |
| Indian, Pakistani, Bangladeshi, or 'other Asian' | 10889 (4.60) | 10877 (4.60) | 12 (10.91) | **0.36 (0.19-0.67)*** | 0.08 (0.01-1.22) |
| Caribbean, African, or 'other black' | 40725 (17.21) | 40710 (17.22) | 15 (13.64) | 1.08 (0.61-1.91) | ---- |
| Other | 16650 (7.04) | 16632 (7.03) | 18 (16.36) | **0.37 (0.22-0.63)**** | 0.10 (0.01-1.73) |
| Not stated | 22961 (9.71) | 22951 (9.71) | 10 (9.09) | 0.91 (046-1.79) | 0.11 (0.01-1.23) |
| Resident in SLaM Catchment Area: *n* (%) | 187773(73.42) | 187661 (73.42) | 112 (73.68) | 0.99 (0.69-1.42) | 3.23 (0.46-22.53) |
| Quartiles of neighbourhood deprivation: *n* (%) | | | | | |
| 1st (most deprived) | 63476 (25.03) | 63428 (25.03) | 48 (33.33) | (reference) | (reference) |
| 2nd | 63452 (25.02) | 63427 (25.03) | 25 (17.36) | **1.92 (1.18-3.11)*** | 1.43 (0.12-16.75) |
| 3rd | 63449 (25.02) | 63405 (25.02) | 44 (30.56) | 1.09 (0.72-1.64) | 1.26 (0.10-15.36) |
| 4th (least deprived) | 63221 (24.93 | 63194 (24.93) | 27 (18.75) | **1.77 (1.11-2.84)*** | 1.23 (0.10-15.00) |

**Note.** **p*<0.05. ***p*<0.001. [1]adjusted for all other variables listed in the table. Missing data: age (*n*=275); sex (*n*=55); ethnicity (*n*=28,727); quartiles of neighbourhood deprivation (*n*=11,702).

For peer review only
